# Spatiotemporal Modulation of Flavonoid Metabolism in Blueberries

**DOI:** 10.3389/fpls.2020.00545

**Published:** 2020-05-13

**Authors:** Catrin Sonja Günther, Andrew P. Dare, Tony K. McGhie, Cecilia Deng, Declan J. Lafferty, Blue J. Plunkett, Ella R. P. Grierson, Janice L. Turner, Laura Jaakola, Nick W. Albert, Richard V. Espley

**Affiliations:** ^1^The New Zealand Institute for Plant & Food Research Ltd., Auckland, New Zealand; ^2^The New Zealand Institute for Plant & Food Research Ltd., Palmerston North, New Zealand; ^3^School of Biological Sciences, The University of Auckland, Auckland, New Zealand; ^4^The New Zealand Institute for Plant & Food Research Ltd., Brooklyn, New Zealand; ^5^Climate Laboratory Holt, Department of Arctic and Marine Biology, UiT The Arctic University of Norway, Tromsø, Norway; ^6^Norwegian Institute of Bioeconomy Research, Norwegian Institute of Bioeconomy Research, Ås, Norway

**Keywords:** anthocyanin, blueberry, flavonoid, fruit maturation, MYB, secondary metabolism, structural genes, phenylpropanoid pathway

## Abstract

Blueberries are distinguished by their purple-blue fruit color, which develops during ripening and is derived from a characteristic composition of flavonoid-derived anthocyanin pigments. The production of anthocyanins is confined to fruit skin, leaving the colorless fruit flesh devoid of these compounds. By linking accumulation patterns of phenolic metabolites with gene transcription in Northern Highbush (*Vaccinium corymbosum*) and Rabbiteye (*Vaccinium virgatum)* blueberry, we investigated factors limiting anthocyanin production in berry flesh. We find that flavonoid production was generally lower in fruit flesh compared with skin and concentrations further declined during maturation. A common set of structural genes was identified across both species, indicating that tissue-specific flavonoid biosynthesis was dependent on co-expression of multiple pathway genes and limited by the phenylpropanoid pathway in combination with *CHS*, *F3H*, and *ANS* as potential pathway bottlenecks. While metabolite concentrations were comparable between the blueberry genotypes when fully ripe, the anthocyanin composition was distinct and depended on the degree of hydroxylation/methoxylation of the anthocyanidin moiety in combination with genotype-specific glycosylation patterns. Co-correlation analysis of phenolic metabolites with pathway structural genes revealed characteristic isoforms of *O*-methyltransferases and UDP-glucose:flavonoid-3-*O*-glycosyltransferase that were likely to modulate anthocyanin composition. Finally, we identified candidate transcriptional regulators that were co-expressed with structural genes, including the activators *MYBA*, *MYBPA1*, and *bHLH2* together with the repressor *MYBC2*, which suggested an interdependent role in anthocyanin regulation.

## Introduction

Blueberries have gained global popularity as high-value fruit and whilst all cultivated species originated from North America, production is growing globally. Northern Highbush (*Vaccinium corymbosum*) and Rabbiteye (*Vaccinium virgatum*, syn. *ashei*) are the economically most important species although blueberries are usually marketed without reference to species or cultivar. Rabbiteye cultivation is increasing in warmer climates where it was shown to outperform *V. corymbosum* genotypes (Huang and Li, 2015; Medeiros et al., 2018). In addition to Rabbiteye’s adaptability to warmer climates, its late flowering season make a valuable extension to the fruit harvest window when combined with Northern Highbush (Scalzo, 2013), thus furthering co-cultivation of these complementary species.

The striking purple/blue skin color is a defining character of blueberries, arising from high concentrations of anthocyanins. Anthocyanins are flavonoids that provide pigmentation to flowers and fruits of many plant species, acting as visual cues to attract pollinators and seed-distributers (Davies et al., 2012). In many fruits, including blueberries, anthocyanins are produced during ripening when fruit have the greatest reward, linking visual attraction with nutritional value. Visual traits like color and appearance are likely the first criteria for assessing blueberry quality prior to consumption and consumer demands on fruit quality traits drive current breeding targets and profitability (Gilbert et al., 2014; Gallardo et al., 2018). The public messaging surrounding blueberries as “superfoods,” containing high concentrations of “antioxidants,” is recognized by consumers and influences their fruit preferences. While anthocyanin profiles of Northern Highbush genotypes are well researched, comparative studies using Rabbiteye are limited but suggest differences in both anthocyanin content and composition between species when ripe (Lohachoompol et al., 2008; Timmers et al., 2017).

Structurally, anthocyanins are composite molecules consisting of an anthocyanidin (aglycone) moiety linked to a hexoside or pentoside. The coloring of the anthocyanidin varies with the degree and position of hydroxylation and/or methoxylation of their 2-phenyl-ring structure in combination with pH and the presence of co-pigments such as flavonols. In nature, over 500 different anthocyanin structures have been reported, based on over a dozen different anthocyanidins (De Pascual-Teresa and Sanchez-Ballesta, 2008). Amongst these, cyanidin, peonidin, delphinidin, malvidin, and petunidin are most commonly identified in blueberry fruit (Kalt et al., 1999; Lohachoompol et al., 2008). While cyanidin-3-*O*-glycosides (Kong et al., 2003; Fang, 2015), are the predominant anthocyanins in many fruits, delphinidin- and malvidin-3-*O*-glycosides are prevalent at high concentrations in blueberries (Kalt et al., 1999; Veberic et al., 2015), which is uncommon among berries (Suh et al., 2018).

**FIGURE 1:**
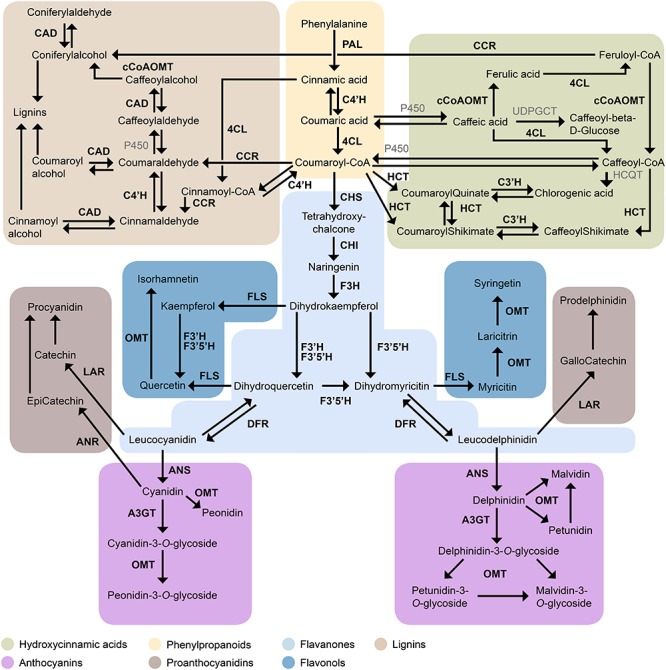
Overview of the metabolic network leading to flavonoid biosynthesis in blueberries. Enzyme abbreviations as follows; PAL, phenylalanine ammonia lyase; C4′H, cinnamate 4-hydroxylase; 4CL, 4-coumarate-CoA ligase; CCR, cinnamoyl-CoA reductase; CAD, cinnamoyl alcohol dehydrogenase; cCoAOMT, Caffeoyl-CoA *O*-methyltransferase; HCT, shikimate *O*-hydroxycinnamoyltransferase; C3′H, 5-*O*-(4-coumaroyl)-D-quinate 3′-monooxygenase; CHS, chalcone synthase; CHI, chalcone isomerase; F3H, flavanone 3-dioxygenase; F3′H, flavonoid 3′ hydroxylase; F3′5′H, flavonoid 3′, 5′ hydroxylase; FLS, flavonol synthase; OMT, *O*-methyltransferase; LAR, leucoanthocyanidin reductase; ANR, anthocyanidin reductase; DFR, dihydroflavonol 4-reductase; ANS, anthocyanidin synthase; A3GT, anthocyanidin 3-*O*-glycosyltransferase.

In addition to colorful anthocyanins, blueberry fruits also contain complex profiles of mainly colorless flavonoids, which share precursors with anthocyanins (Figure 1). In brief, phenylalanine is transformed into *p*-coumaroyl-CoA by the concerted action of phenylalanine ammonia lyase (PAL), cinnamic acid 4-hydroxylase (C4′H), and 4-coumarate ligase (4CL), known as the phenylpropanoid pathway. The thioester *p*-coumaroyl-CoA is a primary precursor to a range of polyphenolic compounds (Durazzo et al., 2019), namely lignans, coumarins, hydroxycinnamic acids (HCAs), and flavonoids (see Quideau et al., 2011 for definition on polyphenols). Anthocyanins are derived from the flavonoid pathway, together with chalcones, flavones, flavonols, and flavanols (Winkel-Shirley, 2001). The first enzyme specific to the flavonoid pathway is chalcone synthase (CHS). This is followed by a cyclisation step by chalcone isomerase (CHI) and hydroxylation by flavanone 3-hydroxylase (F3H). From here the pathway branches according to the hydroxylation pattern, to produce either di- (flavonoid 3′ hydroxylase, F3′H) or tri-hydroxylated (flavonoid 3′5′ hydroxylase, F3′5′H) dihydroflavanonols, respectively. These precursors are converted to flavonols via flavonol synthase (FLS) or reduced to leucoanthocyanidin, a precursor to both anthocyanidins and proanthocyanidins (PAC), by dihydroflavonol reductase (DFR). Blueberry PACs, are polymers of the flavanols, catechin, epicatechin, and/or gallocatechin. Leucoanthocyanidin reductase (LAR) converts leucocyanidin and leucodelphinidin into catechin and gallocatechin, respectively, while epicatechin is derived from cyanidin via anthocyanidin reductase (ANR). Although delphinidin can be converted into epigallocatechin by ANR (Shi et al., 2018), this flavanol is not usually detected in fruit of Northern Highbush or Rabbiteye blueberry. PACs are often localized to vegetative tissues, the seed coat and unripe fruit (Xu et al., 2015).

The biosynthesis of flavonoids is regulated at the transcriptional level and distinct control over the production of anthocyanins and PACs is proposed as regulated by an “MBW” transcription factor (TF) complex. This complex consists of R2R3 MYB, bHLH and WD-Repeat proteins which were shown to activate the expression of biosynthetic genes (Baudry et al., 2004; González et al., 2008; Albert et al., 2014). Recently, we isolated and characterized an R2R3-MYB gene from blueberry, *VcMYBA* (Plunkett et al., 2018). *VcMYBA* was coexpressed with anthocyanin biosynthetic genes and induced anthocyanin production when expressed *in planta*. However, an additional class of R2R3-MYB protein, exemplified by grape *VviMYBPA1* also regulates anthocyanin and/or PAC biosynthesis in a variety of berries (Bogs et al., 2007; Jaakola et al., 2010; Primetta et al., 2015), although the exact role remains unclear.

While the majority of plant MYBs are involved in transcriptional activation, MYBs with repressive activity also regulate flavonoid biosynthesis. R2R3 and R3 MYB repressors such as AmMYB308, and PhMYBx, repress key branch-points within the pathway by directly or indirectly disrupting the activity of the MBW complex redirecting metabolites to alternative biosynthetic pathways (Tamagnone et al., 1998; Jin et al., 2000; Colquhoun et al., 2011; Albert et al., 2014). These repressors are themselves regulated by the MBW activation complex, providing a feedback repression mechanism (Albert et al., 2014; Zhou et al., 2015; Bond et al., 2016).

Different phases of blueberry fruit development are commonly based on berry expansion and color change (Zifkin et al., 2012; Karppinen et al., 2016; Li et al., 2019b) and it is well established that anthocyanins accumulate in parallel with the expression of structural genes of the flavonoid pathway. Blueberry pigmentation, however, is confined to fruit skin and since our current knowledge is based on whole fruit studies, we do not know whether this is regulated at key points within the pathway or by reduced flavonoid biosynthesis overall. Different mechanisms were suggested to limit anthocyanin production in pigment-deficient genotypes, such as inactivation of anthocyanidin synthase (ANS) in raspberry (Rafique et al., 2016), substrate competition between DFR and FLS in *Petunia* (Davies et al., 2003) and downregulation of multiple structural genes in albino bilberry (Zorenc et al., 2017). To investigate the determinants of spatial anthocyanin production in wild-type blueberries, we employed an interdisciplinary approach, linking targeted analysis of metabolites and gene transcription, to map the dynamic changes in flavonoid biosynthesis during fruit maturation of the commercial cultivars ‘Nui’ (Northern Highbush) and ‘Velluto Blue’ (Rabbiteye). Further, we studied how structural genes and their transcriptional regulators affect genotypic flavonoid profiles as a prerequisite for identifying genetic targets that modulate color-related traits.

## Materials and Methods

### Blueberry Fruit Material

Berries were derived from cultivated collections of tetraploid *V. corymbosum* ‘Nui’ and hexaploid *V. virgatum* ‘Velluto Blue’ (Plant & Food Research, Motueka, New Zealand). Fruit was harvested between November 2017 and March 2018 at five different developmental stages identified by fruit skin color ranging from green to deep purple (Zifkin et al., 2012). The sampling was replicated at three separate plots within the field (each “plot” comprised two plants). Each biological replicate consisted of approximately 40 berries from each plot per stage and blueberry cultivar. Each replicate was immediately frozen in dry ice and then transferred to a −80°C freezer. Berry skin was separated from the flesh with a scalpel while keeping the fruit frozen on foil-covered dry ice. Seeds were not removed from the fruit flesh. Each frozen tissue sample was homogenized to a fine powder (IKA A11 basic mill) and stored at −80°C for no longer than 3 months.

### Extraction of Polyphenols and UHPLC-ESI-QTOF-HRMS

Each sample was freeze dried (Edwards worldwide) and 50 ± 5 mg was extracted with 1.5 mL solvent (ethanol: water: formic acid; 80:20:1) at room temperature for 2.5–3 h in the dark and vortexed several times during this period. The samples were then transferred to 1°C overnight, centrifuged (10.000*g* for 10 min) then diluted (1:10) with 1% formic acid in methanol and stored at −20°C prior to analysis.

Ultra-High-Performance Liquid Chromatography (UHPLC) – Mass Spectrometry (LC-MS) was employed (Henry-Kirk et al., 2018) to measure anthocyanins and polyphenols and data were processed using Target Analysis for Screening and Quantitation software (TASQ, Bruker Daltonics, Bremen, Germany).

#### Anthocyanins

Anthocyanins were separated using a Luna Omega Polar C18 (100 × 2.1 mm, 1.6 μm) column maintained at 50°C. The mobile phase was composed of solvents: A = 5% formic acid in water and B = 100% acetonitrile at a flow rate of 300 μL/min. The solvent gradient was: initial composition 95% A, 0–0.5 min; linear gradient to 85% A, 0.5–10 min; linear gradient to 60% A, 10–20 min; linear gradient to 5% A, 20–25 min; composition held at 95% A, 25–28 min; linear gradient to 95% A, 28–28.2 min; to return to the initial conditions. The injection volume for samples and standards was 1 μL. The micrOTOF QII parameters were: temperature 225°C; drying N_2_ flow 6 L min^–1^; nebulizer N_2_ 1.5 bar, endplate offset 500 V, mass range 100–1500 Da, data were acquired at 5 scans s^–1^. Positive ion electrospray was used with a capillary voltage of 3000 V. All anthocyanins were quantified as cyanidin 3-O-glucoside equivalents (Extrasynthese, Genay, France).

#### Polyphenols

Polyphenols were separated using a Luna Omega C18 (100 × 2.1 mm, 1.6 μm) column maintained at 40°C. The mobile phase was composed of solvents: A = 0.2% formic acid and B = 100% acetonitrile at a flow rate of 400 μL/min. The solvent gradient was: initial composition 95% A, 0–0.5 min; linear gradient to 85% A, 0.5–7 min; linear gradient to 60% A, 7–13.5 min; linear gradient to 5% A, 13.5–16 min; composition held at 95% A, 16–18 min; linear gradient to 95% A, 18–18.2 min;. The injection volume and the micrOTOF QII parameters were as above. Negative ion electrospray was used with a capillary voltage of 3500 V. Polyphenolic concentrations were calculated by comparison to external calibration curves of authentic compounds (details in Supplementary Table S1).

### Gene Expression Analysis Using RNAseq

RNA was isolated from fruit tissues (100 mg) using the Spectrum^TM^ Plant Total RNA isolation kit (Sigma-Aldrich) with minor modifications. The modifications made include adding 100 μL of CTAB solution (4% PVP, 4% CTAB) to the 500 μL of lysis buffer at the lysis stage and increased volume (750 μL) of binding buffer. RNA quantity was assessed using a Nanodrop spectrophotometer (Thermo Fisher Scientific). RNA quality was assessed on the Fragment Analyser using the DNF-471 kit (Agilent).

Between 2 and 3 μg of high-quality RNA (A260/280 ratio: 1.8–2, RIN-value of ≥8) per sample was supplied to the Australian Genome Research Facility (AGRF) Ltd. Independent Illumina mRNA libraries were prepared for each biological replicate and sequenced on the Illumina NovaSeq 6000 platform in paired end mode with 150 bp read length.

Raw data was cleaned with Trimmomatic-0.36 (Bolger et al., 2014) then mapped to the *V. corymbosum* reference transcriptome (RefTrans V1) from the Genome Database For Vaccinium (GDV) using bowtie2-2.3.4.3^1^. KEGG annotation of transcripts in RefTrans V1 was performed at GDV using the KEGG/KASS server. When the tetraploid *V. corymbosum* blueberry genome was published (Colle et al., 2019), the best matching genes to the candidate RefTrans V1 sequences were identified through reciprocal ncbi-blast/2.6.0 (Johnson et al., 2008) and considered as candidate genes (see Supplementary Appendix for gene sequences). Differentially expressed genes (DEGs) were detected using DESeq2_1.22.2 (Love et al., 2014) in R version 3.5.1. Transcripts with a total read count less than 100 across samples were discarded as a data filtering step for DEG test. Transcripts with adjusted *P*-value (padj) < 0.05 and logFC larger than 2 or smaller than −2 were marked as highly differentially expressed genes (HDEGs).

Predicted amino acid sequences were validated by BLAST searches of the Arabidopsis TAIR database^2^ and the NCBI database^3^. Protein coding sequences from each gene family were then aligned with reference genes from Arabidopsis, grape and *Vaccinium* species using Geneious software version 10.9.1 to confirm homology and to identify full-length open reading frames. Gene duplicates were identified by reciprocal blast-search against Blueberry_MSU_tetraploid_proteins using our in-house sequence server database and removed from the dataset.

### Statistical Analyses and Data Visualization

All statistical analyses were conducted using R 3.5.1 version “Feather Spray” (R Core Team, 2018) at α = 0.05. Welch test (Welch, 1951) was used for univariate analysis if the data means were normally distributed according the Shapiro–Wilk test (Royston, 1982). If normality was rejected, nonparametric rank sum testing according to Kruskal–Wallis (Hollander and Douglas, 1973) and for pairwise comparisons Mann–Whitney/Wilcoxon (Bauer, 1972) were applied for sample comparisons. For multifactor analysis, analysis of variance (ANOVA) of LOG10-transformed data was used, followed by *post hoc* correction (Tukey–Kramer honest significant difference, Tukey-HSD) (Yandell, 1997). False discovery rate (FDR) adjustment for multiple comparisons were employed for all tests using the Benjamini–Hochberg method (Benjamini and Yekutieli, 2001).

Transcript abundances were normalized using Fragments Per Kilobase of transcript per Million mapped reads (FPKM-values) and genes with >1 FPKM were selected for analysis. Radial heat maps were computed using “*ggplot2*” and “*reshape*” (Wickham, 2007, 2016). The package “*RColorBrewer*” (Neuwirth, 2014) was used to select color schemes for figures. Heatmaps were constructed using the heatmap.2 function as implemented in “gplots” (Warnes et al., 2019).

Spearman rank correlations were performed using the “*Hmisc*” package (Harrell, 2019) for correlation analysis (Best and Roberts, 1975) and plots visualized with “*corrplot*” (Simko and Wei, 2017). To link chemical data with gene expression, regularized canonical correlation analysis (rCCA; Leurgans et al., 1993; González et al., 2008) was employed and Clustered Image Maps (CIM) (González et al., 2012) computed using a hierarchical clustering approach as implemented in “Biocmanager-mixomics” (Le Cao et al., 2009; Le Cao et al., 2016) by the Omics Data integration project. Relevance association networks were displayed for rCCA data according to González et al. (2012).

## Results

### Anthocyanin Composition Is Distinct Between Blueberries

**FIGURE 2:**
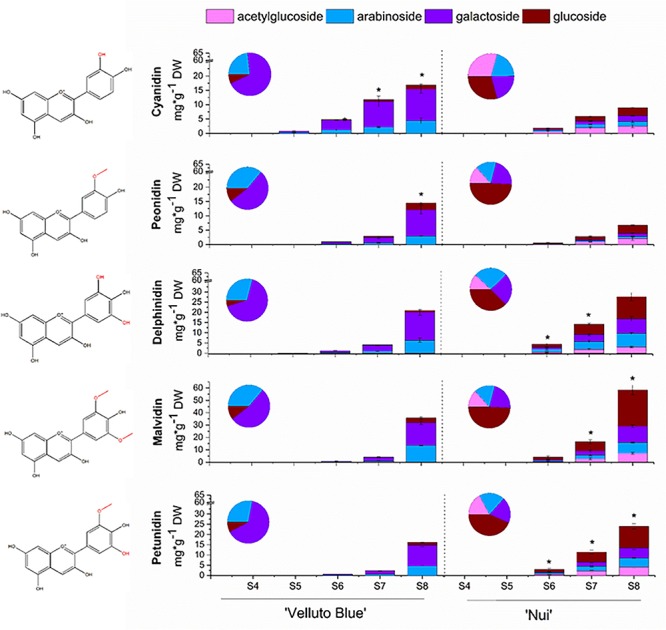
Anthocyanin profiles of *Vaccinium virgatum* ‘Velluto Blue’ (Rabbiteye) and *V. corymbosum* ‘Nui’ (Highbush) fruit skin over development (S4-green, unripe -S8 fully ripe, purple). The asterisk (*) indicates significant differences in anthocyanidin concentrations (Welch *t*-test, α = 0.05) between blueberries. Proportions of glycosides were averaged across all stages and are displayed in pie charts. Structure of each corresponding aglycone shown on left hand side.

Anthocyanin concentrations from skin and flesh of ‘Nui’ (Northern Highbush) and ‘Velluto Blue’ (Rabbiteye) blueberry were quantified throughout development. Fruit flesh was largely devoid of anthocyanins, which accumulated rapidly in skin between S6 (onset of color change) and S8 (Figure 2). In fruit skin, concentrations of total anthocyanins increased to 120 ± 12 mg/g DW in ‘Nui’ which was marginally higher at S8 (15%, *P* = 0.04) compared with ‘Velluto Blue.’ Using multifactor analysis we found that developmental stage (*F* = 171.1; *P* = 1.5 × 10^–9^) and genotype (*F* = 15.9; *P* = 0.002) both affected anthocyanin concentrations, but their interaction did not (*F* = 1.8; *P* = 0.2), confirming that production rates were comparable between genotypes.

Despite comparable amounts of total anthocyanins in skin of ripe fruit, the composition was distinctly different between the two blueberries (Figure 2): While malvidin-3-*O*-glycosides were predominant in both genotypes, concentrations measured for ‘Nui’ (58.5 ± 5.5 mg/g DW) were 1.6-fold (*P* = 0.003) higher when compared with ‘Velluto Blue.’ An increased production in 3′5′ hydroxylated/methoxylated (3′5′OH/OCH_3_) anthocyanins was observed for ‘Nui,’ whereas ‘Velluto Blue’ produced twice the amount of cyanidin- (16.9 ± 2.7 mg/g DW) and peonidin-3-*O*-glycosides (14.5 ± 1.9 mg/g DW). Thus, 3′5′OH/OCH_3_ anthocyanins were prevalent in ‘Nui’ whereas the composition of ‘Velluto Blue’ was characterized by a more balanced profile with respect to the concentrations of 3′OH/OCH_3_ and 3′5′OH/OCH_3_ anthocyanidins.

The anthocyanin profile was differentiated further by the glycone moiety. Glucosides, galactosides and arabinosides of the five common blueberry anthocyanidins were detected in both genotypes and acetylglucosides were also identified in ‘Nui’ but not in ‘Velluto Blue’ (Figure 2). In ‘Velluto Blue,’ up to 75% of anthocyanins were present as galactosides and only up to 15% as glucosides and this glycosylation pattern was largely conserved across the different groups of anthocyanidins (Supplementary Table S2). In contrast, the majority (30–40%) of anthocyanidins were linked to glucose in ‘Nui’ and the proportional glycosylation pattern between aglycones was different: cyanidin and peonidin were predominantly linked with acetylglucose, delphinidin with arabinose, and malvidin with glucose. Thus the major anthocyanin in ‘Nui’ was malvidin-3-*O*-glucoside and concentrations at S8 were eightfold higher compared with ‘Velluto Blue,’ which predominantly accumulated malvidin-3*-O*-galactoside.

### Spatiotemporal Changes of Polyphenols Are Different Between Genotypes

A further 47 polyphenols were quantified from three distinct chemical classes: HCAs, flavonols (FOL), and PAC. As these metabolites share *p*-coumaroyl-CoA as common precursor (Figure 1), their biosynthesis can either divert pathway flux away from or toward anthocyanins as pathway end products.

Across fruit development, the concentrations of measured polyphenolic metabolites were up to fourfold higher in skin compared with flesh (*P*_‘__Nui__’_ = 1.7 × 10^–5^; *P*_‘__Velluto Blue__’_ = 3.1 × 10^–6^) and did not differ significantly between the genotypes when fully mature (skin = 19.6 ± 1.3 mg/g DW, *P* = 0.34; flesh = 5.5 ± 1.5 mg/g DW; *P* = 0.07, Supplementary Figure S1), indicating that biosynthetic activity was consistently higher in fruit skin. The spatiotemporal accumulation of polyphenols, however, was different between genotypes: In ‘Nui,’ maximum concentrations were measured in skin and flesh from unripe berries (S4, Supplementary Figure S1), exceeding ‘Velluto Blue’ concentrations by twofold. While a gradual decline in polyphenols during fruit maturation was measured for ‘Nui,’ this was only observed in ‘Velluto Blue’ fruit flesh but not skin, where concentrations remained stable across time (*P* = 0.27).

**FIGURE 3:**
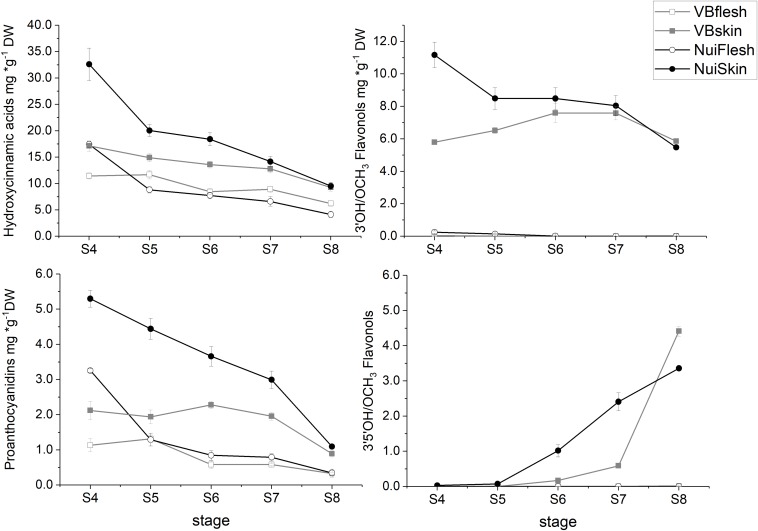
Time course of polyphenolic metabolites in blueberry tissue types during development (S4-green, unripe -S8 fully ripe, purple). Metabolites were grouped according to their chemical class and the mean (± Standard error) is visualized for *Vaccinium virgatum* ‘Velluto Blue’ (VBflesh, VBskin) and *V. corymbosum* ‘Nui’ (NuiFlesh, NuiSkin).

Overall, HCA was the main class of measured polyphenols and during maturation concentrations were consistently higher in skin than flesh for both ‘Nui’ (2-fold) and ‘Velluto Blue’ (1.5-fold), respectively. Independent of the genotype, chlorogenic acid (CGA) and its derivatives *cis*-CGA and *neo*-CGA (‘Velluto Blue’ only) were major compounds and accounted for 44.7 ± 3.3% (*P* = 0.2) and 87.2 ± 1.8% (*P* = 0.6) of measured polyphenols in skin and flesh at S8, respectively. The main difference in HCA production was the pronounced accumulation at S4 in ‘Nui’ (Figure 3), thus contributing to time but not tissue-specific differences between genotypes.

**FIGURE 4:**
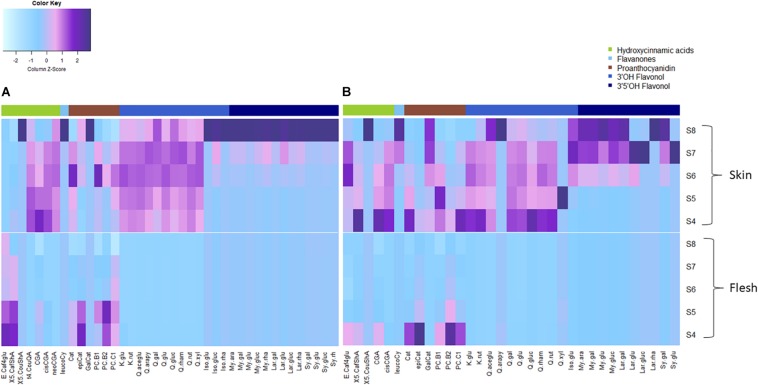
Heatmap of measured polyphenols (mean, *N* = 3) from fruit skin and flesh of **(A)** *Vaccinium virgatum* ‘Velluto Blue’ and **(B)** *V. corymbosum* ‘Nui’ berries during maturation (S4-green, unripe -S8 fully ripe, purple). Column scaling was applied for data standardization, visualizing the spatiotemporal distribution of concentrations per compound. The key to compound abbreviations is outlined in l Supplementary Table S1.

FOLs were the second largest group of non-colored polyphenols (Figure 3), with 3′OH quercetin-3-*O*-glycosides accounting for >90% of total FOL in unripe blueberry skin (S4 and S5) besides monohydroxylated kaempferol-3-*O*-glycosides as minor compounds. While quercetin-3-*O*-glycosides remained stable (6.2 ± 0.9 mg/g DW, *P* = 0.05) during maturation in ‘Velluto Blue,’ concentrations halved in ‘Nui’ from S4 over development (*P* = 1 × 10^–4^) and were comparable between genotypes at S8 (*P* = 0.96). Except for small amounts of quercetin-3-*O*-glycosides in fruit flesh from unripe berries, FOL biosynthesis appeared confined to fruit skin in both genotypes. In contrast to quercetin- and kaempferol-3-*O*-glycosides, the accumulation of 3′OCH_3_ isorhamnetin-3-*O*-glycosides and 3′5′OH/OCH_3_ FOL (laricitrin, myricetin-, syringetin-3-*O*-glycosides) coincided with anthocyanin production and increased rapidly from S6–S8 in skin of both genotypes (Figures 3, 4).

Procyanidins (ProCy) were the major group of PAC which were also higher in blueberry skin compared with flesh and reflecting 3′OH FOL accumulation patterns, particularly (Figure 3). The condensation of the ProCy monomers catechin (Cat) and epicatechin (epiCat) leads to the formation of ProCy dimers B1 (PCB1, Cat-epiCat) and B2 (PCB2, epiCat-epiCat). ProCy composition was found to be the strongest driver in genotypic profiles: similar to HCA and 3′OH FOL production, concentrations of all measured ProCy, were highest at S4 in ‘Nui’ (Supplementary Figure S2). While Cat and ProCB1 accumulation was consistently at least twofold higher and thus characteristic for ‘Nui,’ ‘Velluto Blue’ produced up to fivefold higher concentrations of epiCat (both tissues) and ProCB2 (skin only) during maturation but ProCy concentrations were generally lower when with ‘Nui’. In particular, epiCat flux was distinct and increased steadily in skin but decreased in flesh of ‘Velluto Blue’ berries (Supplementary Figure S3). Leucocyanidin (LeucoCy) is a precursor for both cyanidin and ProCy biosynthesis and accumulated in fruit skin from S6–S8 (‘Nui’) in parallel with anthocyanins when ProCy levels declined, indicating that precursor availability was unlikely limiting ProCy production. Although leucodelphinidin was not detected, its derivative the 3′5′OH PAC gallocatechin (GalCat) increased in parallel with 3′5′OH/OCH_3_ FOL in ‘Nui’ (Figure 4) but not in ‘Velluto Blue’ where GalCat was only detected at S8.

In summary, the production of measured polyphenols was higher in blueberry skin compared with flesh, which almost exclusively accumulated HCA and ProCy. In fruit skin, the production of 3′5′OH/OCH_3_ polyphenols and anthocyanins increased rapidly from S6–S8, contrasting the decline of HCA, ProCy and 3′OH FOL. The production of HCA, ProCy and 3′OH FOL was further distinguished between blueberries by pronounced accumulation in unripe berries (S4) in ‘Nui’ but not ‘Velluto Blue.’

### Gene Expression Corresponds to Metabolite Dynamics in Blueberry Tissue

**FIGURE 5:**
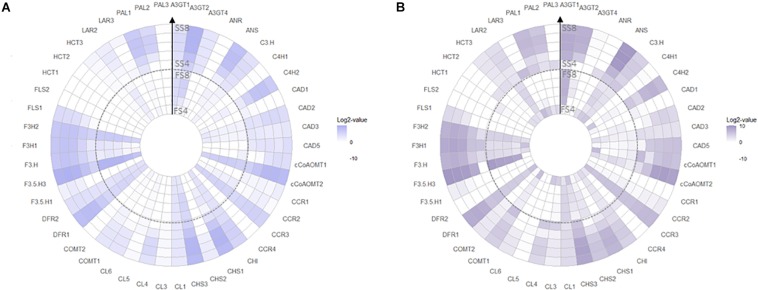
Radial heat map of Log2-transformed gene expression data for *Vaccinium virgatum* ‘Velluto Blue’ **(A)** and *V. corymbosum* ‘Nui’ **(B)** tissue types throughout development (FS4: flesh stage 4; FS8: flesh stage 8; SS4: skin stage 4; SS8: skin stage 8). Transcript abundances below 1 FPKM are white. The key to gene abbreviations is presented in Supplementary Table S3.

Transcript abundances of 20 biosynthetic genes were measured, linking phenylpropanoid-pathway as source for precursors with structural genes of the flavonoid, lignin, and HCA pathway as sinks for substrates (Figure 1). Enzymes were encoded by multiple isoforms, except for *CHI, F3*′*H, C3*′*H, ANR*, and *ANS* which appeared encoded by a single gene (Supplementary Table S3). Transcript abundance was >1 FPKM for 46 genes (Figure 5) in both blueberry genotypes which were further included in the analysis. Overall, transcriptional activity in fruit skin was fourfold higher (*P*_‘__Velluto Blue__’_ = 0.005; *P*_‘Nui’_ = 0.007) when compared with flesh and increased sharply in both genotypes from S5 to S7 in fruit skin (Supplementary Figure S4), but not in flesh. In fruit flesh, transcripts of the structural genes *CHS3, F3H2, F3*′*5*′*H3*, and *DFR1* were prevalent in addition to *CCR3* (cinnamoyl- CoA reductase 3), accounting for 61% (‘Velluto Blue’) and 54% (‘Nui’) proportional abundance across development, suggesting a role in procyanidin formation. Tissue-specific differences in terms of transcript presence/absence on the gene-family level were primarily noted for *PAL* and *FLS* which were not detected in flesh from the time of berry color change.

Reflecting increased concentrations of polyphenols in unripe ‘Nui’ berries, transcript abundances of 26 (flesh) and 12 (skin) genes were at least fivefold higher at S4 then S5, in this genotype only. This was particularly striking for *LAR3*, *ANR*, *PAL3*, *CCR4*, and Shikimate O-hydroxycinnamoyltransferase 2 (*HCT2)*, which were only expressed at this early stage (Figure 5) in both tissues. A subset of these early genes (*HCT3, PAL1, PAL2, C4*′*H2, CHS1, CHS3, F3H1, ANS*) decreased only in flesh but increased in skin during maturation.

Consistent for both genotypes, a core set of structural genes was identified using HDEG analysis at the critical time points framing berry color changes (S5–S7). Candidate genes encoding the entire phenylpropanoid pathway (*PAL1, PAL2, C4*′*H2, CL4, CL6)*, specific isoforms of the early flavonoid pathway *(CHS1, F3H1)* in combination with ANS catalyzing the final step of anthocyanidin biosynthesis (Table 1), were not only significantly more abundant in pigmented fruit skin compared with flesh but also increased sharply in fruit at S6 compared with S5. The proportional abundance of these candidates was also comparable between genotypes and between 5- and 15-fold higher in skin compared with flesh, thus marking these as main drivers of tissue-specific transcript profiles during pigmentation. Although prominent isoforms were common to both genotypes, HDEG variation was observed for decorating enzymes, catalyzing modifications of the flavonoid structure. Genotypic differences were apparent for ‘Nui’ showing particularly increased abundance of *cCoAOMT1* and c*CoAOMT2* and ‘Velluto Blue’ of *A3GT4*.

**TABLE 1:** Overview of highly differentially expressed genes (HDEG) and proportional transcript abundance in *Vaccinium corymbosum* ‘Nui’ and *V. virgatum* ‘Velluto Blue’ blueberry.

	***V. corymbosum*** ‘**Nui**’				***V. virgatum*** ‘**Velluto Blue**’			
	**HDEG Tissue**	**HDEG Time**	**% Skin S6–S7**	**% Flesh S6–S7**	**HDEG Tissue**	**HDEG Time**	**% Skin S6–S7**	**% Flesh S6–S7**
	**Skin-flesh S7**	**Skin S5–S6**	**AV ± SD**	**AV ± SD**	**Skin-flesh S7**	**Skin S5–S6**	**AV ± SD**	**AV ± SD**
	**Log2FC ± SD**	**Log2FC ± SD**			**Log2FC ± SD**	**Log2FC ± SD**		
***PAL1***	7.3 ± 0.9	2.6 ± 1.4	**1.8 ± 0.1**	0.1 ± 0.05	6.4 ± 0.1	2.1 ± 0.1	**2.6 ± 0.2**	0.14 ± 0.03
***PAL2***	7.8 ± 0.7	3.1 ± 1.5	**1.2 ± 0.1**	0.04 ± 0.02	9 ± 1.9	2.9 ± 0.3	**1.0 ± 0.1**	0.02 ± 0.01
***C4.H2***	6.2 ± 0.2	2.9 ± 1.7	**2.5 ± 0.1**	0.2 ± 0.06	5.7 ± 0.6	2.2 ± 0.1	**3.1 ± 0.3**	0.3 ± 0.08
***CL4***	7.2 ± 0.3	2.9 ± 1.5	0.4 ± 0.02	0.02 ± 0.01	6.5 ± 1.4	2.4 ± 0.3	0.5 ± 0.1	0.03 ± 0.01
***CL6***	8.8 ± 0.3	2.9 ± 0.2	0.08 ± 0.01	nd	4 ± 0.2	2.5 ± 0.1	0.2 ± 0.02	0.05 ± 0.01
CHI	2.7 ± 0.2		0.5 ± 0.05	0.5 ± 0.1			0.8 ± 0.2	0.8 ± 0.1
***CHS1***	7.5 ± 1.4	2.6 ± 1.1	**2.7 ± 0.2**	0.1 ± 0.1	6.3 ± 0.6	2.3 ± 0.1	**10.4 ± 0.8**	0.7 ± 0.3
CHS2	3.3 ± 0.4		0.8 ± 0.1	0.5 ± 0.1	5 ± 1.6		0.1 ± 0.01	0.02 ± 0.01
CHS3	6.4 ± 0.7		**7.3 ± 0.6**	0.6 ± 0.4	3.9 ± 0.6		**9.3 ± 0.4**	**3.0 ± 1.0**
***F3H1***	5.4 ± 0.5	2.2 ± 0.9	**6.1 ± 0.2**	0.9 ± 0.3	4.6 ± 0.5	2.2 ± 0.1	**3.0 ± 0.2**	0.6 ± 0.2
F3H2	3.6 ± 0.4		**3.8 ± 0.3**	**1.9 ± 0.6**			**2.8 ± 0.3**	**6.9 ± 0.9**
F3.H	4.3 ± 0.2	2.3 ± 1	**3.3 ± 0.3**	1.0 ± 0.1	4.4 ± 0.7		**2.8 ± 0.3**	0.6 ± 0.2
F3.5.H1	8.1 ± 0.1		0.1 ± 0.02	nd	8.2 ± 3.4		0.1 ± 0.02	nd
FLS1	7.6 ± 1.4	3.5 ± 1.4	0.2 ± 0.04	nd	4.9 ± 0.2		0.3 ± 0.04	0.05 ± 0.01
DFR1	3.1 ± 0.3		**4.3 ± 0.01**	**3.1 ± 0.2**	3.3 ± 0.5		**5.8 ± 1.1**	**2.8 ± 0.5**
***ANS***	6.4 ± 0.6	2.7 ± 1.5	**17.5 ± 0.5**	**1.4 ± 0.5**	6.3 ± 0.4	2.4 ± 0.2	**4.5 ± 0.1**	0.3 ± 0.1
A3GT2	5.7 ± 0.6		**3.2 ± 0.1**	0.4 ± 0.2	3.8 ± 0.3	2.7 ± 0.2	**11.9 ± 0.5**	**4.1 ± 0.9**
A3GT4			0.02 ± 0.001	0.05 ± 0.01	3.6 ± 0.4		0.2 ± 0.02	0.06 ± 0.03
COMT1	3.4 ± 0.3		0.6 ± 0.06	0.3 ± 0.03	2.6 ± 0.2		0.1 ± 0.01	0.1 ± 0.02
COMT2	3.1 ± 0.8		0.07 ± 0.01	0.05 ± 0.03			0.02 ± 0.01	0.05 ± 0.05
cCoAOMT1	5.1 ± 0.5		1.2 ± 0.2	0.2 ± 0.06			0.3 ± 0.1	0.02 ± 0.01
cCoAOMT2	6.5 ± 0.5	3.3 ± 2.1	**10.3 ± 0.7**	0.7 ± 0.3	2.8 ± 0.2		**8.1 ± 0.4**	**5.4 ± 0.8**
C3.H	2.6 ± 0.2		0.4 ± 0.07	0.4 ± 0.06	3 ± 0.2		0.7 ± 0.1	0.4 ± 0.06
CAD2	2.6 ± 0.3		0.3 ± 0.02	0.3 ± 0.1	3.1 ± 0.3		0.5 ± 0.1	0.3 ± 0.03
CAD5	4.9 ± 0.5		0.9 ± 0.04	0.2 ± 0.04	3.2 ± 0.2		0.1 ± 0.06	0.1 ± 0.02

*Only DEG with Log2-fold change (Log2FC) > 2 and Padj < 0.05 are listed. The increase in transcripts in fruit skin vs flesh at stage 7 (S7) is summarized as HDEG Tissue. HDEG Time marks genes which increased in fruit skin during time of color change (S6 compared with S5). Average values and standard deviation (SD) are given. Bold font indicates prominent genes at >1% proportional abundance and italicized genes are suggested candidates in blueberry. nd = not detected.*

**FIGURE 6:**
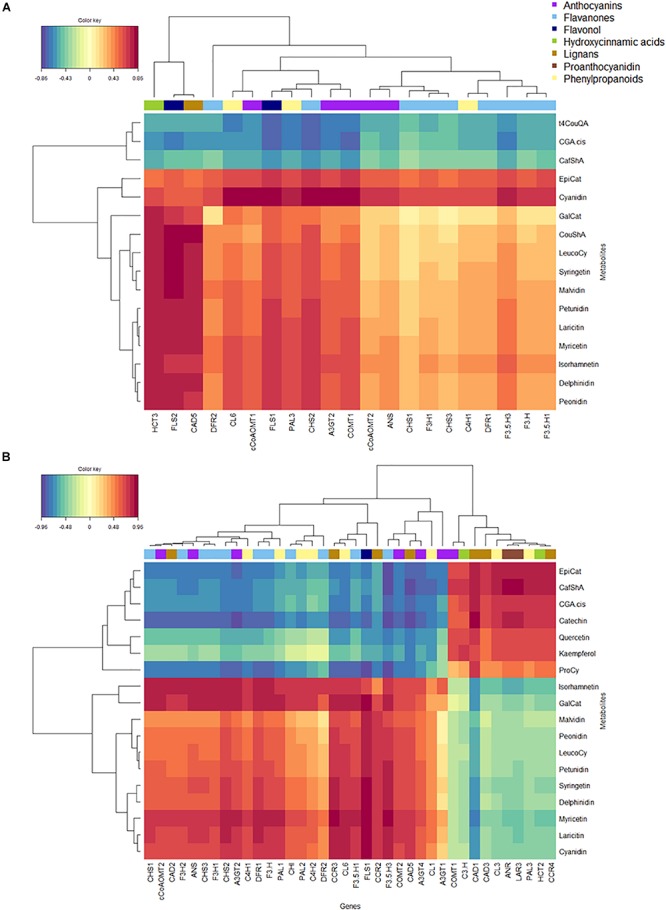
Heatmap of regularized canonical correlations between normalized expression levels of biosynthetic genes and polyphenolic metabolites in fruit skin of **(A)** *Vaccinium virgatum* ‘Velluto Blue’ and **(B)** *V. corymbosum* ‘Nui.’ The correlation threshold was set to 0.7. The dendrogram clusters the data (method “Ward”) with respect to their correlation strength. Warmer colors (red, orange) indicate positive and cooler colors (blue, green) negative correlations. Biosynthetic genes are grouped by color-bars according to their function.

We went on to co-correlate metabolites with transcripts in fruit skin using rCCA (Figure 6). Here, metabolites were grouped according to chemical class when these were correlated (Spearman’s rank correlation) over time in both blueberries. Two distinct pathway correlation clusters were identified in both genotypes (Figures 6A,B): The first was associated with HCA biosynthesis in ‘Velluto Blue’ only but co-correlated with ProCy and 3′OH FOL metabolites in addition to HCA in ‘Nui.’ This cluster was negatively correlated with the second cluster which visualized strong co-correlations of anthocyanins and 3′5′OH/OCH_3_ FOL with *FLS1*, *CHS2*, *F3*′*5*′*H3*, *A3GT2*, and *CL6* in both genotypes. In ‘Velluto Blue’ epiCat production was positively correlated with cyanidin biosynthesis (Figure 6A), suggesting simultaneous production of both compounds. In ‘Nui’ on the contrary, ProCy including Cat and epiCat were associated with HCA and not cyanidin biosynthesis, showing strong negative correlations with structural flavonoid pathway genes, except *PAL3, LAR3*, and *ANR* (Figure 6B) with which these metabolites were positively co-correlated.

### Possible Role of Transcription Factors in Gene Regulation

MYB TFs together with bHLH and WD-repeat proteins are known to regulate flavonoid biosynthesis and the effect of genotype, tissue, and their interaction on their transcript profiles (Supplementary Table S4) was analyzed. Only *bHLH1* was unaffected by these factors (*P* > 0.19), whereas five candidates (*bHLH2, MYB4, MYBA, MYBC2, MYBPA1*) were significantly higher (*P* < 6.7 × 10^–5^) in skin and *MYBR3* slightly higher (25%, *P* = 9.9 × 10^–5^) in fruit flesh. This was independent of species and factor interaction. In both genotypes transcripts of these five candidates increased steadily during maturation in skin only (Supplementary Figures S5A–C), thus resembling tissue-type dependent accumulation patterns of 3′5′OH/OCH_3_ polyphenols and anthocyanins.

Genotype had the strongest effect on expression profiles of *bHLH075* (a regulator of fruit ripening (Fasoli et al., 2018)), *WDR1*, *MYBPA2a*, and *MYBPA2b*. *MYBPA2a* and *MYBPA2b* are likely associated with PAC biosynthesis (Terrier et al., 2009) and transcript abundance of both genes were below 1 FPKM in ‘Velluto Blue,’ whereas *bHLH075* and *WDR1* were each twofold higher (*P* < 5 × 10^–4^) compared with ‘Nui’ (Supplementary Figures S5A–C). *MYBPA2a* and *MYBPA2b* were co-expressed with *LAR3*, and *ANR* in ‘Nui’ which coincided with increased ProCy concentrations at S4.

**FIGURE 7:**
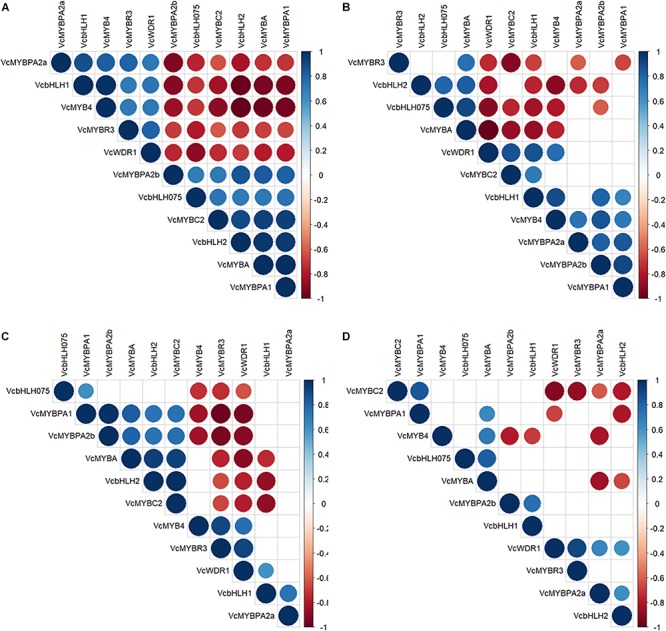
Spearman-rank correlation coefficients of transcription factor gene expressions in skin **(A,C)** and flesh **(B,D)** of *Vaccinium corymbosum* ‘Nui’ **(A,B)** and *V. virgatum* ‘Velluto Blue’ **(C,D)** blueberry. Positive correlations are displayed in blue, negative ones in red and non-significant relationships (α = 0.05) are left blank.

To identify interactions of these TFs, we correlated gene transcription during fruit maturation (Figure 7). In fruit skin, two cohorts were identified of which *bHLH2*, *MYBA*, *MYBC2*, and *MYBPA1* (cohort 1) were co-expressed in skin of both genotypes and negatively correlated with, *MYB4*, *MYBR3*, and *WDR1* (cohort 2). Of these, cohort 1 was expressed in parallel with anthocyanin production during ripening, suggesting a possible function as activators. In fruit flesh, *MYBA*, *MYBPA1*, and *MYBC2* were not co-expressed and fewer significant correlations were apparent overall.

**FIGURE 8:**
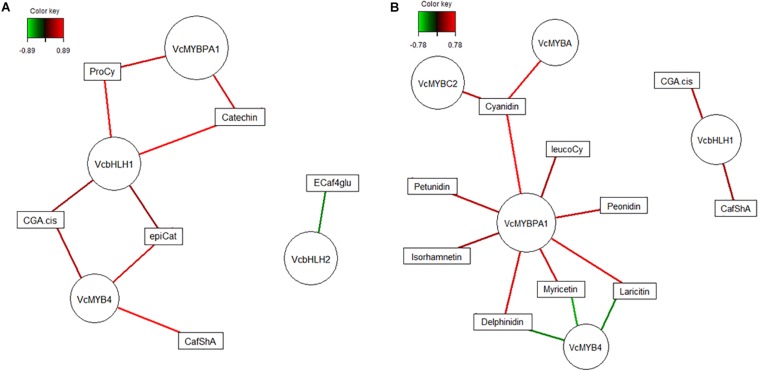
Relevance network graph depicting correlations derived from regularized canonical correlation analysis between polyphenolic metabolites and genotype-independent transcription factors for blueberries (combined data, *Vaccinium virgatum* and *V. corymbosum*) flesh **(A)** and skin **(B)**. Nodes (circles) represent variables and are sized according to number of connections. Lines are colored according to correlation strength with augmented intensity indicating higher strength, green negative and red positive values, correlation threshold were set 0.8 for **(A)** and 0.7 for **(B)**.

In order to propose a general model for the potential function of TF interactions on polyphenol biosynthesis in blueberry, we further focused on tissue-type dependent TFs only and co-correlated their transcripts with metabolites across both genotypes. The relevance networks (Figure 8) visualize strong correlations only (cut-off >0.7) and for fruit flesh (Figure 8A) a positive relationship between *MYBPA1*, *bHLH1*, and *MYB4* and metabolites from HCA and PA pathways is suggested. Although *MYB4* is classified as a repressor of gene transcription (Jin et al., 2000), it is unlikely that this MYB acts as a repressor of HCA and PAC pathway genes directly. These same TFs were also strongly correlated to metabolites in fruit skin (Figure 8B) where associations with more defined sections of the pathway were apparent. While *bHLH1* was associated with HCA primarily, *MYBPA1*, together with *MYBA* and *MYBC2*, were positively correlated with the accumulation of anthocyanins and 3′5′ substituted polyphenols. In contrast, *MYB4* was negatively correlated suggesting a possible repression of flavonoids in particular.

## Discussion

### Precursor Limitation as Likely Cause for Flavonoid Deficiency in Fruit Flesh

We studied flavonoid biosynthesis during fruit maturation by correlating metabolite concentrations with gene transcript abundance in ‘Velluto Blue’ (Rabbiteye) and ‘Nui’ (Northern Highbush) blueberry. The accumulation of anthocyanins, gallocatechin and flavonols was confined to fruit skin. Procyanidins and HCAs were produced in fruit flesh, although at lower concentrations, and their concentrations declined in both tissues during maturation. In fruit skin, the production of anthocyanins and trihydroxylated flavonoids was strongly co-correlated with structural genes leading to their biosynthesis and negatively correlated with HCAs, thus indicating likely competition for substrates between pathways. This confirms conclusions from previous studies in Highbush and Lowbush blueberries (Gibson et al., 2013; Li et al., 2019b) suggesting that precursors might be increasingly used for flavonoid instead of HCA biosynthesis when fruit ripens. In both tissues, however, HCAs declined in parallel with procyanidin biosynthesis, thus indicating that precursors were shared and pathways might be co-regulated. While a diversion of substrates away from procyanidin biosynthesis toward cyanidins might likely explain the temporal shift observed in fruit skin at the onset of pigmentation, low gene transcription in fruit flesh of the phenylpropanoid pathway in general and *PAL* genes in particular, indicated limited supply of precursors as primary cause for reduced flavonoid production rather than substrate competition. During ripening, at least one isoform of structural flavonoid genes was prevalent in fruit flesh (with the exception of *FLS*) and the integrity of the pathway was therefore not interrupted at any particular step. Instead limited transcription of multiple pathway genes were identified as bottlenecks: In addition to isoforms of the phenylpropanoid pathway (*PAL1-2*, *C4*′*H2*, *4CL4/6*), structural genes catalyzing formation of flavanone scaffolds (*F3H1*, *CHS1*) in combination with *ANS* were likely restricting anthocyanin formation in fruit flesh. This is in line with previous studies on anthocyanin-deficient raspberry (Rafique et al., 2016) and bilberry (Zorenc et al., 2017). In contrast to our findings, DFR-activity was also reduced in albino bilberry and a concomitant increase in flavonols reported. Substrate competition between DFR and FLS has previously suggested to regulate anthocyanin formation (Davies et al., 2003) but this was unlikely causing the observed lack of pigmentation in our study as blueberry flesh was largely devoid of flavonols and *FLS* gene expression.

### Anthocyanin Biosynthesis Was Likely Regulated by Coexpression of Transcriptional Activators MYBA, MYBPA1, and the Repressor MYBC2

Trihydroxylated flavonoids accumulated in parallel with anthocyanins, which was concomitant with expression patterns of transcriptional activators *MYBA* and *MYBPA1* in addition to *bHLH2* and the repressor *MYBC2*. The co-expression of the repressor *MYBC2* with the activators *MYBA* and *MYBPA1* fit proposed models, where the activators regulate repressors to provide feedback repression and fine control of anthocyanin production (Albert et al., 2014). Under these models, the expression of the R2R3-MYB repressor (*MYBC2*) and R3-MYB repressor (*MYBR3*) would be expressed most highly in tissues that accumulate key activators of anthocyanin or proanthocyanidin biosynthesis. Genotype-specific transcriptional regulation was most apparent for proanthocyanidin biosynthesis which was likely regulated by *MYBPA2a* in Northern Highbush fruit while no co-correlations with any of the *MYBPA* TSs were identified in Rabbiteye.

Interestingly, the expression of *MYBR3* was not correlated with *MYBA*, *MYBPA1*, or *MYBPA2* transcription as expected, but was more highly expressed in the flesh of berries than skin. In albino bilberry (Zorenc et al., 2017) *MYBR3* was also significantly increased while *MYBC2* and *MYBPA1* were lower compared with the pigmented phenotype. It is, however, unlikely that *MYBR3* is responsible for the lack of anthocyanins in the flesh, because its mode of action is to titrate bHLH proteins (Albert et al., 2014) which would also affect proanthocyanidin biosynthesis. Proanthocyanidins were reduced in fruit flesh compared with skin but not abolished, indicating that *MYBR3* might indeed suppress flavonoid production to some degree but not entirely.

### Flavonoid Biosynthesis Is Distinct Between Northern Highbush and Rabbiteye Blueberry in the Early Stages of Fruit Development

In line with our observations, quercetin-3-*O*-glycosides have previously been described as the most abundant flavonols in Northern Highbush blueberry (Häkkinen et al., 1999) and we show tissue-dependent accumulation in fruit skin. Maximum concentrations have been reported at the early stages of berry development when amounts of HCA and PAC were also high (Jaakola et al., 2002; Zifkin et al., 2012). We confirm these trends for the Northern Highbush but not for the Rabbiteye cultivar, where procyanidins and dihydroxylated flavonols were relatively consistent over time and not pronounced at S4. These diverging metabolic profiles correlated well with transcript abundances of structural genes, which remained stable in green Rabbiteye berries but were down-regulated in Northern Highbush after S4. In skin and flesh of Northern Highbush, increased transcription of the phenylpropanoid pathway genes (*PAL, C4′H, 4CL*) was concomitant with *HCT, C3*′*H*, and *cCoAOMT* at S4, which likely corresponded with the observed early peak in HCA production. In skin, the production of quercetin-3-*O*-glycosides coincided with early expression of *F3*′*H*, *CHI, CHS*, and *F3H* isoforms but not *FLS*. *DFR* and *ANS* transcripts were also detected at S4 in the absence of anthocyanins, suggesting that *ANS* expression might primarily affect procyanidin biosynthesis as procyanidin accumulation was strongly correlated with the early expression of *LAR3*, and *ANR* in Northern Highbush. Earlier findings in Northern Highbush by Zifkin et al. (2012), postulated that flavan-3-ol synthesis was likely restricted to early stages of fruit development, providing sufficient pools of monomers to drive procyanidin production further into S5 and S6. For Rabbiteye, however, neither procyanidin biosynthesis nor expression of *LAR or ANR* was pronounced early in development. Our data indicate heterogeneity in the expression profile of the *LAR* gene family, with the presence of *LAR2* transcripts in the skin and flesh of both blueberry species well into the later stages of development, suggesting that procyanidin biosynthesis likely continued past berry color change. In fruit skin, the metabolic flux of procyanidins resembled patterns observed for quercetin-3-*O*-glycosides, indicating that precursors were shared between both groups of flavonoids. The prodelphinidin gallocatechin, on the contrary, accumulated in parallel with trihydroxylated flavonols (myricetin- and laricitrin-, syringetin-3-O-glycosides), which was consistent for both species. Differences in proanthocyanidin biosynthesis were not previously identified in blueberry and our findings suggest prodelphinidin biosynthesis was likely dependent on availability of structurally different substrates in addition to *LAR2* gene transcription.

### Genotype-Characteristic Anthocyanin Profiles Were Likely Determined by Flavonoid Flux and Decorating Enzymes

We observed genotypic differences in anthocyanin production, which affected their onset and composition, but not their total amounts or distributions between tissue types. In Northern Highbush, anthocyanins accumulated simultaneously in fruit skin from S6 onward whereas in Rabbiteye the onset of cyanidin production occurred earlier in fruit development (S5), preceding other anthocyanins. Small amounts of anthocyanidin-3-*O*-glycoside have been previously measured in green Northern Highbush berries by Zifkin et al. (2012) and Li et al. (2019b). The use of different experimental techniques, however, challenges conclusions to whether these diverging findings might be based on genetic variation. Since standardized conditions were applied in our comparative study, we suggest that leucocyanidin might have been more efficiently used as precursor for cyanidin instead of procyanidin biosynthesis in Rabbiteye, reflecting low abundance of *LAR3* and *ANR* transcripts. In ripe fruit, the composition of anthocyanins was distinct between the blueberries with Rabbiteye producing significantly higher amounts of 3′ and Northern Highbush of 3′5′ substituted anthocyanins. Although only one genotype per species was analyzed in this study, Lohachoompol et al. (2008) reported similar trends for anthocyanin profiles from ripe fruit based on three Northern Highbush and Rabbiteye genotypes each, indicating that the observed differences in anthocyanin composition are likely to be a species-specific feature.

Fruit-specific anthocyanin profiles are likely genetically predisposed, but plasticity in pigment composition has also been emphasized in response to environmental factors such as light and temperature (Karppinen et al., 2016; Spinardi et al., 2019). As Rabbiteye fruit usually mature toward the later summer months and – as in our case – harvest dates can be months’ apart, seasonal effects on anthocyanin accumulation cannot be excluded. Our knowledge of these effects, however, does not sufficiently explain the observed accumulation of cyanidin-based anthocyanins in Rabbiteye compared with Northern Highbush, because delphinidin- and malvidin-based anthocyanins were found to predominantly increase with light wavelength (Zoratti et al., 2014; Zoratti et al., 2015; Spinardi et al., 2019), at least in Northern Highbush and bilberry fruit.

The production of delphinidin-based anthocyanins was shown to depend on *F3*′*5*′*H* transgene expression in fruits naturally devoid of trihydroxylated anthocyanins (Brendolise et al., 2017) and in blueberry, accumulation of trihydroxylated anthocyanins was suggested to depend on *F3*′*5*′*H* gene expression (Zifkin et al., 2012). Using whole fruit samples, a late induction (S7/S8) in *F3*′*5*′*H* gene expression was found by Zifkin et al. (2012) and an increase in F3′5′H proteins was reported during development of Northern Highbush blueberry in a recent study (Li et al., 2019b). Thus making *F3*′*5*′*H* a strong candidate for regulating the accumulation of trihydroxylated flavonoids in ripe fruit.

We identified two *F3*′*5*′*H* isoforms that co-correlated with trihydroxylated flavonoids in blueberry skin. While *F3*′*5*′*H1* transcripts accumulated predominantly in skin, the major isoform *F3*′*5*′*H3* was also highly expressed in fruit flesh, reflecting both increased production of dihydroxylated flavonoids in unripe Northern Highbush as well as accumulation of trihydroxylated flavonoids during ripening. While our data confirm that increased *F3*′*5*′*H* gene expression was concomitant with increased flavonoid production, we conclude that tissue-specific biosynthesis was unlikely regulated by *F3*′*5*′*H* gene expression alone. The formation of trihydroxylated compounds is described as two-step reaction, and F3′5′H is therefore considered to exhibit both F3′H and F5′H activity. Since dihydroxylated flavonoids were predominant in unripe fruit and fruit flesh, even in the absence of *F3*′*H* gene expression, we postulate that *F3*′*5*′*H3* was primarily involved with generating dihydroxylated compounds due to high substrate turnover and formation of trihydroxylated compounds occurred later when excess substrate was available.

While *F3*′*5*′*H* expression is undoubtedly necessary for the production of delphinidin-based anthocyanins, *F3*′*5*′*H* as well as *F3*′*H* transcript abundances were within the same range for Northern Highbush and Rabbiteye fruit and therefore unlikely driving observed differences in anthocyanidin composition. We found structural genes linking phenylpropanoid and flavonoid pathways co-expressed and therefore conclude that anthocyanin composition in fruit skin was likely modulated by genotype-specific pathway flux, resembling the concerted action and interaction of each element of the pathway rather than regulated by individual genes. For example, ANS produces precursors for both anthocyanins and PAC, and gene expression was fourfold higher in Northern Highbush, while the actual anthocyanin concentrations were comparable between species. Since procyanidins were also highest in this species, especially during berry development, we suggest that this accumulation might have been at the expense of leucocyanidin, leading to lower cyanidin accumulation compared with Rabbiteye. That substrate competition between LAR and ANS can affect cyanidin concentrations has recently been shown in crabapple (Li et al., 2019a) but how this relationship might affect composition between di-and trihydroxylated anthocyanins has not been explored to date.

Anthocyanins are also structurally modified by methyltransferases, which were indicative for Northern Highbush and increased concentrations of malvidin- and petunidin-3-*O-*glycosides in this species might have been related to 3- and 5-fold higher expression of COMTs and cCoAOMTs, respectively. In addition to methyl transferases, the expression of UDP-glucose: flavonoid-3-*O*-glycosyltransferases was genotype-characteristic and likely to affect characteristic pigment compositions. To our knowledge, specific glycosylation patterns of blueberry anthocyanins have not been focussed on in previous literature, but have been suggested as markers for the authenticity of bilberry products (Primetta et al., 2013). We have shown that Northern Highbush produced 30% more anthocyanidin-3-*O*-glucosides than Rabbiteye, which produced pigments predominantly linked to galactose. These results are of interest as the bioavailability of anthocyanins was shown to be influenced by the nature of the aglycone in combination with its glycoside. Firstly, systemically, after absorption into the bloodstream, arabinosides were generally less bioavailable than glucosides (McGhie et al., 2003). In addition, methoxylation also improved bioabsorption, and malvidin-3-*O*-glucoside was most efficiently absorbed. Secondly, *in vitro* and gut studies have suggest that glucosides were most efficiently metabolized by the intestinal microflora (Aura et al., 2005).

## Conclusion

In summary, we conclude that anthocyanin biosynthesis is likely modulated by flavonoid flux within the pathway and fine-tuned by decorating enzymes with respect to structural composition. The control of the pathway, however, involves multiple pathway steps in association with multiple TFs in an interdependent manner.

## Data Availability Statement

The full raw sequence reads used for comparative RNAseq-based transcriptomics analyses have been made available on NCBI.

PRJNA591951: *Vaccinium virgatum* ‘Velluto Blue’ (TaxID: 1493660) https://www.ncbi.nlm.nih.gov/bioproject/PRJNA591951.

PRJNA591663: *Vaccinium corymbosum* ‘Nui’ (TaxID: 69266) https://www.ncbi.nlm.nih.gov/bioproject/PRJNA591663.

The raw data supporting the conclusions of this article will be made available by the authors, without undue reservation, to any qualified researcher.

## Author Contributions

RE, AD, and NA conceived the experimental design and supervised all aspects of the study. CG performed the experiments, analyzed the data, and wrote the manuscript. CD was leading Bioinformatics of RNA sequencing. DL contributed to the data analysis and manuscript writing with respect to transcription factors. LJ provided academic advice on all sections of the manuscript. TM performed the LC-MS experiments, metabolite identification, and quantification. EG, JT, and BP provided support in sample collection, sample processing, and preparation of the manuscript.

## Conflict of Interest

The authors declare that the research was conducted in the absence of any commercial or financial relationships that could be construed as a potential conflict of interest.

## Footnotes

1 http://bowtie-bio.sourceforge.net/bowtie2/index.shtml

2 https://www.arabidopsis.org/

3 https://blast.ncbi.nlm.nih.gov/Blast.cgi

## Supplementary Material

FIGURE S1 Concentrations of total measured polyphenols in blueberry fruit tissues during.

FIGURE S2 Differences in procyanidin composition between blueberries.

FIGURE S3 Epicatechin accumulation in blueberry tissues during fruit maturation.

FIGURE S4 Combined transcript counts of biosynthetic genes and transcription factors in blueberry tissues during fruit maturation.

FIGURE S5A Gene expression profiles of *bHLH*, *MYB* and *WDR1* transcription factors in blueberry tissue types during fruit maturation.

TABLE S1 Summary of compound identification from blueberry tissues using LC-MS.

TABLE S2 Glycosylation patterns of anthocyanidins from blueberry (*Vaccinium*) skin.

TABLE S3 Overview of gene mapping results and functional annotation.

TABLE S4 Summary of factorial ANOVA testing the effect of blueberry genotype, tissue, and interaction on gene expression of candidate transcription factors.

APPENDIX Coding sequences of genes listed in Supplementary Table S3.
