# Supplementary material for: Spatiotemporal Modulation of Flavonoid Metabolism in Blueberries
Source: Front Plant Sci. 2020 May 13;11:545. doi: 10.3389/fpls.2020.00545 (PMC7237752; doi:10.3389/fpls.2020.00545)

Supplementary Table S1:
Summary of compound identification from blueberry tissues using LC-MS. RT: retention time; CAS: Chemical abstract service registry number; Equivalence: quantified in equivalence of the listed standard compound; ID confidence: Degree of confidence in accuracy identification.

Compound	Abbreviation	RT(min)	Formula	CAS	Exact Mass	Equivalence	ID confidence
(E)-caffeoyl 4-glucoside	E.Caf4glu	4.68	C15H18O9		342.0951	chlorogenic acid	2
4-coumaroylshikimic acid	X5.CouShA	0	C16H16O7	196496-50-1	320.0896	chlorogenic acid	3
5-caffeoylshikimic acid	X5.CafShA	7.37	C16H16O8	73263-62-4	336.0845	chlorogenic acid	2
caffeic acid	CafA	5.42	C9H8O4	331-39-5	180.0423	chlorogenic acid	1
catechin	Cat	4.68	C15H14O6	154-24-4	290.079	catechin	1
chlorogenic acid	CGA	5.17	C16H18O9	327-97-9	354.0951	chlorogenic acid	1
cis-chlorogenic acid	cisCGA	6.21	C16H18O9	15016-60-1	354.0951	chlorogenic acid	2
epicatechin	epiCat	6.42	C15H14O6	490-49-0	290.079	epicatechin	1
ferulic acid	FA	8.85	C10H10O4	1135-24-6	194.0579	chlorogenic acid	1
gallic acid	GA	1.26	C7H6O5	149-91-7	170.0215	epicatechin	1
gallocatechin	GalCat	2.38	C15H14O7	970-73-0	306.0739	epicatechin	3
isorhamnetin 3-glucoside	Iso.glu	10.65	C22H22O12	5041-82-7	478.1111	quercetin 3-galactoside	2
isorhamnetin 3-glucuronide	Iso.gluc	10.86	C22H20O13	36687-76-0	492.0904	quercetin 3-galactoside	2
isorhamnetin 3-rhamnoside	Iso.rha	11.63	C22H22O11	67068-82-0	462.116	quercetin 3-galactoside	2
kaempferol 3-glucoside	K.glu	10.6	C21H20O11	480-10-4	448.1006	quercetin 3-galactoside	2
kaempferol 3-rutinoside	K.rut	10.3	C27H30O15	17650-84-9	594.1585	quercetin 3-galactoside	1
laricitrin 3-galactoside	Lar.gal	9.91	C22H22O13	93219-26-2	494.106	quercetin 3-galactoside	2
laricitrin 3-glucoside	Lar.glu	9.99	C22H22O13	39986-90-8	494.106	quercetin 3-galactoside	2
laricitrin 3-glucuronide	Lar.gluc	9.99	C22H20O14	1026666-46-5	508.0853	quercetin 3-galactoside	2
laricitrin 3-rhamnoside	Lar.rha	10.81	C22H22O12		478.1111	quercetin 3-galactoside	2
leucocyanidin	leucoCy	4.3	C15H14O7	480-17-1	306.074	epicatechin	1
leucodelphinidin	leucoDp	0	C15H14O8	491-52-1	322.0689	epicatechin	3
myricetin 3-arabinoside	My.ara	9.45	C20H18O12	26856-98-4	450.0798	quercetin 3-galactoside	2
myricetin 3-galactoside	My.gal	8.57	C21H20O13	15648-86-9	480.0904	quercetin 3-galactoside	2
myricetin 3-glucoside	My.glu	8.73	C21H20O13	19833-12-6	480.0904	quercetin 3-galactoside	2
myricetin 3-glucuronide	My.gluc	8.64	C21H18O14	77363-65-6	494.0697	quercetin 3-galactoside	2
myricetin 3-rhamnoside	My.rha	9.57	C21H20O12	17912-87-7	464.0955	quercetin 3-galactoside	2
naringenin	Nar	13.33	C15H12O5	480-41-1	272.0685	quercetin 3-galactoside	1
neochlorogenic acid	neoCGA	3.14	C16H18O9	202650-88-2	354.0951	chlorogenic acid	2
p-coumaric acid	pCouA	7.67	C9H8O3	7400-08-0	164.0473	quercetin 3-galactoside	1
procyanidin B1	PC.B1	4.28	C30H26O12	20315-25-7	578.1424	procyanidin B2	1
procyanidin B2	PC.B2	6.03	C30H26O12	29106-49-8	578.1424	procyanidin B2	1
procyanidin Cx	PC.Cx	5.37	C45H38O18	37064-30-5	866.2058	procyanidin B2	2
quercetin	Q	12.44	C15H10O7	117-39-5	302.0427	quercetin 3-galactoside	1
quercetin 3-acetyl-glucoside	Q.aceglu	10.28	C23H22O13	54542-51-7	506.106	quercetin 3-galactoside	2
quercetin 3-arabinopyranoside	Q.arapy	10.47	C20H18O11	22255-13-6	434.0849	quercetin 3-galactoside	2
quercetin 3-galactoside	Q.gal	9.71	C21H20O12	482-36-0	464.0955	quercetin 3-galactoside	1
quercetin 3-glucoside	Q.glu	9.86	C21H20O12	482-35-9	464.0955	quercetin 3-galactoside	2
quercetin 3-glucuronide	Q.gluc	9.79	C21H18O13	22688-79-5	478.0747	quercetin 3-galactoside	2
quercetin 3-rhamnoside	Q.rham	10.66	C21H20O11	522-12-3	448.1006	quercetin 3-galactoside	1
quercetin 3-rutinoside	Q.rut	9.63	C27H30O16	153-18-4	610.1534	quercetin 3-galactoside	1
quercetin 3-xyloside	Q.xyl	10.19	C20H18O11	549-32-6	434.0849	quercetin 3-galactoside	2
syringetin 3-galactoside	Sy.gal	10.77	C23H24O13	55025-56-4	508.1217	quercetin 3-galactoside	2
syringetin 3-glucoside	Sy.glu	10.82	C23H24O13	40039-49-4	508.1217	quercetin 3-galactoside	2
syringetin 3-glucuronide	Sy.gluc	10.91	C23H22O14	1094607-09-6	522.101	quercetin 3-galactoside	2
syringetin 3-rhamnoside	Sy.rh	11.7	C23H24O12	93126-00-2	492.1268	quercetin 3-galactoside	2
trans-4-p-coumaroyl quinic acid	t4.CouQA	6.6	C16H18O8	1108200-72-1	338.1002	trans-4-p-coumaroyl quinic acid	1
cyanidin 3-(6-acetyl)glucoside	Cy.acglu	12.63	C23H23O12	784107-03-5	491.1184	cyanidin 3-glucoside	2
cyanidin 3-arabinoside	Cy.ara	7.96	C20H19O10	27214-72-8	419.0973	cyanidin 3-glucoside	2
cyanidin 3-galactoside	Cy.gal	6.62	C21H21O11	142506-26-1	449.1078	cyanidin 3-glucoside	1
cyanidin 3-glucoside	Cy.glu	7.31	C21H21O11	7084-24-4	449.1078	cyanidin 3-glucoside	1
delphinidin 3-(6-acetyl)glucoside	Dp.acglu	11.36	C23H23O13	753416-12-5	507.1133	cyanidin 3-glucoside	2
delphinidin 3-arabinoside	Dp.ara	6.8	C20H19O11	324533-67-7	435.0922	cyanidin 3-glucoside	2
delphinidin 3-galactoside	Dp.gal	5.5	C21H21O12	28500-00-7	465.1028	cyanidin 3-glucoside	2
delphinidin 3-glucoside	Dp.glu	6.08	C21H21O12	50986-17-9	465.1028	cyanidin 3-glucoside	2
malvidin 3-(6-acetyl)glucoside	Mv.acglu	14.37	C25H27O13	732279-31-1	535.1446	cyanidin 3-glucoside	2
malvidin 3-arabinoside	Mv.ara	11.24	C22H23O11	863107-21-5	463.1235	cyanidin 3-glucoside	2
malvidin 3-galactoside	Mv.gal	9.88	C23H25O12	104880 34-4	493.1341	cyanidin 3-glucoside	2
malvidin 3-glucoside	Mv.glu	10.49	C23H25O12	7228-78-6	493.1341	cyanidin 3-glucoside	1
peonidin 3-(6-acetyl)glucoside	Pn.acglu	14.05	C24H25O12	751447-22-0	505.1341	cyanidin 3-glucoside	2
peonidin 3-arabinoside	Pn.ara	10.3	C21H21O10	27214-74-0	433.1129	cyanidin 3-glucoside	2
peonidin 3-galactoside	Pn.gal	8.93	C22H23O11	260256-26-6	463.1235	cyanidin 3-glucoside	2
peonidin 3-glucoside	Pn.glu	9.65	C22H23O11	68795 37-9	463.1235	cyanidin 3-glucoside	2
petunidin 3-(6-acetyl)glucoside	Pt.acglu	13.91	C24H25O13	774148-75-3	521.129	cyanidin 3-glucoside	2
petunidin 3-arabinoside	Pt.ara	9.09	C21H20O11	749848 37-1	449.1078	cyanidin 3-glucoside	2
petunidin 3-galactoside	Pt.gal	7.78	C22H23O12	260256-23 3	479.119	cyanidin 3-glucoside	2
petunidin 3-glucoside	Pt.glu	8.37	C22H23O12	6988-81-4	479.119	cyanidin 3-glucoside	2

ID Confidence
1 High-tested against authentic standard
2 Good- exact mass
3 Reasonable- ambiguity possible

Table S2: Glycosylation patterns of anthocyanidins from blueberry (*Vaccinium*) skin.

Proportions were averaged over development if time had no significant effect (Kruskal-Wallis test, α=0.05) on concentrations. If levels differed over development the stage producing maximum and minimum amounts is listed.

	V. virgatum 'Velluto Blue'<br>Average % ± Std			*V. corymbosum* 'Nui'<br>Average % ± Std (%)			
	**Arabinoside**	**Galactoside**	**Glucoside**	**AcetylGlucoside**	**Arabinoside**	**Galactoside**	**Glucoside**
**Cyanidin**	S4: 39.8 ± 1.3<br>S7: 18.6 ± 1.9	S4: 60.2 ± 1.3<br>S7: 75.9 ± 2.5	6.0 ± 1.7	28.9 ± 2.9	22.8 ± 5.3	19.4 ± 3 .3	29 ± 1 .9
**Peonidin**	20.9 ± 0.1	63.4 ± 0.5	15.7 ± 0.8	31.2 ± 1.6	12.7 ± 2.5	15.4 ± 0.4	40.8 ± 3.7
**Delphinidin**	S5: 41.2 ± 9.3<br>S8: 30.2 ± 2.9	66.2 ± 5.7	4.2 ± 0.8	11.8 ± 1.4	S6: 33.9 ± 1.8<br>S8: 24.4 ± 2	S6: 21.2± 0.9<br>S8:2 5.5 ± 1	S6: 33.8 ± 1.2<br>S8: 39 ± 1.7
**Malvidin**	S6: 26.6 ± 2.7<br>S8: 38 ± 2.3	62.9 ± 7.3	8.6 ± 1.9	S6: 11.3 ± 0.2<br>S7: 16.8 ± 2.1	16.7 ± 1.6	20.4 ± 1.7	39.4 ± 3.65
**Petunidin**	29.5 ± 4.8	65.6 ± 2.5	S6:4.8 ± 0.9<br>S8:7.9 ± 0.6	16.7 ± 1.7	22.2 ± 4.7	18 ± 2.8	43.1 ± 0.9

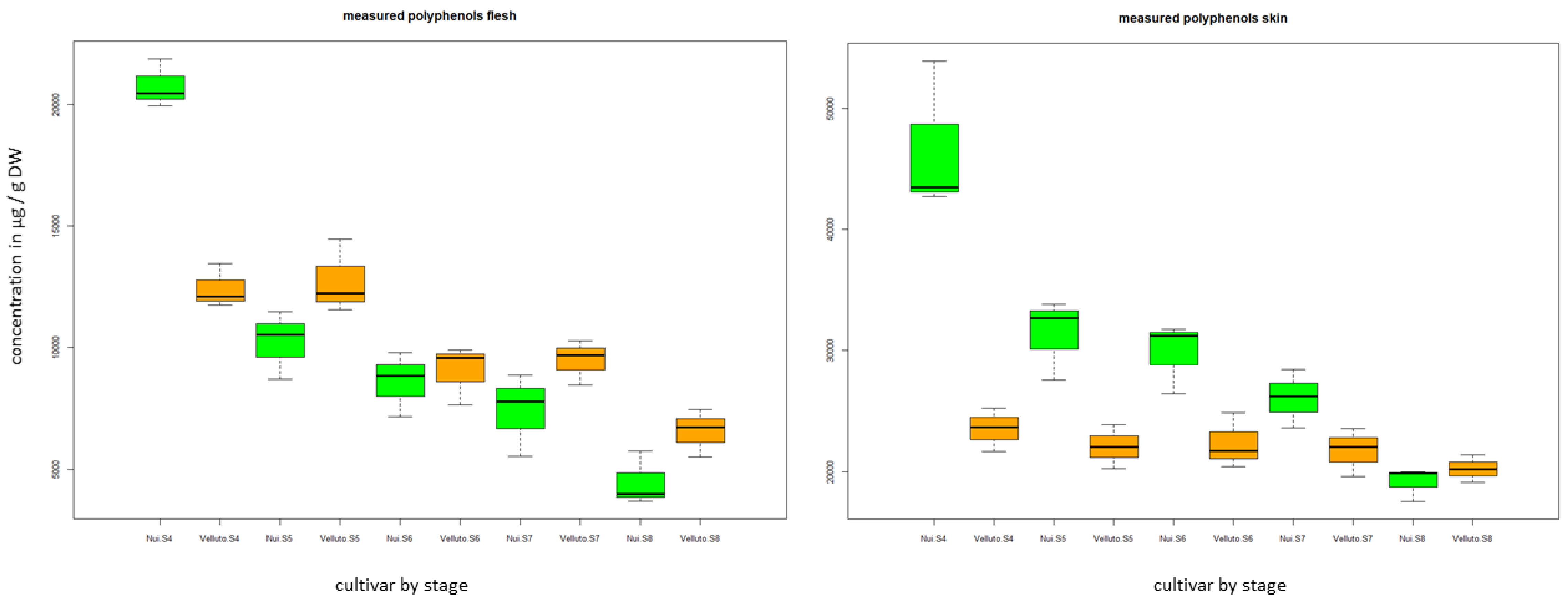

Figure S1:
Concentrations of total measured polyphenols in blueberry (*Vaccinium virgatum* ' Velluto Blue' (orange); *V. corymbosum* 'Nui' (green)) fruit tissue types (left: flesh; right: skin) during maturation (S4-S8).

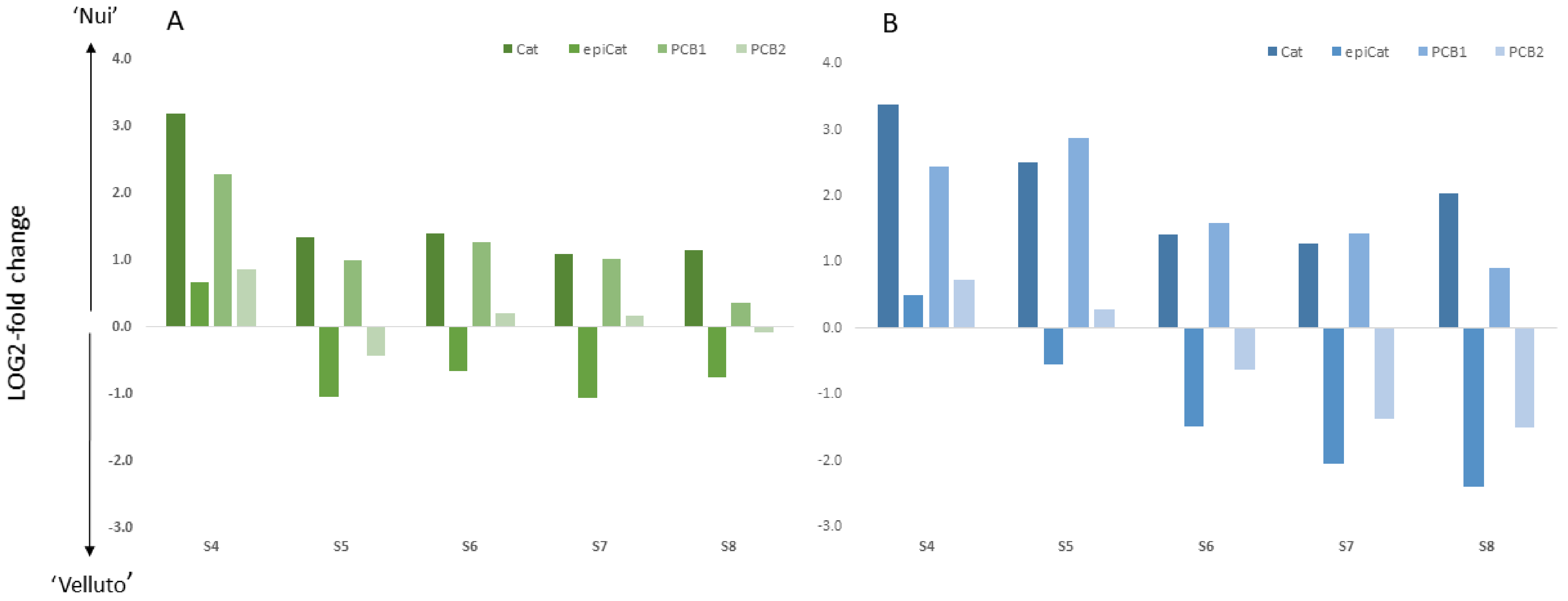

Figure S2:
Differences in procyanidin composition between blueberries (*Vaccinium virgatum* 'Velluto Blue' and *V. corymbosum* 'Nui'). Data are scaled on 'Nui' and deliver positive factors (expressed in LOG2-fold change) when concentrations are higher and negative values when these are lower compared to 'Velluto Blue'. (A) fruit flesh; (B) skin. Developmental stages: S4 (green/unripe)- S8 (purple/ ripe).

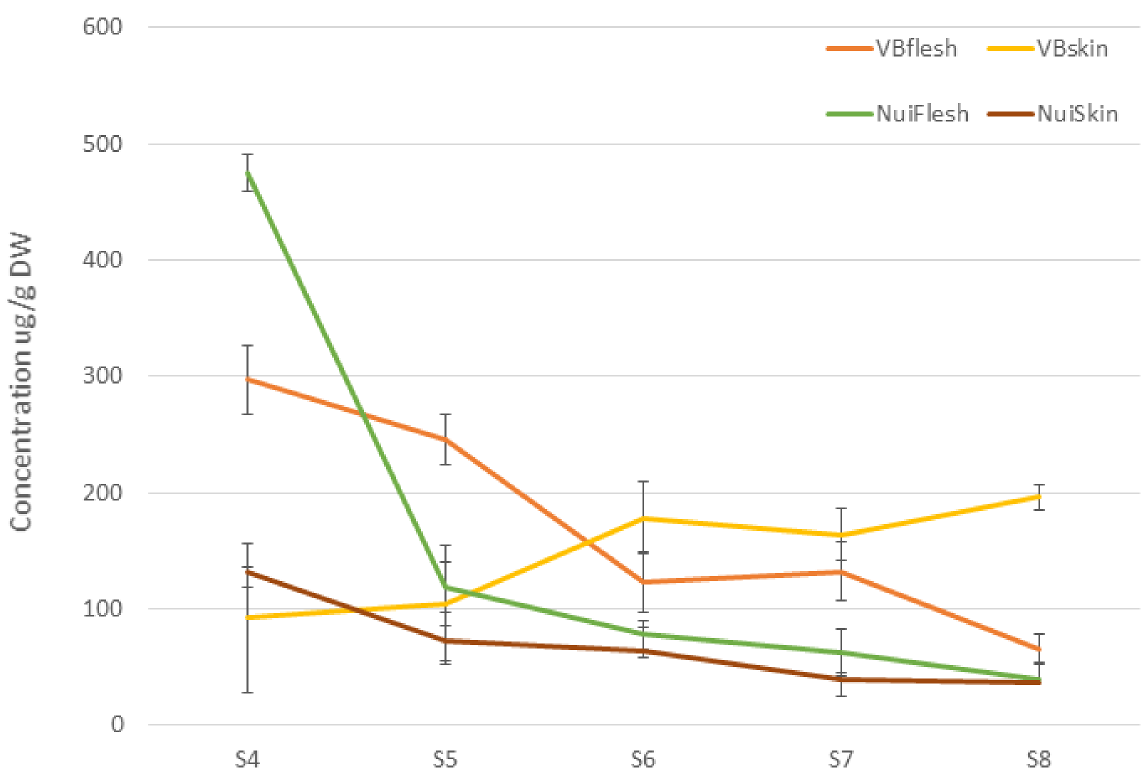

Figure S3:
Epicatechin accumulation in blueberry tissues during fruit maturation (S4 green/unripe- S8 purple/ ripe). VB: *Vaccinium virgatum* 'Velluto Blue'; Nui: *V. corymbosum* 'Nui'. Error bars indicate the Standard deviation of the mean of three replicates.

Table S3:

Overview of gene mapping results and functional annotation. Candidate genes were identified by mapping transcripts to *V. corymbosum* Reference Transcriptome (RefTrans V1) from the Genome Database For *Vaccinium* (GDV) and annotated using the KEGG/ KASS server. Genome location was then identified through reciprocal blast against the chromosome-scale genome assembly of tetraploid highbush blueberry from Michigan State University (MSU).

Name	Abbreviatio	KEGG	reftrans ID	MSU ID
CL1	4CL	K01904	v.corymbosum_GDV_reftransV1_0009935	maker-VaccDscaff46-augustus-gene-86.25
CL2	4CL	K01904	v.corymbosum_GDV_reftransV1_0018642	maker-VaccDscaff47-augustus-gene-2.17
CL3	4CL	K01904	v.corymbosum_GDV_reftransV1_0023569	maker-VaccDscaff37-augustus-gene-303.53
CL4	4CL	K01904	v.corymbosum_GDV_reftransV1_0030312	augustus_masked-VaccDscaff50-processed-gene-21.6
CL5	4CL	K01904	v.corymbosum_GDV_reftransV1_0033876	maker-VaccDscaff47-augustus-gene-69.25
CL6	4CL	K01904	v.corymbosum_GDV_reftransV1_0020364	maker-VaccDscaff48-augustus-gene-108.40
A3GT1	UFGT	K12930	v.corymbosum_GDV_reftransV1_0000638	maker-VaccDscaff28-augustus-gene-290.34
A3GT2	UFGT	K12930	v.corymbosum_GDV_reftransV1_0001574	maker-VaccDscaff6-augustus-gene-420.36
A3GT3	UFGT	K12930	v.corymbosum_GDV_reftransV1_0012230	maker-VaccDscaff37-augustus-gene-300.29
A3GT4	UFGT	K12930	v.corymbosum_GDV_reftransV1_0018085	maker-VaccDscaff19-snap-gene-64.36
A3GT5	UFGT	K12930	v.corymbosum_GDV_reftransV1_0035590	maker-VaccDscaff34-augustus-gene-294.29
ANR	ANR	K08695	v.corymbosum_GDV_reftransV1_0028563	maker-VaccDscaff19-augustus-gene-256.19
ANS	ANS	K05277	v.corymbosum_GDV_reftransV1_0032663	maker-VaccDscaff43-augustus-gene-236.29
C3.H	C3'H	K09754	v.corymbosum_GDV_reftransV1_0034725	maker-VaccDscaff1-augustus-gene-373.37
C4.H1	C3'H	K00487	v.corymbosum_GDV_reftransV1_0032452	maker-VaccDscaff24-augustus-gene-58.38
C4.H2	C3'H	K00487	v.corymbosum_GDV_reftransV1_0005046	augustus_masked-VaccDscaff33-processed-gene-307.8
CAD1	CAD	K00083	v.corymbosum_GDV_reftransV1_0016328	maker-VaccDscaff3-augustus-gene-213.23
CAD2	CAD	K00083	v.corymbosum_GDV_reftransV1_0016712	maker-VaccDscaff22-augustus-gene-306.29
CAD3	CAD	K00083	v.corymbosum_GDV_reftransV1_0021895	maker-VaccDscaff49-snap-gene-1.40
CAD4	CAD	K00083	v.corymbosum_GDV_reftransV1_0022475	maker-VaccDscaff22-snap-gene-141.41
CAD5	CAD	K00083	v.corymbosum_GDV_reftransV1_0004320	maker-VaccDscaff149-snap-gene-2.68
cCoAOMT1	OMT	K00588	v.corymbosum_GDV_reftransV1_0036845	maker-VaccDscaff34-snap-gene-139.31
cCoAOMT2	OMT	K00588	v.corymbosum_GDV_reftransV1_0038613	augustus_masked-VaccDscaff42-processed-gene-262.6
CCR1	CCR	K09753	v.corymbosum_GDV_reftransV1_0024732	maker-VaccDscaff4-augustus-gene-398.17
CCR2	CCR	K09753	v.corymbosum_GDV_reftransV1_0024733	maker-VaccDscaff8-snap-gene-149.28
CCR3	CCR	K09753	v.corymbosum_GDV_reftransV1_0024734	augustus_masked-VaccDscaff36-processed-gene-33.3
CCR4	CCR	K09753	v.corymbosum_GDV_reftransV1_0007209	maker-VaccDscaff27-snap-gene-293.30"
CHI	CHI	K01859	v.corymbosum_GDV_reftransV1_0019487	maker-VaccDscaff26-snap-gene-196.29
CHS1	CHS	K00660	v.corymbosum_GDV_reftransV1_0007549	augustus_masked-VaccDscaff9-processed-gene-64.0
CHS2	CHS	K00660	v.corymbosum_GDV_reftransV1_0017397	maker-VaccDscaff13-augustus-gene-46.32
CHS3	CHS	K00660	v.corymbosum_GDV_reftransV1_0018037	maker-VaccDscaff2-augustus-gene-67.14
COMT1	OMT	K13066	v.corymbosum_GDV_reftransV1_0014435	maker-VaccDscaff20-augustus-gene-5.31
COMT2	OMT	K13066	v.corymbosum_GDV_reftransV1_0033992	maker-VaccDscaff20-snap-gene-377.34
DFR1	DFR	K13082	v.corymbosum_GDV_reftransV1_0001248	augustus_masked-VaccDscaff13-processed-gene-166.8
DFR2	DFR	K13082	v.corymbosum_GDV_reftransV1_0026455	maker-VaccDscaff12-snap-gene-66.28
F3.5.H1	F3'5'H	K13083	v.corymbosum_GDV_reftransV1_0036273	maker-VaccDscaff29-augustus-gene-305.28
F3.5.H2	F3'5'H	K13083	v.corymbosum_GDV_reftransV1_0036277	maker-VaccDscaff19-augustus-gene-162.21
F3.5.H3	F3'5'H	K13083	v.corymbosum_GDV_reftransV1_0001233	maker-VaccDscaff10-augustus-gene-348.25
F3.5.H4	F3'5'H	K13083	v.corymbosum_GDV_reftransV1_0007214	maker-VaccDscaff24-augustus-gene-336.30
F3.H	F3'H	K05280	v.corymbosum_GDV_reftransV1_0007478	maker-VaccDscaff32-augustus-gene-159.26
F3H1	F3H	K00475	v.corymbosum_GDV_reftransV1_0033001	maker-VaccDscaff32-augustus-gene-323.35
F3H2	F3H	K00475	v.corymbosum_GDV_reftransV1_0038468	snap_masked-VaccDscaff7-processed-gene-48.23
FLS1	FLS	K05278	v.corymbosum_GDV_reftransV1_0030204	maker-VaccDscaff6-augustus-gene-163.26
FLS2	FLS	K05278	v.corymbosum_GDV_reftransV1_0032793	maker-VaccDscaff25-augustus-gene-225.24
HCT1	HCT	K13065	v.corymbosum_GDV_reftransV1_0006514	augustus_masked-VaccDscaff38-processed-gene-5.8
HCT2	HCT	K13065	v.corymbosum_GDV_reftransV1_0006673	maker-VaccDscaff24-augustus-gene-299.21
HCT3	HCT	K13065	v.corymbosum_GDV_reftransV1_0035212	maker-VaccDscaff27-snap-gene-291.30
LAR1	LAR	K13081	v.corymbosum_GDV_reftransV1_0032700	maker-VaccDscaff25-augustus-gene-293.25
LAR2	LAR	K13081	v.corymbosum_GDV_reftransV1_0033478	maker-VaccDscaff28-augustus-gene-232.26
LAR3	LAR	K13081	v.corymbosum_GDV_reftransV1_0007959	maker-VaccDscaff9-augustus-gene-315.13
PAL1	PAL	K10775	v.corymbosum_GDV_reftransV1_0003392	maker-VaccDscaff19-augustus-gene-331.32
PAL2	PAL	K10775	v.corymbosum_GDV_reftransV1_0013816	maker-VaccDscaff10-augustus-gene-222.29
PAL3	PAL	K10775	v.corymbosum_GDV_reftransV1_0024098	maker-VaccDscaff29-augustus-gene-173.18
VcbHLH075	bHLH		v.corymbosum_GDV_reftransV1_0002395	maker-VaccDscaff17-augustus-gene-313.25
VcbHLH1	bHLH		v.corymbosum_GDV_reftransV1_0032614	maker-VaccDscaff22-snap-gene-21.29
VcbHLH2	bHLH		v.corymbosum_GDV_reftransV1_0008144	maker-VaccDscaff19-augustus-gene-381.30
VcMYB4	Repressor		v.corymbosum_GDV_reftransV1_0019415	maker-VaccDscaff37-augustus-gene-96.16
VcMYBA	Activator		v.corymbosum_GDV_reftransV1_0025517	maker-VaccDscaff1486-snap-gene-0.3
VcMYBC2	Repressor		v.corymbosum_GDV_reftransV1_0001734	maker-VaccDscaff28-augustus-gene-197.19
VcMYBPA1	Activator		v.corymbosum_GDV_reftransV1_0038996	maker-VaccDscaff39-snap-gene-168.25
VcMYBPA2a	Activator		v.corymbosum_GDV_reftransV1_0003384	maker-VaccDscaff32-augustus-gene-55.27
VcMYBPA2b	Activator		v.corymbosum_GDV_reftransV1_0015556	maker-VaccDscaff33-snap-gene-307.38
VcMYBR3	Repressor		unknown	maker-VaccDscaff4-snap-gene-174.23
VcWDR1	WDR		v.corymbosum_GDV_reftransV1_0019141	maker-VaccDscaff28-augustus-gene-346.33

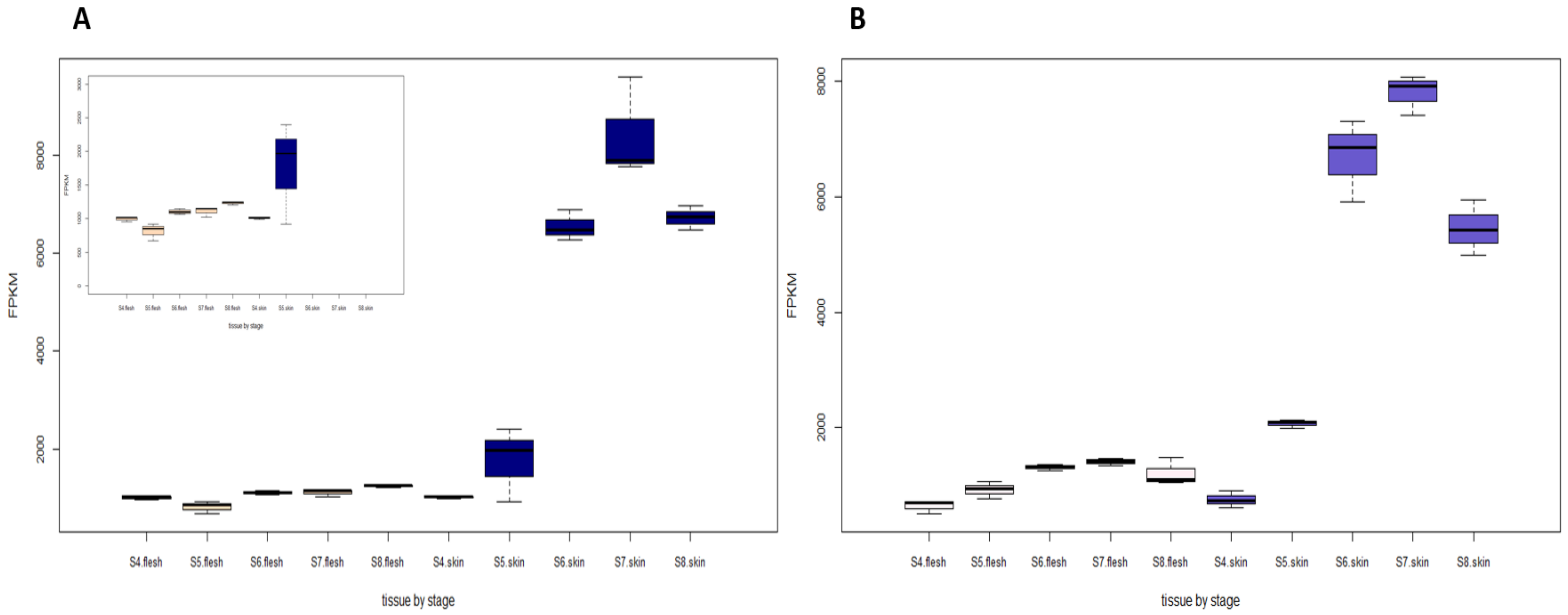

Figure S4: Combined transcript counts (FPKM) of biosynthetic genes and transcription factors for *Vaccinium corymbosum* 'Nui' (A) and *V. virgatum* 'Velluto Blue' (B) fruit tissue (pink: flesh; purple: skin) during maturation development (S4 green/unripe- S8 purple/ ripe).

TableS4:
Summary of factorial Analysis of Variance testing the effect of blueberry cultivar (*Vaccinium virgatum* ' Velluto Blue' and *V. corymbosum* 'Nui'), tissue type (fruit skin, fruit flesh) and their interaction on gene expression of candidate transcription factors. Normalised (FPKM) values were Log10 –transformed for data normalisation and P-values adjusted (Padj) using Benjamini Hochberg method for False discovery rate correction. Significant (α=0.05) results are highlighted in red.

	**Cultivar**		**Tissue**		**Interaction**	
	F-value	*P_{adj}-value*	*F-value*	*P_{adj} -value*	*F-value*	*P_{adj} -value*
bHLH075	16.10	5.41 x 10^{-04}	0.24	0.72	0.00	0.99
bHLH1	0.39	0.63	1.16	0.36	2.41	0.19
bHLH2	24.55	3.3 x 10^{-05}	71.32	1.2 x 10^{-10}	3.44	0.12
MYB4	7.45	0.02	22.28	6.7x10^{-05}	4.48	0.07
MYBA	5.07	0.06	20.66	9.9 x10^{-05}	0.13	0.8
MYBC2	5.23	0.05	47.50	3.4 x10^{-08}	1.45	0.33
MYBPA1	0.01	0.97	175.23	2.3x 10^{-17}	1.20	0.36
MYBPA2a	2.55	0.19	5.65	0.05	0.90	0.42
MYBPA2b	45.46	5.1 x 10^{-08}	106.35	1.6x 10^{-13}	1.35	0.35
MYBR3	5 x 10^{-06}	0.99	20.64	9.9x 10^{-05}	2.40	0.19
WDR1	120.10	2.5 x 10^{-14}	6.10	0.04	14.69	8.9 x 10^{-04}

Figure S5A:
bHLH and WDR1 gene expression in *Vaccinium virgatum* ‘ Velluto Blue’ (red) and *V. corymbosum* ‘Nui’ (blue) fruit tissue types over development (S4-S8)

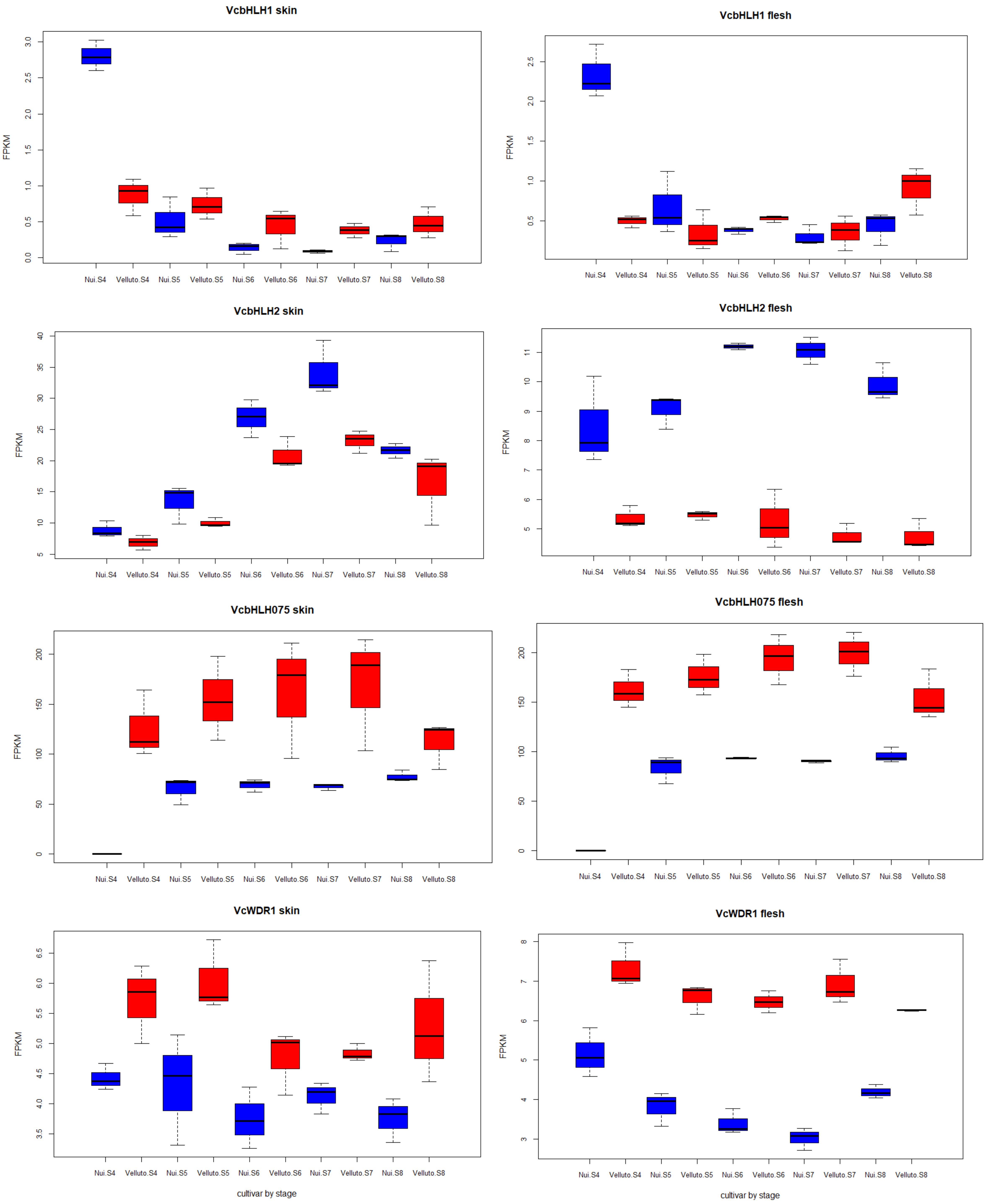

Figure S5B:
MYB activator gene expression in *Vaccinium virgatum* ‘ Velluto Blue’ (red) and *V. corymbosum* ‘Nui’ (blue) fruit tissue types over development (S4-S8)

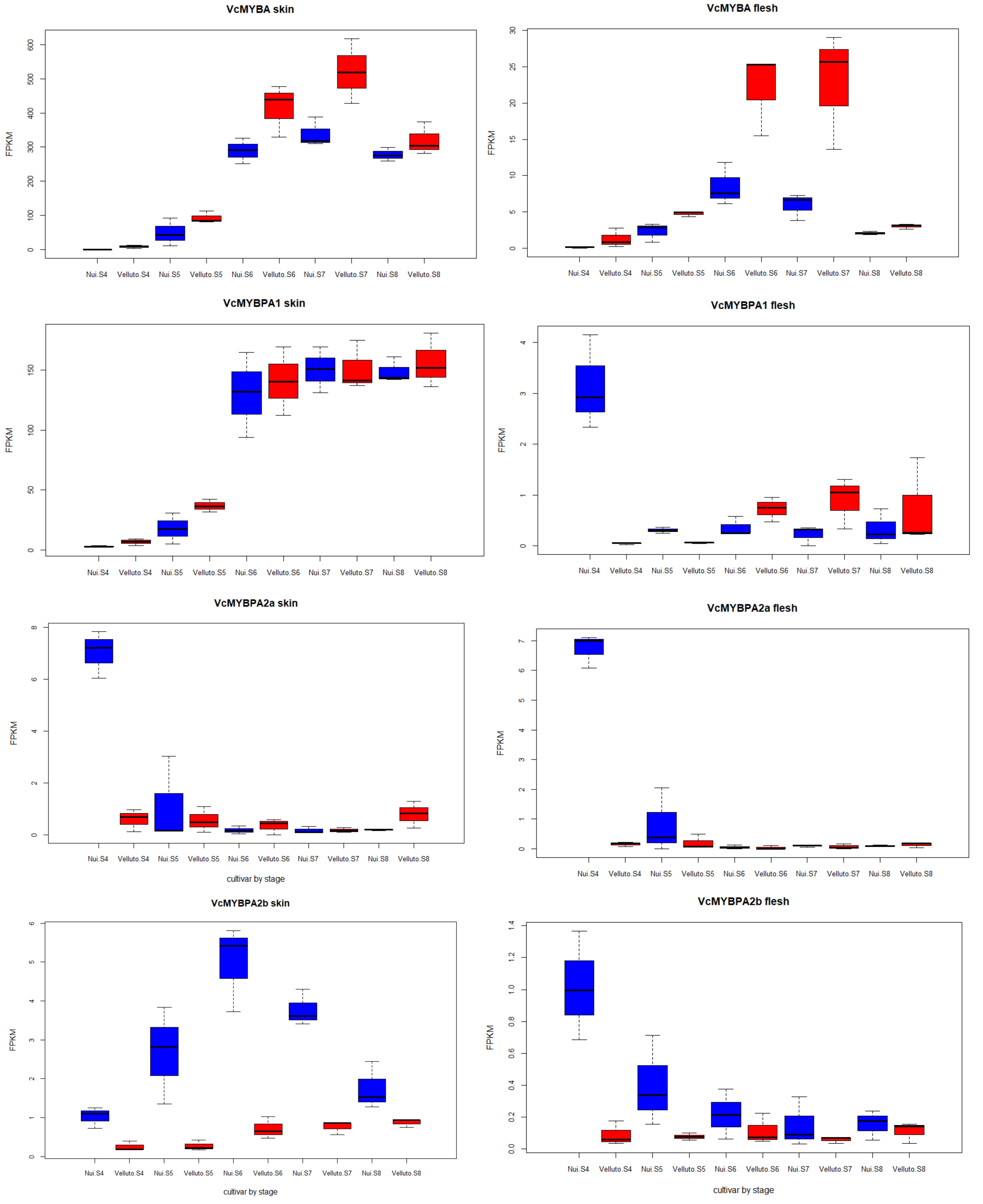

Figure S5C:
MYB repressor gene expression in *Vaccinium virgatum* ‘ Velluto Blue’ (red) and *V. corymbosum* ‘Nui’ (blue) fruit tissue types over development (S4-S8)

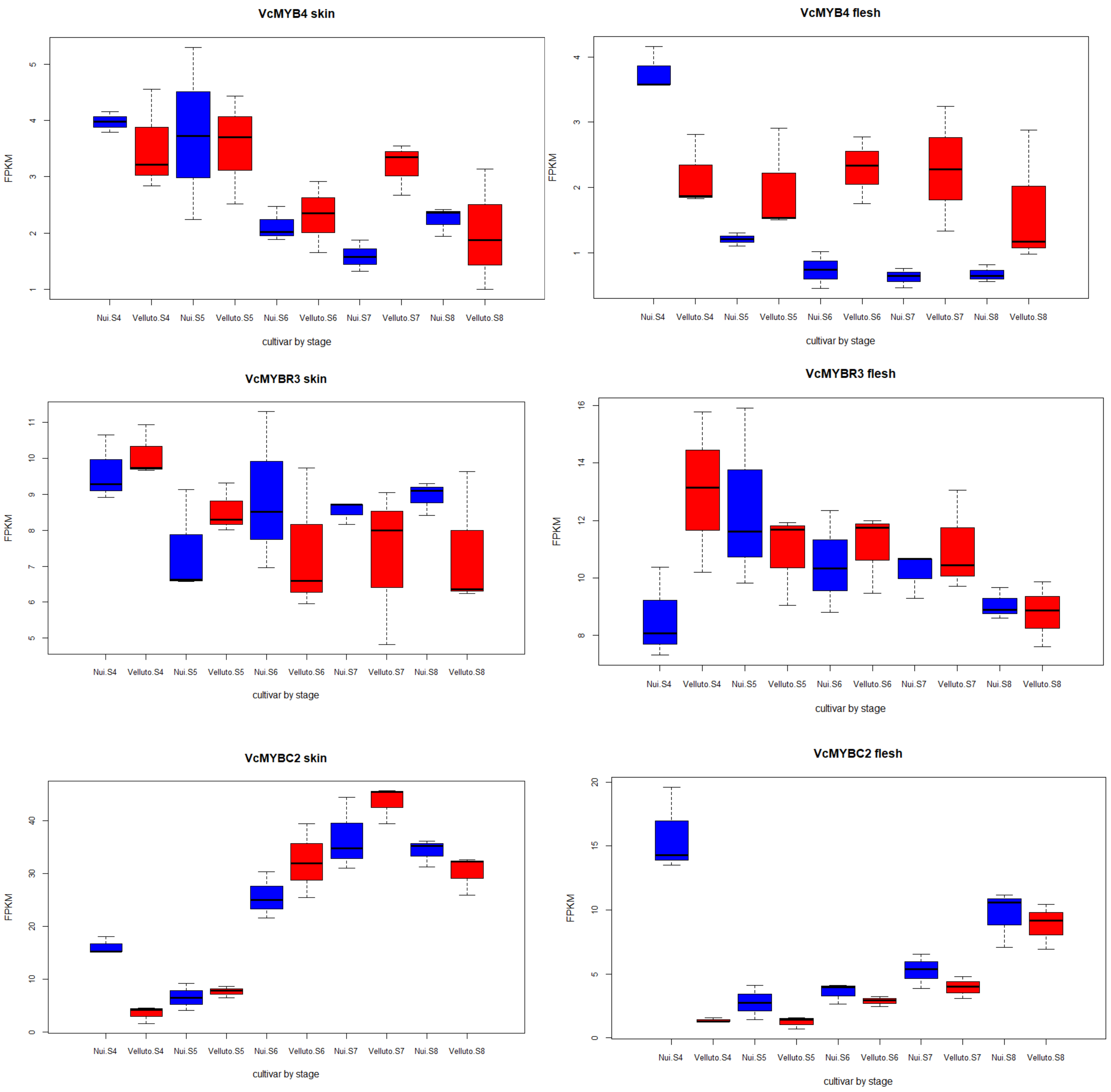

Supplement: FIGURE S1 — Concentrations of total measured polyphenols in blueberry fruit tissues during. [file Data_Sheet_1.pdf]
