# Supplementary material for: Spatiotemporal Modulation of Flavonoid Metabolism in Blueberries
Source: Front Plant Sci. 2020 May 13;11:545. doi: 10.3389/fpls.2020.00545 (PMC7237752; doi:10.3389/fpls.2020.00545)
Supplement: APPENDIX — Coding sequences of genes listed in Supplementary Table S3. [file Data_Sheet_2.PDF]

>VcA3GT1 K12930 maker-VaccDscaff28-augustus-gene-290.34

ATGACCAAAATATCAAAAGACCACCGCCACGTAGCCGTTCTGCCATTCCCATTCTCCAGCCACGCCGGCCGCCTC
CTCAGCCTCGTTACCCGCCTCGCCACCGCCGCAGGCCCAAACATCACTTTCTCCTTCTACACCACTTCCAAATCACTCGAAA
CCTTATTCGCATCGGCGAAGAGAGTCCCCGGAAACATAAAGCCGTACGTGGTGTCCGACGGGGTGCCGGAGGGGTACGT
GTTCTCCGGGAAGCCCCTGGAGGATATTAACTTGTACCTTACGTCTGTGGCGGAGGGGGAGAGCCTTAAGGGAGTTTTG
AAGGCGGCCGAGGCGGAGACAGGGCAGAGGATTGGGTGTGTTATGTCGGATGCGTTTCTGTGGTTCGCCGGGGATTTG
GCGGAGGAGATGGGGGTTCCGTGGGTTCCGCTTATGTGCGGGGGTGCTAACTCTGTGTCGGCTCATTTTTACACTGACCT
GATTAGGGAGACTGTTGGCATGCATGACATTGCCGGGCGGGGAAACGACGTCGTGAAATTCATCCCAGGATTTTCTGAG
CTACGCCTTGGGGACTTGCCCACGGGAATCCTGTTTGGAAACTTGGAATCTCCATTCGCCGTCATGCTACACAAAATGGGC
CGGGCTTTTCCGAAAGCGACCGCGATTGTTATCAACTCCTTTGAAGAACTAGACCCCGAACTCAATCGAGATCTCAAGTCC
AAGTTTAGAATGGTTCTCAATGTGAGCCCATTTAACTCAAAATCATTGTCGTCGCCGCCGCCGCCGTACTCGGACGAGTAC
GGATGCATCCCGTGGTTGGACAACCACAAATCCGCCTCCGTTGCCTATATCGCCTTTGGAACATTAGCTACACCGCCACCG
GTTGAGATTGCTGCATTGGCTGAAGCACTAGAAGCCTACCGCACTCCGTTTCTCTGGTCTCTCAAAGATGATTCGAAAGAG
TTTCTTCCGGAAGGATTCATGAAGAGAACTAGCGAGCTGGGAAAAATCGTGCCGTGGGCACCTCAAGAACAGGTTCTGTC
ACATAGTTCAGTAGGAGTTTTCGTGACCCACTGCGGGTGGAACTCGGTACTGGAGAGCATTGCGGCCGGTGTGCCACTAA
TCGGGAGGCCATTCTTCGGGGATCACCACCTAAACGCGTGGGAGGTGGAAAACGTGTGGAAGATTGGTGTGAGGTTGG
AGGGCGGAGTTTTCACAAAAAATGGCACAGTTTCTGCTCTTGAACTGGTTTTGAATCATGAAAAGGGCAAAGAATTGAGG
GAGCAAATTGTAAAGTTCAAAGAGCTTTTTTGGAAGGCTGTTGAACCACAAGGGAGCTCAACCCAAAATTTCAATACTTTG
TTGGAGGCAGTAACAGGGTACAATCTTTA

>VcA3GT2 K12930 maker-VaccDscaff6-augustus-gene-420.36

ATGTCCAACTTCTCAAAAGACCGGCACGTCGCTGTTTTGCCATTCCCATTCTCCACACACGCCGCCCCAATCCTCA
GCATCATCCGCCGCTTTGCCTCCGCCTCCCCCGACGTCACTTTCTCCTTCTTCAGCATTCCTCAATCCATCCAAACCCTGTTCC
CATCCGAAAACCCCGACAGCAACATAAAGCCCTACGTCGTGTCGGATGGGGTGCCGGAGGGGTACGTGTTCTCAGGGAA
GCACCATGAAGACATCAACTTGTTTCTTGCGGCGGGGAAGGAGAGCTTGAAGGCAGGGATGAAGGCAGCCGAGGCGGA
GATCGGGCGGAGGATTGACTGTGTGGTGGCGGATGCGTTTTTGTGGTTTACGCAAGAGTTGGCGGAGGAGATGGGGGT
GCCATGGATCACGCTTTGGGTTTCAGGGGCATGCTCTCTGTCGGCTCATTGTTATACTGATCTCATTAGGGAAACTGTGGG
CATGCATGACATTGCGGGACGCGAAAACGAAATCGTGAAATTCGTCCCGGGATTTTCAGAGGTACGACTCGGGGACTTG
CCCAGCGGAGTCGTGTATGGAAACTTAGAATCACCCTTCTCAATGATGCTGTACAATATGGGCCAAGTTCTGCACAAAGC
AACCGCAGTTGCCATAAACTCCTTTGAAGAATTAGAGCCTGAACACAACAAAGTTCTCGAGTCGAAGTTTAAGAAGCTCCT
CAATTGTGGTCCGTTCAACTCAATATCACCGCCACCACCACCGTCGTCCAACTTGGACAAATACGGCTGTATCCCGTGGCT
GGACCAGCACAAAACTCGTTCAGTAGCGTATATCGGCTTCGGATCAGTGGCTACACCGCCACCGGTTGAGATAGCGGCAT
TAGCTGAAGCACTAGAAGCTAGCGGCACTCCGTTCCTCTGGTCTCTCAGAGACAATTTCAAAAAGCATTTACCAGAAGGAT
TCCTGAAGAGAACAAGTGAGCTAGGAAAAATCGTGGCATGGGCTCCTCAAGTGCAAGTTTTAGCACACAGCTCAATTGG
GGTTTTCATAAACCACTGCGGTTGGAATTCAGTACTGGAGAGCATTGTGGCTGGCGTGCCAATCATCGGTAGGCCGTTCT
TCGGGGATCATCAGGTGGACACGTGGATGGTGGAAAATGTGTGGAAGATTGGTGTGAGAGTGGAGGGTGGAGTCTTCA
CAAAAAGTGGCACAATGTCTGCCCTTGAACTGGTTTTGTCCCAAGAAAAGGGGAAGGAATTGAGGGAACAAACTGGAAA
GTACAAAGAGTTTGCTTTGAAGGCTGTTGGACCAAAAGGGAGATCAACTCAAAATTTAAACACATTGCTGGAGTTAGTGA
GAGGGTACAACATTTAG

>VcA3GT3 K12930 maker-VaccDscaff37-augustus-gene-300.29

ATGCAAGTCCCCAATGAAGGTCACGTTGCCGTCCTAGCATTCCCATTCGGCACCCACGCCACCCCTCTCCTCACC
CTCGTCCGCCGCTTATCCGCCACCGCGCCCAACCTAAAATTCTCATTCCTCAACACGCCCGCATCCAACCAAAAAATATTTT
CCAAAATCAAACCCGATGACTACCAAAACATCAATCCATACGACGTGGATGACGGCGTCCCTGAGGGGCACGTGTTTTCA
GGCCATCCTTTGGAGGCCGTGGAACTTTTCCTAGGGGTAATGCCGGAAAACTTTAAAAATGGTTTGGAAGAGGCTGTGG
AAAAAACAGGCGTCCAGATCACGTGCTTGTTGACCGATGCGTTCTATTGGTTTGCGGCCGATATGGCGGCTGAAATGGGT
GTCCCTTGGGTGGCGTTTTGGACCGCCTCGCCTTGCGCTATCTCCGTTCATATGTATACCGATGTCATTTTGAGCACGTTGA
AAGGCTATACAGAAGAAAACGGAGATCAAACTCTCAATTTCATCCCAGGAATGTCCGCAATTTACGCCAAAGATATACCA
GGAGGGATCAACCAAGAAGTGCCATTCAACATCTTGTTACACAAAATGGGACAAACAATACCAAGAGTAACAGCCCTCGC
AATAAACTCATTCGAAGAAATTGATCCGATTATCACGAACGATCTCAAGTCGAAGCTGAAAATGGTGATGAATATAGGCC
CCTTTGATCTCGCCTTGCCACCGAAATCATTTCCGGATGAGAGCGGATGCATACCGTGGCTGGACGAGCACGAGAAAGCG
TCCGTGGCGTACCTAAGTTTCGGTACCCTTTTGACGCCTCCGCCGGATGAGCTGATTGCATTGGCAGAGGCACTGGAAAC
TCAAAGGGTGCCGTTTTTGTGGTCTTTTAGAGACAGTTCAAAGCTGCCGTTGCTGGAAGGATTTCTTGAAAGAACGAGCA

CTCTTGGGAAGGTGGTTCCATGGACACCTCAATTACAAGTCCTAGAACACCCATCGGTCGGAGTGTTCATAACTCATGCCG
GGTGGAATTCGGTTTCCGAGAGCATTGCCGGTGGCGTGCCAATGATATGCCGGCCTTTTTCGCGGATCAAGGGCTGAAC
AGCCGGCTAGTTGAGGAAGTTTGGAAGATTGGTGTAAGCGTCGATGGTGGAAATTTCACTAAATCGGGAACTACAAAAG
CCCTAGAACTCGTTTTCTCGTCGGAAAAAGGGGTGGAAATGAGAGAGAATATTGGTAATCTTAAAGAGAAAGCTAAAAT
AGCTGTGGGTAGTGATGGGAGCTCCACCAAGAATTTCATAAGTTTGGTGAAGGTGGTTGCAGGCTGTGAGAATGCTTAA

>VcA3GT4 K12930 maker-VaccDscaff19-snap-gene-64.36

ATGACCAATGTTGTCTCGAAAGACCGGCATGTGGCCGTTCTGCCATTCCCATTCTCCAGCCACCCCGGCGGCCTC
CTCAGCGTCGTTACCCGTCTTGCCACCGCCGCCCCAAACGTCAGTTTCTCCTTCTACAGCACTTCCAAATCCATCGAAACCT
TATTCGCACCTGCGAAGGGAGTCCCCGGCAACATAAAGCCGTACGTGGTGTCGGACGGGGTGCCGGATGGGTACGTGTT
CTCCGGGAACCCTCTGGAGGCTATTAACTTGTACATTGCGGCTGTGGAGGAGGAGCGGAGCATTAAGGCAGTTTTGAAG
GCGGCCGAGGCGGAGACAGGGAAGAGGATTGGGTGTGTTATGTCGGATGCGTTTCTGTGGTTTGCCGGAGATTTGGCG
GAGGAGATGGGGGTTCCGTGGGTATCGTTTTGGTGCGGGGGTGCTAACTCATGTGCTGCTCATTTTTACACTGACCTGAT
TAGGGATACTGTTGGAATGCATGATATTGCCGGGCGGAAAAATGACGTCGTAAATTTCGTGCCGGGATTTCCAGAGCTAC
GTCTCGGGGATTTGCCCCCGGAAATCCTGTTTGGAAACTCGGAATCTCCATTCGTAATCATGCTGCACAAAATGGCCCGG
GCTCTACCCAAAGCTACCGCTGTTTTTGTCAACTCCTTTGAAGAAATAGTTCCTGAAATCAACCAAGATCTCAAGTCCAAGT
TGAAAATGCTCCTCAACGTTGGCCCATCCAATTTACTATCATCTTCGTCACCGCCGCCACCCTCCTCGTACTCAGACGAGTA
CGGATGCATCCCGTGGTTGGACAATTTCAAAGCAGTCTCAGTTGCCTATATCGGCTTTGGAACAATACTTACACCGTCACC
AGCAGAGATTGTGGCATTAGCTGAAGCACTAGAAGCTAGCGGCACTCCGTTTCTCTGGTCTCTCATAGATAATTTGAAAG
AGTTTTTTCCACAAGGATTCATGGAGCGAACAAGCAAGCTAGGAAAAATCGTGCCGTGGGCACCTCAGCAACAAATTTTG
GCCCATAGTTCAGTAGGAGTTTTCGTAAGTCACTGTGGGTGGAACTCGGTATTGGAGAGCATTGTGGCAGGTGTGCCTCT
GATTGGGAGGCCATTCTTCGCAGATAATCTACTAAACGCGTGGATGGTGGAAAAGGTGTGGAAAATTGGTGTGAGGTTG
GAGGGAGGGGTTTTCACAAAAAATGGCACAATGTTGGCTCTTGAACTGGTTTTGAATCATGAAAAGGGTAAGGAAATGA
GGGAGCAAATCGGAAAGTACAAAGAGCTTGCATGGAAGGCTGTTGGACCAGAAGGGAGCTCAACTCAGAATTTCAATAC
TTTGCTGGAGGTAATAACAGAGTACAATCTTTAG

>VcA3GT5 K12930 maker-VaccDscaff34-augustus-gene-294.29

ATGGCTGAAACAAGCTGCCACTCCCAACCAAAACAACTGCACATAGCAGCCCTTGCCTTCCCCTTCGGCACCCAC
GCCGCCCCTCTCCTGACCCTCCTTCGCCGCTTAGCCACTGCCGTACCACCCACCACAGTTTTCTCATTTTTCAGCACTGAAAA
GTCAAACACCCAAACATTTTCCAACAAAGCAGATCTCCCATTTGGGACGTCCATAAAACCTTACAATGTGTGGGATGGTAT
GCCGGAGATGTCTACGGCAGTGGATCAGCAACACATAAATCCCGGCAATGCCCAAGTGGCCGGTCCCGCGTTTTTCCTCG
GGGCGGTGCCGGGAAACTTCAAGAAGGCAATGGGAGAGGCGGAGCAGGAGAGCGGTGTGAAGATAAGTTGCATTCTG
AGTGATGCATTCTTGTGGTTTTCGGGTGATTTGGCGGCGGAGATGGGGGTGCCTTGGGTGGCATTTTGGACTGCCGGTTC
TTGCTCTCTCTCTGTCCATATGTGTACCGATGAAGTTAGGAAGACACTCGAAATGCCTGGAAGTACTCTTCAGCAAGATCT
TTCTTTCATCCCCGGAATGTCAGCAGTGCACATCGATGATTTACCGGAAGGAATACTCTCCGGAAACTTGGAATCGCCGAT
CGCTCAAATGCTTTGGAAAATGGGACTGAACTTGTCAAGAGCAACTGCAGTTGTCCTAAACTCATTTGAAGAGCTAGACC
CTACAATCACAAATGACCTGAAGTTGAAGTTGCGAAAAGTGCTCCACGTCGGCCCTTCAACCCTAGCATCATCATCACCAC
CACCGGGGCTACCGACAATTTCGGACGAAAATGGCTGCCTGTTGTGGTTGGAAAAGCAAAAACCATCATCTGTTGCTTAC
ATTAGCTTTGGCACCATGATGACACCACCCCCTAATGAGTTGCTAGCATTGGCGCAAACACTTGAAGCTCTTAAACTCCCTT
TCCTTTGGTCACTCAGGGACCATTCGCGACACTTTTTGCCCCAAGGATTTTTCGAAAGCACGGTGTCTTTCGGTAAAGTCGT
TTCATGGGCACCACAATCGCAAGTCTTGGCACATCCGTCCGTCGGAGTTTTTGTAACGCATTGTGGGTGGAACTCAATCTT
GGAGAGCGTTGCCGGAGGTGTGCCGATGATCTGCAGGCCGTTCTTTGGGGATCAGACGTTGAACAGTCGGATGGTACAG
GATGTGTGGCGAATTGGGGTCAAGGTTGAGGACGGAGGAGGGTTCACAAAATCTGGAACAACGAGCGCTTTGGAACTT
GTTTTGTCGAGCGGGGAAGGAAGGAAAATGAGAGAGAACATTAGAGGGCTCAAAGAGAGCGCGGTGAATGCCGTTGG
ACCGAATGGAAGCTCGACTGCGAATTTCGAAACTTTGGTAGAGGTGATCAAAACTTGCAAT

>VcANR K08695 maker-VaccDscaff19-augustus-gene-256.19

ATGGCAGCTTCGAAGACGGCTTGCGTCATCGGCGGCAGCGGGTTCGTGGCTTCGACCCTGATTAAGCTTTTGTT
ACAGAAAGGCTACGCCGTCAACACCACTGTTAGGGATCCCGACAATCACAAGAAGACCTCCCACCTCGTAGAATTTCAGA
GTCTGGGGAGCTTAAACATATTCAAAGCAGATCTTACTGATGAAAAGAGCTTTGATGCCCCAATAACAGGTTGTCACCTG
GTCTTCCATGTTGCAACTCCAGTCAACTTTGCTTCAGAGGATCCAGAGAATGACATGATAAAGCCGGCAATCCAAGGAGT
GCAGAATGTTTTAAAAGCCTGTGCAAAAGCAGGAACGGTTAAGCGCGTTGTTTTAACATCCTCAGCAGCTGCTGTAACGA
TCAATACACTCAATGGGACTGGCATCGTCATGGATGAGAGTCACTGGACCGATGTCGAATTTTTAAGTTCTGCAAAGCCAC
CCACCTGGGGGTATCCTGCATCAAAGACCCTAGCGGAGAAAGCAGCTTGGAAATTTGCCGAAGAAAACGACATTGATCTT

GTTACCGTCATCCCTAGTCTTATGGCTGGTGTTTCTCCCACAATAGACGTTCCTAGTAGTGCTTTTCTTGCCATGTCCTTAAT
CACAGGGAATGAGTTCCTTATAAATGCATTGAAAGGTATGCAAATGCTCTCAGGTTCTATCTCCATATCCCATGTCGAAGA
TGTTTGTCGCGCCCATATATTTGTGGCCGAAAAAGAATCGGCTTCTGGTCGTTATAATTGCTGTGCCATCAATACCAGTGTT
CCAGAGCTTGCCAAGTTCCTCAAGAATAGATACCCACAGTACAACATCCCTACTGACTTTGACGATTTCCCTTCCAAAGCCA
AGTTGATCGTTTCATCTGAAAAGCTTATCAAAGAGGGATTCAGCTACAAGTATGGTATTGAAGAAATTTACGACCAATGC
GTGGAGTACTTCAAGTCTAAGGGGATTTTGCAGAACTGAAATCAACTGATATTTACCTCTCCTTTTCTAATATGCCACTGTG
TGTCTGTGTGGTTGGTGTTCACCTAGCAATGGTTTTACTTGGTTGTGCGAGTAGTACGAATAATAACAATTTCCCTTTCGGA
GTTTCTCCGCGTGACTTGTCGAAAGACAGATTTTCAAAGATCTCTGGTTGA

>VcANS K05277 maker-VaccDscaff43-augustus-gene-236.29
ATGGTGAGTACAATGGTTGCTGCTCCCAGTCGCGTCGAGAGCCTTGCAAGCAGCGGCATCCAGTCCATCCCGA
AAGAATACGTGAGGCCTAAAGAGGAGCTCACCAGCATCGGCAACATTTTTGAGGAGGAGAAGAAGCACGAAGGGCCTC
AGGTGCCAACCATTGATCTAGAAGACTTGGTATCGGAGGACAAAGAGGCCAGAGAGAGATGCCACGAGGCGTTGAAGA
AGGCGGCGACAGAGTGGGGGGTGATGCACCTAGTGAACCACGGCGTCCCCGAAGAGCTGATGGACCGGGTTAGGGTG
GCCGGAGAGGGGTTCTTCAATCAGCCCGTGGAGGAGAAGGAGAAGTATGCTAATGACCATGATACTGGGAATTCTGGGA
AGATACAGGGGTATGGGAGTAAACTGGCAAACAATGCCAGTGGGCAGCTGGAGTGGGAGGACTATTTCTTCCACACTGT
TTACCCTGAGGACAAGAGGGACATGAAGATTTGGCCCAAGAACCCATCCGACTACATTCCGGCGACAAGCGAGTATGCG
AATCACCTGAGAGCCCTAACAACCAAGGTCCTATCGGCCCTCTCCGTCTGCTTGGGATTGGAAGAAGACAGGCTAGAAAA
AGAAGTTGGCGGAAAGGACGAACTCGTCATCCAAATGAAAATCAACTACTACCCAAAATGCCCTCAACCGGAGCTTGCCT
TGGGCGTCGAAGCCCACACTGATGTCAGCGCCCTCACCTTCATCCTCCACAACATGGTCCCCGGCCTGCAACTCTTCTACG
AGGGCAAATGGATCACAGCAAAATGTGTCCCTAACTCCATCATTATGCACATTGGCGACACGGTCGAGATTTTGAGCAAT
GGGAAGTACAAGAGCATTCTCCACAGGGGGCTTGTCAATAAGGAAAAGGTTAGGATTTCGTGGGCGGCTTTTTGCGAGC
CGCCAAAGGAGAAGATCATCCTGAAGCCGCTCCCGGAGACCATGTCGGAGACCGAGCCGGCAAGGTATCCGCCGCGTAC
CTTTTCTCAGCACATCGAGCACAAGTTGTTCAGGAAGACCCAGGCACTTAATGGGGCTTAA

>VcC3.H K09754 maker-VaccDscaff10-snap-gene-65.37
ATGGGATTGTCTCTACTACTCCTCTTCTCAATCACTCTGATAAGCTTCGTCGTCTACTACCTCTACCCACGGCTGA
GATACAAGCTCCCACCGGGCCCCCGCCCGCTGCCATTCGTCGGAAACCTCTACGACATAAAGCCGGTGAAGTTCCGATGC
TTCGCGGAGTGGGCCCAGGTCTACGGCCCGATCTTTTCGTACTGGATGGGCTCGACGCTAAACGTGGTCGTTTCGAGCTC
GGCGCTGGCGAAAGAGGTGCTCAAGGACAAGGATCAGCAGCTGGCGGATAGGCATAGGAGCAGATCGGTGGTCAGGTT
TAGCAAAGATGGGCAGGACCTCATTTGGGCTGATTATGGGCCTCACTATGTGAAGGTTAGGAAAGTTTGTACACTTGAAT
TGTTCACTCCGAAGAGGCTTGAGTCCCTTAGGCCCATTAGGGAAGAAGAGGTTACAGCCATGGTTGAGTCCTTGTACAAA
GACTGCACCAATTCTGAAAACTCGGAGAAAAGTTTGCTAGTGAGGAAATACTTGGGAGCGGTAGCATTCAACAACATAAC
AAGACTGACATTTGGGAAACGGTTCGTGAATTCCGACGGCGTTTTGGATGAACAAGGACTGGAATTCAAGGCGATTTCG
GAGAACGGCCTGAAACTGGGGGCGACACTGGCTGCGGCGGAGCACATCCCCTGGCTCCGGTGGATGTTCCCACTCGAGG
AAGAGGCTTTCGCCAAGCACGGGGCACGGCGGGACAGACTCACCAGGGTCATCATGGATGAGCACACCGCCACCCGCCA
GAAGAGCGGTGGCGCTGCCAAGCAGCATTTTGTGGATGCGTTGCTTACATTAAGGGAGAAGTATGATCTGAGTGATGAC
ACCATCATTGGGCTGCTTTGGGACATGATAACTGCCGGCATGGATACTACTGCTATATCAGTTGAGTGGGCAATGGCTGA
GGTGATCAAGAACCCTAGGGTGCAACAGAAGGTTCAAGAGGAGCTAGACCGGGTTGTTGGGTACGAGCGAGTCATGAC
CGAATCCGACTTCTCCAGTCTCCCATACCTGCAATGCGTAACCAAAGAGGCGATGAGGCTACACCCTCCAACCCCTCTGAT
GCTTCCCCATAGGGCTAATTCCAACGTCAAGATTGGCGGGTACGATATCCCTAAAGGATCCAATATGCACGTAAATGTGT
GGGCTGTGGCCCGTGACCCAGAAGTTTGGAAAAACCCAGCCGAGTTCCGACCAGAGAGGTTTTTGGAAGAGGATGTTGA
TGTGAAGGGTACCGATTTTCGATTGTTGCCTTTCGGAGCTGGCCGACGGGTCTGTCCTGGGGCTCAACTCGGGATCAACT
TGGTGACGTCAATGCTCGGTCACCTTCTCCATCATTTTTGGTGGACCCCACCAGAAGGTGTGAAGCCGGAAGAGATAGAC
ATGTCAGAGAACCCGGGAATGGTGACTTACATGAGAACCCCTTTACAAGCTGTTGCTAAGCCAAGATTGCCTGCTCACCTC
TACAATCGTGTTGTTTTTGACATGTAA

>VcC4.H1 K00487 maker-VaccDscaff24-augustus-gene-58.38
ATGGATCTCCTCCTCCTAGAAAAAACCCTCCTAGCCCTTTTCTTTACCACACTAGTCGCCATCACCGTCTCCAAAT
TATGCGGCAAGCGCTTCAAGCTCCCTCCAGGACCCATACCAGTGCCCGTATTCGGAAACTGGCTCCAAGTCGGAGACGAC
TTGAACCACCGGAATTTGACCGATTTAGCCAAGAAATTCGGTCAAATACTCCTCCTCCGTATGGGCCAACGCAACTTAGTC
GTGGTCTCCTCCCCGAACCTAGCCAAAGAAGTCTTACACACCCAAGGCGTCGAGTTCGGCTCGCGTACCCGAAACGTCGT
GTTCGATATCTTCACCGGGAAGGGCCAGGACATGGTGTTCACGGTGTACGGCGAGCACTGGAGGAAGATGAGAAGGAT
CATGACCGTCCCTTTTTTTACCAACAAGGTTGTGCAGCAGTACAGGTGCGGGTGGGAGGACGAGGCGGCCGGGTCGTG

GAGGACGTGAGGAGGAACAAGGAGGCTGCCACCGATGGGATTGTTTTGAGAAGACGGTTGCAGCTTATGATGTACAATA
ATATGTACAGGATTATGTTTGATAGGAGGTTTGAGAGTGAGGAGGATCCGTTGTTTGTGAAGCTCAAGGCGTTGAATGG
AGAGAGGAGTCGGTTGGCTCAGAGCTTTGAGTATAATTATGGTGATTTTATTCCGGTTTTGAGGCCTTTTCTGAGAGGGT
ATTTGAAGGTTTGCAAGGATGTCAAGGAGAGGAGGTTGCAGCTTTTTAAGGATTATTTTGTTGATGAGAGGAAGAAGTTA
GGAAGCAGGAAGAGCACGGACAGTAATAGCCTAAAATGTGCGATGGATCATATTTTGGAAGCCCAAGAGAAAGGAGAG
ATCAATGAGGACAATGTTCTTTACATTGTTGAGAACATTAACGTAGCAGCAATTGAGACAACATTATGGTCAATAGAATG
GGGCGTTGCAGAGCTAGTCAACCACCCTCGCATCCAGAACAAGCTTCGGAAGGAGCTCGACACCGTGCTGGGCTCCGGA
GTCCAAGTCACCGAGCCAGACATCGAAAAGCTGCCTTACCTCCAAGCTGTAGTCAAAGAGACTCTCCGCCTCCGAATGGC
GATTCCCTTATTGGTGCCCCACATGAACCTCCACGATGCAAAGCTAGGTGGGTACGACATCCCAGCGGAAAGCAAAATCT
TGGTGAACGCGTGGTGGCTCGCCAACAACCCCGACCACTGGAAGAACCCGGAGGAATTTAGGCCAGAGAGGTTTTTCGA
AGAGGAGTCTAATGTGGAGGCCAACGGGAATGACTTTAGGTACCTTCCTTTTGGTGTCGGTAGGAGGAGTTGCCCTGGG
ATTATTCTCGCTTTGCCTATTCTAGGCATTACTTTGGGTCGTTTGGTCCAGAATTTCGAACTCTTGCCTCCTCTAGGACAGTC
GAAGATTGATACTGCAGAGAAGGCTGGGCAATTCAGCTTGCAGATTATGAAGCACTCTACCATCGTTCTGAAACCGAGAT
CATTTTG

>VcC4.H2 K00487 augustus_masked-VaccDscaff33-processed-gene-307.8

ATGGATCTCCTCCTCTTGGAGAAAGCCCTGTTGGGCCTCTTCATAGCCATAGTCCTCGCCATCACCATTTCCAAG
CTCCGCGGCAAGCGTTTCAAGCTCCCGCCCGGACCCATACCTGTACCCGTATTCGGAAACTGGCTCCAAGTCGGCGACGA
CCTGAACCACCGCAACCTTACAGACCTAGCCAAGAAATACGGCGAAATCCTGTTGCTCCGGATGGGCCAACGCAACCTAG
TCGTGGTCTCCTCACCCGACTTGTCCAAAGAAGTGCTCCACACACAAGGGGTCGAGTTCGGGTCGAGAACCCGGAACGTC
GTTTTCGATATTTTCACGGGTAAGGGTCAGGACATGGTGTTCACCGTCTACGGCGAGCACTGGAGGAAGATGAGGCGTA
TCATGACCGTGCCGTTTTTCACGAACAAAGTGGTCCAGCAGTATCGGACCGGGTGGGAGTACGAGGCGGGTCGGGTTGT
GGAGGACGTGAAGAAGAACCCGGAGGCGGCGACGAATGGGATTGTTTTGAGGAGGCGGTTGCAGACGATGATGTATAA
CAACATGTACAGGATTATGTTCGATCGGAGGTTCGAGAGCGAGGATGATCCGTTGTTTGTGAAGCTCAAGGCGTTGAATG
GAGAGAGAAGCAGACTGGCTCAGAGCTTCGAGTATAATTATGGAGATTTTATTCCGATTCTGAGGCCGTTTCTGAGAGGG
TATTTGAAGATTTGCAGGGAGGTTAAGGAGAGGAGGTTGCAGCTGTTCAAGGATAATTTCGTCGATGAAAGAAAGAAGC
TCGCAAGCACCAAGAGGATGGACAACAACAGTCTGAAATGTGCCATGGATCATATTCTGGATGCACAGCAGAAGGGAGA
GATCAATGAAGACAACGTTCTTTACATTGTTGAGAACATTAACGTTGCCGCCATTGAAACAACACTATGGTCGGTAGAGTG
GGGGATAGCAGAGCTAGTGAACCACCCCAGAATCCAGACGAAGCTCCGGCACGAGCTCGATACCGTGTTGGGCCTGGGA
ACCCAAATCACAGAGCCTGACGTCCAGAAGCTGCCTTACCTCCAGGCCGTGATCAAAGAGACCCTCCGCCTCCGGATGGC
GATCCCACTTCTAGTCCCGCACATGAACCTCCACGATGCCAAGCTGGGCGGTTACGATATCCCCGCAGAGAGCAAAATCTT
GGTTAACGCATGGTGGCTCGCCAATAACCCCGAGACTTGGAAAAAACCAGAGGAGTTCAGGCCGGAGAGGTTCTTGGAA
GAGGAGGCTAAGGTCGAGGCCAACGGTAACGACTTCAGGTACCTCCCGTTCGGTGTTGGAAGGAGGAGTTGCCCAGGA
ATTATCCTTGCTCTGCCGATTCTTGGCATCACTATCGGGCGTTTGGTCCAGAATTTCGAGCTCCTGCCTCCGCCGGGGCAGT
CTAAGCTTGATACCTCAGAGAAGGGGGGACAGTTCAGCTTGCACATTTTGAAGCACTCCACCATTGTTCTGAAACCAAGAT
CCTTTTGA

>VcCAD1 K00083 maker-VaccDscaff3-augustus-gene-213.23

ATGAGTTCTGAAGTTACTCCAAAAGAAGACTGCCTTGGATATGCGGCAAGAGATCCTTCTGGCTTTTTATCACCT
TTTCAATTCAGCCGCAGGGCTATTGGAATTGATGACATTTCAATTAGTATCACACACTGCGGAGTTTGTTACGCCGAAGTC
GTGTGGACTCGGAATAAATTTGGAGATGCAAAATATCCCGTGGTGCCTGGGCATGAGATTGTTGGAGTTGTGAAAGAGG
TGGGATCAAACGTGAAAGGCTTCAAAGTTGGGGACCATGTTGGAGTTGGAACTTATGTGAACTCTTGCAGAGATTGTGA
GTATTGTAACGACGGGTTCGAAGTTTATTGCTCAAAGGGTGCTATCTTCACTTACAACCATGTTGACGTGGACGGTACCGT
CACTAGAGGAGGATTTTCCACTTACATTGTTGTTCATGAGAGGTACTGCTTCCAAATACCAAAAGATTACCCATTAGCATC
GGCAGCACCATTGCTATGTGCTGGGATTACTGTTTACTCCCCTATGATTCGTCACAAAATGAACCAACCTGGCAAATCACT
CGGTGTGATTGGGCTCGGAGGATTAGGGCACATGGCTGTGAAGTTTGGAAAGGCTTTTGGGCTGAATGTGACAGTATTC
AGCACCAGTATATCCAAGAAAGACGAGGCCCTGAATCTGCTTGGGGCTGACAAATTTGTCGTCTCGTCAAATGAACAAGA
GATGCTGGAAGTTTCAAAGTCATTGGACTTCATAATCGACACTGCATCAGGGACTCACCCATTTGATCCATACATGTCTCT
GTTGAAGACCAGTGGTGTCCTAGTCTTGGTGGGTGCCCCAACCGAAGTGAAGCTCAGCCCCGGAAGCCTCATTCAGGGC
ATGAAATCAATTTCAGGGAGTGCGGCAGGTGGTACAAAGGATACAAGAGAAATGCTAGACTTCTGTGCTAAAAACAAGA
TTTACCCAGAGATTGAAGTTATCCCAATTCAGTATGTGAATGAAGCTATTGAGAGGTTGATAAAAAGGGATATCAAGTAC
CGGTTCGTGATCGACATCGAGAACTCCTTGAAGTGA

>VcCAD2 K00083 maker-VaccDscaff22-augustus-gene-306.29

ATGAGTTCAGATCAAAGAAGGGAGGAGTGCCTCGGATGGGCAGCTAGAGACCCATCTGGATTTCTTTCCCCTT

ATAAGTTCGACCGAAGGGCTGTTGGAAGTGATGATGTATCAGTAAAAATCACACACTGTGGAGTCTGCTATGCTGATGTT
GCATGGACCAGGGATAAACTTGGAGGGTTTGGGGGATCCAAGTACCCCCTTGTGCCTGGGCATGAGATTGTTGGAATCG
TTAAAGAGGTGGGTTCCAATGCTCAAGGCTTCAAAGTTGGAGATCACGTTGGAGTGGGTACTTTGGTCAATTCATGCAAA
GATTGCGAGTACTGTAACATGTACATGGATGTATACTGCTCGAAGGGAGTTGTCCGTACTTTCAGCTCTGTTGATGTGGAT
GGTACAATCACAAAGGGTGGATATTCAACTTTCATTGTTGTTCATCAAAGGTACTGCTCCAGGATACCAAATGACTTTCCA
TTGCCCTTGGCAGCTCCACTCCTGTGCGCAGGAATCACCGTTTACAGTCCAATGATGCGCCACAAAATGAACCAACCGGG
AAAATCCCTGGGAGTGATTGGTCTTGGTGGCCTGGGTCACTTGGCAGTGAAATTCGGAAAGGCGTTCGGCTTGAGCGTA
ACTGTTTTCAGTACGAGTTTGTCCAAGAAAGATGAAGCCCTTAATAAGCTTGGAGCAGATGGATTCGTGATCTCTTCCGAC
GAAGAACAGATGAAGGCTATGGCGAAGTCACTCGACTTTATAATTGATACAGCATCAGGGGACCACCCATTTGATCCGTA
CATGTCGCTGTTGAAGACCGGCGGCACCCTTGTCTTGGTTGGTGTCCCAACTCAAGTCAAGCTCAGTCCTATCAGCCTAAT
AGGCGGCATGAAATCCATTACGGGTAGTGCAACGGGTGGCATCAAGGAGAGGCAAGAAATGGTGGATTTCTGTGCAGCT
CACAAGATATACCCAGAAATTGAGATCATTCCTATACAGTATATAAATGAAGCTTTGGAGAGGCTTATAAAGGGGGAGGT
GAAGTACAGGTTCGTTATCGATATCGAGAACTCCCTCAAGTGA

VcCAD3 K00083 maker-VaccDscaff49-snap-gene-1.40

ATGAGCTCCAATAGTACAAATGAGGACTGCCTTGGATGGGCGGCAAGAGACCCATCTGGAGTTTTATCGCCTTA
CAAATTCAGCCGCAGGCTTGTTGGACCTGATGATGTTTCACTAAGGATCACACACTGTGGAGTGTGCTATGCTGATGTTGC
CTGGACTAGAAATAAACAAGGACATTCTAAGTATCCCCTAGTACCCGGCCATGAAATTGTTGGGATTGTAAGGGAAGTTG
GTCCGAATGTCAAGCGCTTCAGTGTCGGTGACCATGTTGGAGTGGGGACCTATGTTAACTCATGCAGAGAGTGCGAGTAT
TGTAACGATGGTTTAGAAGTTCACTGCATGAAGGGAGCAATCTACACTTTTGACGGTTTTGACGTAGATGGTACCATCAC
GAAAGGAGGATACTCTAGTTTCATTGTTGTACATGAAAGGTACTGCTTCAGAATTCCAGAGAATTACCCATTAGCTTTGGC
AGCGCCTTTGCTATGTGCTGGAATTACTGTTTACACTCCCATGATGCGCCATAACATGAACCAACCTGGTAAGTCTTTGGG
TGTGATTGGGCTTGGTGGTCTTGGTCACTTGGCAGTGAAGTTTGGGAAAGCTTTCGGATTGAATGTTACGGTTTTTAGCAC
TAGTATATCAAAGAAAGAGGAGGCTCTGGTTCTCCTTGGAGCAGATAAATTTGTTCTCTCGTCTGACGAACAACAGATGCT
GGCCCTGGCTAAATCATTGGACTTCATTATCAACACAGCGTCTGGTGATATTCCATTTGATCCATACATGTCGCTGTTGAAG
ACTGCTGGTGTTTTGGTTTTGGTGGGGTTTCCAAGTGTAGTGAAATGCAGTCCAGCTAGCCTTATCCTGGGTATGAAATCC
ATTTCAGGAAGTATAACTGGAGGCACAAAACAAACTCAGGAAATGTTGGACTTCTGCGCTGCCCATAAAATTTTCCCGGA
GGTTGAAATAATTCCTATCCAGTATGCAAATGAAGCTCTTGAGAGGCTCATAAAGAAGGACGTGAAATATCGGTTCGTGA
TTGATATTGAGAACTCCCTTAAGTGACACCAGAAGGGGAATAGTGTTTTATCTATGCGTGCATGTGTCATTTACTTGAATT
GGAGCTACCTTGAAGCCTGTAGGTTTACATCTACTTTCTGCCAAAGATTATTCAGGAACCATGTTCTGTATCTTGTTACTAT
TTATGGTTATTAA

>VcCAD4 K00083 maker-VaccDscaff22-snap-gene-141.41

ATGGGTTCTGCAAAAGAAGACTGCCTTGGGTATGCGGCAAGAGATCCATCTGGGTTTTTATCACCTTACAAATT
CAGCCGCAGGACTCTCGGAAATGATGATGTTTCAATTAGTATTACAAATTGTGGAGTGTGTTACGCTGAAGTTTTGTTGAC
TAGGAATAAATTTGGGGATGCAAAGTATCCACTAGTGCCTGGACATGAGTTTGTTGGAGTGGTGAAAGAGGTGGGGTCG
GATGTGAAAGGCATTAAAGTCGGGGACCGTGTCGGAGTGGGAACTTACGTGAACTCGTGCAGAGATTGTGAGTATTGTG
GAGATGGAGTAGAAGTTTCTTGCTCAAAGGGGGTTGTCTTTACCTACAACCACGTTGATTACGATGGTACCATCACTAAA
GGAGGGTTTTCCACTTACGTTGTTGTTCATGAGAGGTACTGCTACAAAATACCAGAAGGTTACCCATCAGCATTAGCAGCA
CCATTGCTATGTGCTGGGATCACTGTTTACTCTCCAATGATTCGCCACAAAATGAACCAACCGGGAAAATCACTTGGTGTG
ATTGGGCTTGGGGGTCTTGGTCACTTGGCAGTGAAATTTGGGAAGGCTTTCGGCCTGAATGTGACTGTATTCAGCACAAG
CATTTCCAAGAAAGAGGAGGCCCTAAATCTGCTTGGAGCCGACAAATTTGTGATCTCAACTGACCCACAAGAGATGATGG
AAGTATCAAAATCATTGGACTTTATACTAGACACTGCATCAGGAACTCACCCATTTGATCCATACATGTCTCTGTTGAAGAC
TGGTGGTGTCCTAGTCTTGGTTGGGGCTCCAGGCGAAGTGAAGCTCAGTCCCGTGAGCCTTATTCACGGAATGAAATCAA
TTTCAGGGAGTGCAGCAGGTGGTACAAAAGATACAAGAGAAATGCTAGAGTTCTGCGCTGCAAACAAGATTTACCCAGA
GATTGAGATCATCCCAATTCAATATGTAAATGAAGCCATTGAGAGGCTAATTAAGAGCGATATCAAGTACCGGTTCGTGA
TCGACATCGAGAACTCCTTGAAGTGA

VcCAD5 K00083 maker-VaccDscaff149-snap-gene-2.68

ATGGCCCAAACAACTCCGAACCACACACAAACTGTGTCCGGCTGGGCAGCCCATGACCCCTCCGGCAAGATTAC
CCCTTACACATTCAAACGAAGGGAAAATGGGATCAACGATGTGACCATCAAAATCCTCTACTGCGGTATTTGCCATACTGA
TATCCACCATGTCAAGAACGACTGGGGTATCACCATGTACCCCGTTGTTCCCGGGCATGAAATTACTGGGTTGATCACCAA
GGTGGGAGGCAATGTGACCAACTTCAAGGTAGGGGACAGGGTTGGAGTTGGATGTTTGGCAGCGACGTGCTTGGAGTG
CGAATTTTGCAAGGAATCCCAGGAGAATTACTGTGACCAAGTCCAGTTTACTTACAATGGCATTTTCTGGGATGGTAGCAT

CACTTACGGTGGATACTCCAAGATGCTGGTTGCAGATCACAGATACGTCGTGCACATACCGGAAAACCTACCCATGGATG
CGGCGGCGCCTCTGTTGTGTGCCGGGATAACGGTGTACTGCCCACTGAAGGACAACAACTTGCTTGACTTACCAGGGAAA
AGGATTGGAGTGATTGGCTTGGGCGGACTGGGACATGTAGCTGTCAAATTTGGCAAGGCGTTCGGGCACCATGTGACGG
TGATTAGTACCTCTCCATCCAAAGAAAAAGAGGCTAGGGACCGGTTGGGCGCCGATGATTTTATCGTCAGCACTGATGCT
GCACAGATGAAGGCAGGGAAGAGGTCTCTGGACTTTATTTTGGACACCGTATCCGCGTACCACTCACTTGGGCCTTACTT
GGAACTGCTCAAAGTTAATGGAACATTAGTGATTGTGGGTGCACCAGACAAGCCCATGGACCTGCCTTCATTCCCTTTGAT
ATTTGGAAAGCGAGCGGTGAAAGGTAGTATGACAGGGAACATGAAGGAGACACAAGAGATGATGGACGTGTGTGGGA
AATACAACATTACGTGTGATATCGAGACGGTCATGCCTGAGGAAATTAATGAAGCCCTTGACCGGCTCGCAAAGAACGAT
GTCAAGTACCGTTTTGTGATCGATATTGCTAACCAGAAGCCATCGACTAGCATCTTCATCCCAACTCCGAAGCCTCTGATCT
G

>VccCoAOMT1 K00588 maker-VaccDscaff34-snap-gene-139.31

CTCTCTCCCCTCATATATACCCTCCTCGTTTCATCCGTCACCCCAGAGACTCGCAGGCAATCCACAGCGATCAAAA
AGAGTCCAAAGCAAGAAGGAAAGGAAAGAAATCAGAAAAATCAATTCGCAGTAATGGCAGCCACCGGAGAAACAGAGC
AGAAGCAGAACCTTAGGCACCAAGAGGTTGGCCACAAGAGCCTTTTACAGAGTGATGCTCTCTACCAGTATATACTTGAG
ACTAGTGTGTACCCAAGAGAGCCGGAATCGATGAAAGACCTGAGAGAGGTGACTGCGAAGCATCCATGGAACCTCATGA
CAACCTCCGCTGATGAAGGGCAGTTCTTGAACATGCTTTTGAAGCTGATCAACGCCAAGAACACCATGGAAATCGGTGTC
TACACCGGTTACTCTCTTCTCGCCACAGCCCTTGCCATCCCTGATGATGGAAAGATTTTGGCAATGGACATCAACCGCGAG
AACTACGAAATCGGTCTCCCCATAATTGAGAAGGCCGGCGTCGCCCACAAAATCGACTTCAGAGAAGGCCCTGCTCTCCC
TGCCCTAGATCAAATGATCGAAGATGGGAAGTACCATGGGACATTTGATTTCGTCTTCGTGGACGCGGACAAGGACAACT
ACATCAACTACCACAAGAGGCTGATCGATCTGGTGAAGATCGGGGGGCTGATCGGATACGACAACACCCTGTGGAACGG
ATCCGTGGTGGCCCCACCGGATGCGCCGATGAGGAAGTACGTTAGGTACTATAGAGACTTCGTGTTGGAGCTTAACAAG
GCCTTGGCCGCCGATCCCCGTATCGAGATCTGCATGCTCCCCGTCGGCGACGGGATTACCCTGTGCCGCCGCGTCAGCTG
A

>VccCoAOMT2 K00588 augustus_masked-VaccDscaff42-processed-gene-262.6

GGAACTAGCAACCCCTCCTGTCCTATAAAGCCTTTCCTTCTTTCAAAGTTTGTGAATACTCTCACGAGTTTATTTT
ATCGAACTCATCTGTGGAAGAGTGAACAGAATACAGTTGATCAGTTTGTCTATTCACAAACTCGCTGCCGTTGGAAAGCTA
TGGAACACCTAACATTGAAGGAAGGGACTCCAAGGAAGGGAATATTGCTGACTGAGGAGTTGCATCAGTATGTATTGGA
GACTAGTGTCTACCCTCGTGAACCAGAGCCCCTCAAGGAGATAAGAGATGCCACTGCAAGCGACCCATGGGCGTTTATGG
GTACTTCTCCTGATGGAGGTCAGTTAATCGCCATGCTTTTGAAGCTACTAAATGCCAAAAAGACCATTGAACTTGGAGTTT
TCACTGGATACTCACTTCTTCTCACTGCCCTTTCAATTCCAGATGATGGCAAGATTACGGCCATAGATGTGAATCGGGAGA
TGTATGAAATAGGGTTGCCAATTATTAAAAAAGCCGGTGTTGAGCACAAAATCGACTTTATTGAGTCTAAGGCTCTACCG
GTTCTTGATAAACTCTTGGAAGATAATGAGAATGAAGGGAGTTTCGACTTCGCTTTTGTTGATGCTGACAAGCCAAGTTAC
ATGGATTATCACAAGCGATTGATCAAACTAGTGAAGGTTGGCGGGATAATTGCATATGACAACACACTGTGGTATGGAAC
CGTAGCGATGCCAGAGGAAATGGTTCTAGAGGATCTGAGACCTAGTAGGGAGAGCACGATGAAGCTCAACAAAATGCTA
GCAGCTGATCCTCGTGTTATGATTGCTCATGCTTCAATAGGCGATGGAATCACGTTGTGTATGCGCGTCCAGTGA

>VcCCR1 K09753 maker-VaccDscaff4-augustus-gene-398.17

ATGCCATCAGCTTCCAGCCGAACCGTTTGCGTCACCGGCGCCGGTGGCTTCATCGCTTCATGGCTTGTCAAATTG
CTCTTGGAGAAAGGCTACATTGTCAGAGGCACCGTCAGAAATCCAGGCAAGAACTCGTATAACTACAAGGAGGAGAGAC
TAACTCTATGCAAAGCCGACCTTCTGGATTTCGAGAGCTTGGGCCGAGCAATTGATGGATGCGATGGTGTTTTCCACACTG
CTGCCCCTTTGTCTGATGACCCGGAAGAAGTGCTGGAGCCGATAATGATTGGAACAAAAAACGTGATAGTTGCGGCGGC
CGGAGCAAAGGTTCGGCGGGTGGTGTTCACATCAACAATTGGTGCTGTGACCATGGACCCTAACCGGAAGCCTGACACG
GTCGTGGACGAGAGTTGCTGGAGCGATCTTGAGTTTTGTAAGAACACTAAGAACTGGTATTGCTATGGGAAAGCAGTAG
CAGAGCAAGTGGCATGGGACGAGGCGAAGCAGAGAGGAATGGACCTCGTGGTAGTAAACCCAGTAGTAGTTACGGGAC
CATTGTTGCAACCCACTCTCAATGCCAGCACTCTCCACATTCTCAAATACCTAAATGGCTCGGCAAAAACTTATGCCAACTT
GGTCCAGGGCTACGTTCATGTCAAGGACGTGGCGTTGGCACACATTCTCGTTTTCGAGACTCCCTCCGCCTCTGGCCGGTA
CATTTGTGCTGAGAGCGTGCTTCACCGCGGCGATGTGGTCAAAATTCTTGCCAAATTGTTCCCTCAGTATCCCATTCCTACC
AAGTGCAAGGATGAAACAAATCCGAGAGCAAAACCATACAAGCTCTCAACTAAAAAACTTACGGACTTGGGCTTGGAGTT
CACACCCGTTGAGCAGTCACTGTACGAAGCAGTCAAGAGCCTTCAAGACAATGGTCAACTCCCAATACCTACTAAAAAAG
AGGATCATGCATCTTTTAGAGGAAGTAGCCTCTAA

>VcCCR2 K09753 maker-VaccDscaff8-snap-gene-149.28

ATGCCATCAGTTTCTGGCCAAACCGTCTGCGTCACCGGCGCCGGTGGCTTCATCGCTTCTTGGATGGTCAAATTG
CTCTTGGAGAAAGGCTACACTGTCCGAGGCACCGTCAGAAACCCAGGCAAGAATTCGTATATAAGGCAGGATGATCCGA
AGAATTCCCATTTGAGAAATCTGGAAGGAGCGGACGAGAGACTAACTCTATGCAAAGCCGACCTTCTGGATTTCGGGAGC
TTGCGCCAAGCAATTAATGGATGCGATGGTGTTTTCCACACCGCTTCCCCTGTTACTGATGACCCAGAAGAGATGGTGGA
GCCGGCAGTGATTGGAACAAAGAATGTGATAGTTGCGGCAGCCGAAGCAAAGGTTCGGCGCGTAGTGTTCACCTCATCG
ATCGGTGCAGTGACCATGGATCCTAACCGGGGGCCTGACACGGTTGTGGACGAGAGTTGCTGGAGTGATCTCGAGTTTT
GTAAGAACACCAAGAACTGGTATTGCTATGGGAAGGCAGTAGCAGAGCAAGCGGCATGGGACGAGGCAAAGGACAAA
GGAGTGGACCTAGTGGTAGTGACCCCAGTACTGGTAATGGGACCATTGTTGCAACCCACTCTCAACGCCAGCATTATCCA
CGTTCTGAAGTACCTAAATGGCTCGGCAAAAACTTATGCCAACTCGGTCCAGGCCTACGTGCACGTCAAGGACGTGGCGT
TGGCACACATTCTGGTCTACGAGACTCCCTCCGCCTCCGGCCGGTACCTTTGTGCTGAGAGCGTGCTTCACCGCGGAGATG
TGGTTGAAATTCTGGCCAAGTTCTTCCCTGAGTATCCCATTCCTATCAAGATTCTAGGATTCTTCCCTGGTCTTGTCGGAAA
CAAGTCTTTTGTTGCTTTTGTTGCCGGCCTTCTGGAGAGTCAAGTTTCCATTTTGATGTGCAAGGATGAAACGAAGCCAAG
AGCAAAACCGTACAAATTCTCAAACCAAAAGCTAAAGGACTTGGGTTTAGAGTTCACCCCGACGAAGCAGTCACTGTACG
AAACAGTCAAGAGCCTCCAAGACAAGGGTCACCTCCCAATACCCACTCAACAACAGGATCATGAATCTATTATTCGCATTC
ACTCTTGA

>VcCCR3 K09753 augustus_masked-VaccDscaff36-processed-gene-33.3

ATGCCATCAGTTTCTGGCCAAACCGTCTGCGTCACCGGCGCCGGTGGCTTCATCGCTTCTTGGATGGTCAAATTG
CTCTTGGAGAAAGGCTACACTGTCCGAGGCACCGTCAGAAACCCAGGCAAGAATTCGTATATAAGGCAGGATGATCCGA
AGAATTCCCATTTGAGAAATCTGGAAGGAGCGGACGAGAGACTAACTCTATGCAAAGCCGACCTTCTGGATTTCGGGAGC
TTGCGCCAAGCAATTAATGGATGCGATGGTGTTTTCCACACCGCTTCCCCTGTTACTGATGACCCAGAAGAGATGGTGGA
GCCGGCAGTGATTGGAACAAAGAATGTGATAGTTGCGGCAGCCGAAGCAAAGGTTCGGCGCGTAGTGTTCACCTCATCG
ATCGGTGCAGTGACCATGGATCCTAACCGGGGGCCTGACACGGTTGTGGACGAGAGTTGCTGGAGTGATCTCGAGTTTT
GTAAGAACACCAAGAACTGGTATTGCTATGGGAAGGCAGTAGCAGAGCAAGCGGCATGGGACGAGGCAAAGGACAAA
GGAGTGGACCTAGTGGTAGTGACCCCAGTACTGGTAATGGGACCATTGTTGCAACCCACTCTCAACGCCAGCATTATCCA
CGTTCTGAAGTACCTAAATGGCTCGGCAAAAACTTATGCCAACTCGGTCCAGGCCTACGTGCACGTCAAGGACGTGGCGT
TGGCACACATTCTGGTCTACGAGACTCCCTCCGCCTCCGGCCGGTACCTTTGTGCTGAGAGCGTGCTTCACCGCGGAGATG
TGGTTGAAATTCTGGCCAAGTTCTTCCCTGAGTATCCCATTCCTATCAAGATTCTAGGATTCTTCCCTGGTCTTGTCGGAAA
CAAGTCTTTTGTTGCTTTTGTTGCCGGCCTTCTGGAGAGTCAAGTTTCCATTTTGATGTGCAAGGATGAAACGAAGCCAAG
AGCAAAACCGTACAAATTCTCAAACCAAAAGCTAAAGGACTTGGGTTTAGAGTTCACCCCGACGAAGCAGTCACTGTACG
AAACAGTCAAGAGCCTCCAAGACAAGGGTCACCTCCCAATACCCACTCAACAACAGGATCATGAATCTATTATTCGCATTC
ACTCTTGA

>VcCCR4 K09753 maker-VaccDscaff27-snap-gene-293.30

ATGCCGTCAGTTTCCGGCCAAACAGTCTGTGTCACCGGCGCCGGTGGCTTCATAGCTTCATGGATTGTCAAATT
ACTCCTTGAAAAAGGCTACACTGTTAGAGGGACGGTCCGAAATCCTGATGATCCGAAGAACAGTCATTTGAGAGAAATG
GAAGGAGCAGAGGAGAGGTTAACTCTGTGCAGAGCTGATCTTCTTGATCTTCAGAGTTTACTGGAAGCCATTAATGGGTG
TGATGGTGTTTTCCACACAGCATCCCCTGTCACCGATGACCCTGAAGAAATGGTGGAGCCGGCGGTGAACGGAGCGAAG
ATCGTGATAGCCGCAGCGGCGGAAGCGAAAGTCAGGCGGGTCGTGTTCACGTCATCTATCGGTGCTGTGACCATGGACC
CTTACCGGAGCCCCGATACCATTGTTGATGAGAGTTGCTGGAGTGATCTTGAGTTCTGCAAGAACACCAAGAACTGGTAC
TGCTACGGTAAGGCGGTGGCGGAGCGGGCGGCATGTGAGGAGGCGAAGGAGAAAGGAGTGGACCTGGTGGTGGTGA
ACCCAGTACTGGTAATGGGACCATTGCTGCAGCCCACAGTGAATGCCAGTACCGTTCACATTCTCAAGTACCTGAATGGGT
CGGCCAAGACATACGCCAACTCGGTCCAGGCCTATGTGGACGTCAGGGACGTGGCCCTTGCCCACATTCTGGTCTACGAG
ACACCGGCCGCCTCCGGCCGGCATCTCTGCGCCGAGTGCGTGCTTCACCGCGGCGATGTGGTGAAGATTCTCGCCAAGTT
CTTCCCGGAGTATACCCTTCCCACCAAGTGCAAGGATGAAACAAAGCCAATGGTGAAACCATATCAAATCTCAAACCAAA
AGCTCAAGGACTTGGGTTTGGAATTCACACCAGTGAAGCAAAGCCTATACGACAGCGTCAAGAGCCTACAAGAGAAGGG
TCACCTCCCAATTCCAACACAACGTGATGAATCGACTAGTATTCGAAGTCGGTCCTAA

>VcCHI K01859 maker-VaccDscaff26-snap-gene-196.29

ATGTCTTCGCCGCCGGCCGTCACCGAAATCAAAATCGAAAGCCAAGTCTTTCCTCCGTCGGTCAAACCACCGGG
CACCACCAATTCCTTCTTCCTCGGTGGCGCAGGGGAGAGAGGTTTGGAGATTGAAGGCAAGTTCATAAAGTTCACGGCCA
TTGGAGTGTACCTACAAGTTAGCGCCGTAGAGTCGCTCGCCGTTAAGTGGAAGGGCAAGAGCGCGGAGGAGTTGACGG
AGTCCGTTGAGTTCTTCAGGGATATCGTATCAGGTCCCTTTGAGAAATTCACACAGGTGACAATGATCTTGCCTTTAACGG

GCAAGCAATACTCAGAAAAGGTGGCAGAAAATTGTGTTGCTTATTGGAAAGCAGTAGGAACCTATACTGATGCAGAGGC
CAAAGCGGTTGAAAAGTTTCTTGAGGTCTTCCAGGATGAGACATTCCCCCCTGGTGCTTCTATTCTTTTCACCCAGTCACCC
CATGGGTTGCTAACGATTGGCTTCTCGAAAGATGGTTTGTTACCTGAGACTGGCAAAGTGGTAATCGAGAACAAACAATT
GTCCGAAGCAGTGTTGGAATCAATCATCGGGAAGCATGGCGTTTCCCCTGCAGCAAAGCAGAGTTTGGCGGCAAGAGTG
TCTGCATTGTTCAAGGAGGCATAA

>VcCHS1 K00660 augustus_masked-VaccDscaff9-processed-gene-64.0

TTTTGTCACACATTCTCACTTAATCCTCCAATCAAATTTGGTAGTACCCTTCTAGCTACCACTCCCCAAGATAAAT
ATCCGGTTACCGGAAAACATTCTCCGGCGAAAAAAATGGTGACCGTCGAGGAAGTCAGGAAGGCGATGAGGGCCGAGG
GACCGGCCACGGTCTTGGCGATCGGGACGGCCACACCGGCCCATTGCGTCGAGCAGGCCACGTACCCCGATTACTACTTC
CGTGTTACTAACAGCGAGCACAAGGCCGAGTTGAAGGAGAAATTCCAGCGCATGTGTGACAAATCCCAGATCAAGAAGA
GGTACATGTACTTGACTGAGGAAATCTTGAAGGAGAACCCCAATGTGTGCGCGTACATGGCACCTTCCCTGGACGCTAGG
CAGGATATGGTGGTTGTGGAAATTCCCAAATTGGGCAAGGAGGCTGCTGTGAAGGCCATCAAGGAATGGGGACAGCCC
AAGTCCAAGATCACCCACTTGGTCTTTTGCACCACCTCCGGCGTCGACATGCCGGGAGCCGACTACCAGCTCACCAAGCTC
CTCGGGCTCCGTCCTTCCGTGAAGCGGCTCATGATGTACCAACAGGGTTGCTTCGCTGGTGGCACGGTCCTCCGCTTGGCC
AAGGACTTGGCAGAGAACAACAAGGGGGCTCGTGTTTTGGTGGTTTGCTCTGAGATCACCGCGGTCACCTTCCGTGGGCC
CAGTGACTCCCACCTCGACAGTCTTGTGGGCCAGGCCTTGTTCGGGGATGGTGCGGCCGCAATCATAGTTGGGGCCGACC
CGATCCCCGAGGTCGAGAAGCCCTTGTTTGAGGTGGTCTCCGCGGCCCAAACCATTCTCCCGGATAGTGATGGGGCCATC
GATGGACATCTCCGTGAAGTGGGCCTGACTTTCCATCTCCTCAAGGATGTTCCTGGGCTTATTTCAAAGAACATTGAAAAG
AGCCTAGTGGAGGCCTTCCAGCCTTTGGGCATTTCTGATTGGAATTCTATCTTCTGGATCGCCCACCCCGGTGGGCCTGCC
ATTTTGGACCAGGTGGAACAGAAGCTGGCCCTTAAGCCCGAGAAGCTACGGGCCACAAGGCACGTGCTGAGCGAGTACG
GTAACATGTCAAGCGCGTGTGTGTTGTTCATTATGGATGAGATGAGGAAGAAGTCGGCCGAAGATGGGTTCAAGACCAC
CGGTGAAGGGCTCGACTGGGGCGTGCTCTTTGGGTTCGGGCCTGGGCTTACCGTTGAGACCGTAGTGCTCCATAGTCTGT
GCACTTA

>VcCHS2 K00660 maker-VaccDscaff13-augustus-gene-46.32

ATGGCTCCTACAGGTGTGTCCGTGGAGGAAATCCGAAAGGCCCAGCGGGCACAGGGTCCGGCCACTGTCTTGG
CCATCGGGACCGCGACTCCGGCCAACTGCGTAAACCAGGCCGAATACCCCGATTTCTACTTTCGGATTACCAACAGCGAG
CACAAGACTGAGTTGAAAGAGAAATTCAAGCGAATGTGTGAGAAATCAATGATCAAGAAGCGTTACATGTACTTAACAG
AAGAAATCCTGAAAGAGAACCCAAGTGTGTGCGAGTACATGGCACCATCGCTGGACGCTCGGCAGGACATGGTTGTGGT
TGAGGTACCAAAACTGGGGAAAGAGGCTGCAACCAAAGCCATCAAAGAATGGGGCCAACCCAAGTCCAAGATCACCCAC
CTCGTCTTCTGCACCACTTCCGGCGTCGACATGCCCGGTGCCGATTACCAGCTCACCAAGCTCCTCGGCCTCCGACCTTCCG
TCAAGCGTCTCATGATGTACCAACAGGGCTGCTTCGCCGGCGGCACCGTCCTCCGCCTAGCCAAAGACCTCGCCGAGAAC
AACGCCGGCTCCCGCGTCCTCGTTGTTTGCTCCGAAATCACCGCCGTCACCTTCCGCGGCCCCTCCGACACCCACCTCGACT
CCCTCGTCGGCCAAGCCCTCTTCGGCGACGGAGCCGCAGCCGTCATCGTCGGCTCCGACCCGACACCACCCGAGCGCCCA
CTCTTCCAACTCGTCTCCGCATCACAAACGATCCTCCCGGACTCCGACGGCGCCATCGACGGCCACCTCCGCGAGGTGGG
GCTCACGTTCCACCTCCTGAAAGACGTCCCCGGTTTAATATCAAAGAACATCGAGAAGTCTCTGGTGGAAGCGTTTGCTCC
GGTCGGGATAAGCGACTGGAACGAGTTGTTTTGGATAGCTCACCCGGGTGGACCGGCGATTCTGGACCAGGTGGAGTTG
AAACTCGGGTTGAAGGAGGAGAAACTGAAGGCTACTCGACACGTGCTGAGCGAGTACGGGAACATGTCGAGCGCGTGC
GTGCTGTTTATTTTGGATGAGATGAGGAAGAAGTCTGTGGAGGAAGGGAGTGGGACGACGGGTGAAGGGTTGGAGTG
GGGTGTTTTGTTCGGGTTCGGGCCGGGTTTGACGGTGGAGACGGTTGTGCTCCATAGTTTGCCTGCACAGGGCGGTGTG
ACTCACTGA

VcCHS3 K00660 maker-VaccDscaff2-augustus-gene-67.14

ATGGTCACCGTCGAGGAAGTTCGCAAGGCACAACGGGCTTCAGGTCCGGCGACTGTCATGGCCATCGGCACCG
CTACTCCACCGAACTGCGTCGACCAGAGCACGTACCCGGATTTCTACTTTCGGATCACTAACAGCGAGCACAAGACAGAG
CTCAAAGAGAAGTTCCAGCGCATGTGCGACAAATCCATGATCAAGAAGCGTTACATGCACGTAAACGAAGAAATCTTAAA
GGAAAATCCCAGTATGTGTGAGTACATGGCACCTTCCTTGGACGCTAGGCAGGACATGGTTGTGGTGGAAGTCCCCAAAC
TCGGCAAAGAGGCTGCCACAAAGGCCATAAAGGAATGGGGCCAACCCAAATCCAAAATCACTCACTTGGTCTTCTGCACC
ACCAGCGGTGTCGACATGCCCGGCGCCGACTACCAGCTCACCAAGCTCCTCGGCCTCCGCCCGTCCGTCAAGCGTCTCAT
GATGTACCAACAGGGTTGCTTCGCTGGTGGCACGGTCCTCCGTTTGGCCAAGGACCTGGCAGAGAACAACAAGGGTGCT
CGGGTTCTAGTCGTTTGCTCTGAGATCACTGCTGTCACTTTCCGCGGGCCAAGTGACACGCACCTCGACAGTTTGGTTGGA
CAGGCCCTTTTCGGGGATGGCGCCGCCGCTATTATTGTGGGTGCAGACCCGATTCCCGAAGTTGAGAAGCCATTGTTTGA
GTTGGTCTCGGCAGCCCAAACCATTTTACCCGACAGCGAAGGGGCCATCGATGGACACCTTCGCGAAGTGGGCCTTACTT

TCCACCTTCTCAAGGACGTTCCTGGCCTGATCTCGAAGAACATCGAAAAGGCGCTCGCGGAGGCTTTTCAGCCATTGGGT
ATCTCTGACTGGAACTCAATTTTCTGGATTGCGCACCCTGGTGGGCCTGCCATTCTAGACCAAGTGGAACTGAAGTGTAAC
CTCAAGCCCGCGAAGCTACGGGCCACGAGGCACGTGCTGAGCGAGTACGGTAACATGTCGAGCGCGTGTGTGCTGTTCA
TTTTGGATGAGATGAGGAAGAAGTCGGCCGAAGAAGGGCTCAAGACCACCGGTGAGGGGCTCGAGTGGGGCGTGCTGT
TCGGGTTCGGGCCGGGGCTGACTGTTGAGACTGTGGTGCTCCACAGCTTGTGCACTTGA

>VcCL1   K01904   maker-VaccDscaff46-augustus-gene-86.25
ATGGAGAAATCTGGTTATGGGCGTGACGGTATATACAGATCGCTTCGCCCTCCTCTGCTTCTTCCCAAAGACCCA
AACCTCTCAATGGTCTCATTCCTCTTTCGAAACTCCTCCTCTTACTCCGACAAACCCGCCCTTATCGACGCCGACTCCGGCCA
AACCCTCACCTTCTCCCAATTCAAATCCACCGTCGCCAAACTCTCCCACGCCTTCATCGACCAACTGGGTATCAAGAAAAAC
GATGTCGTCCTCATCTTCGCCCCCAATTCAATCCAATTCCCCATCTGCTTCTTTGCAGTCGTCGCAATCGGGGCCATTGCTAC
AACTGTAAACCCTTTGTATACGGTAGCAGAGATTTCAAAGCAAGTCAAGGATTGTAAACCAAAGGTCATTGTTACCGTTTC
TGAGTTATGGGAAAAGGTGAAAGGGTTTGGATTGGATTATGTTATTATGGGTAATGAGAAAAATTCTAGCTTGATCGGTA
ACAGCTCCAATTCTAAGATTACATGGTTAATTGATTTGGTAAAAAATTCCGGGTCCACGACGGATTCGTTTCCCGTGGCTG
AAATTAGATCAAGCGATACGGCAGCTCTTTTGTACTCTTCAGGCACAACAGGGGTAAGTAAAGGAGTGGTTCTGACCCAT
AGGAATTTTGTGGCATCAGCTCTTATGGTTACTGCTGATCTAGAAATGGCGGGGGAGATGCACAACTTGTTATTATGTGT
GTTGCCTATGTTTCATGTTTTTGGACTTGCTGTGATTATGTGTGGGAATCTGCAGAGGGGGGATGGGATTGTGTCGATGG
CAAAGTTCGATTTTGAGATGCTTTTGGGGGCTGTGGAGAAGTATAGAGTGACACATTTATGGGTTGTGCCCCCTATTGTAC
TTGCTTTGGCTAAGAATAGTATGGTGAAGAAATATGATTTGTCGTCGTTGAGGCAAATTGGGTCAGGTGCAGCGCCATTG
GGGAAGGATTTGATGGCCGAATGCGCGAAGAATTTTCCTCAGGCTACAGTTTTGCAGGGCTATGGTATGACAGAAACGT
GTGGGATTGTTTCAGTTGAGAATCCAAGGTTAGGTCCTCGACATTCTGGTTCAGCAGGAATGCTTTCTCCAGGAGTGGAA
AGCAAAATAGTTGGTGTAGATACTCTGAAGCCTCTTCCTCCTAAACAATTGGGGGAAATATGGGTTCGTGGACCCAACAT
GATGCAAGGTTACTTCAACAATCCAGAGGCCACCAGACAGACTATAGATAAGCAGGGTTGGGTCCATACAGGAGATCTT
GGATACTTTGATGAGGAAGGACAATTATTTGTCGTTGACAGACTTAAAGAGCTCATCAAGTACAAAGGCTTTCAGGTTGC
ACCAGCAGAGCTCGAAGGGTTGCTTGTTTCTCACCCTGAAATACTGGATGCTGTTGTCATTCCGTTTCCCGATGCTGAAGC
TGGTGAGGTCCCGGTGGCATATGTTGTCCACTCACCCACCAGTTCTCTTACTGAAGGAGATGTGAAGAAATTCATTGCAG
ATCAGGTTGCACCTTTTAAAAGACTGCATCGGGTAACATTTATAAACAGTGTTCCAAAATCAGCTTCAGGAAAGATTCTGA
GAAGAGAACTCATTGAGAAAGTCCGGTCCAAGTTATGA

>VcCL2   K01904   maker-VaccDscaff47-augustus-gene-2.17
ATGGCAACAAGCATGAATGAGAATCTCAACTTGAACTCCCAAAAAACACAATCCACCTCTCGCCTTCACAACAAT
AACAAGTACTCCCACTGGTACTCCCCCGAAACAGGAATATACCAAAGCAAACACTCTCCAGTAGACCTCCCAAAAGACCCA
TTTCTCGACGTTGTTTCCCACATCTTCTCACAGCACCACCATGGTGTCTCCGCCCTCATTGATTCCACTTCTGGCCACACAAT
TCCCTACTCCCACCTCTACCCCTTAGTCAAATCCATGGCCTCTGGCCTCCACCAAATGGGTATTTCACAAGGTGATGTTGTT
TTGATCCTACTGCCTAATTCAGTCTACTTCCCTATCATTGTCTTTGGTGTCCTCTACTTGGGTGCTATTGTGACTACCATGAA
TCCTCTCAGTACTCTCTCTGAGATCAAGAAACAAGCACTTGATTCTAAAGCAATTCTTGCCTTTACTTTGCCTGATTGCCTTG
ACAAAATTTGCGGATTGGGTATTCCAGCAATAGGGGTGCCAGAAACTGGGGGTTCTGATTTAAAACAAATGGGTTTGTTA
AATTTTGATCAGTTAATTGGTGGGCAAAATCCCAATTCGGTTCCAAACCCAACGATTCATCAAGAAGACACTGCGGTGATT
TTGTATTCATCGGGCACAACCGGGACTAGTAAAGGGGTTGTGTTATCACATAGGAATTTAATATCTATGATTGAGCTTTTT
GTTAGGTTTGAAGCTTCCCAATATGAGTATTCGAGTGAGGAAAATGTGCATTTAGCCGTTATGCCCATGTTCCATATTTAC
GGGCTTTCGTATTTTGTGCTGGGGTTGTTGTCATTGGGTTCTACTATGGTTGTAATGAGAAAGTTTGATGCTAATGAAATG
GTGAAGGCTATTGAGAGATATAAGGTCACCCACTTCACTACCGTTCCGCCCTTACTGGCGGCACTGACGGAGAAAGCTAA
GAGCGTTGAGGGAAGTGTTTGGAAGAGTTTGAAGCAGGTTTGCTGTGGCGCAGCTCCAGTGACCAGGAAAAGCATAGA
GGATTTCGTACAGACTCTTCCTCATGTTGATTTCATTCAGGGTTATGGCATGACTGAGTCAACTGCAGTAGGAAGCCGTGG
CTTCAATACAGAAAAGAATCGTAAGTACACTTCGATGGGGCTTATGGCTCCAAATATGGAAGCTAGAGTGGTAGATTGGG
TTACCGGTTCCTTCATGCCTCCTGGGAGGAGTGGTGAACTTTGGTTTAGAGGACCTGCTGTTATGAAAGGTTACTTGAATA
ATGAAGAGGCAACTGCAATAACAGTGGATGAACATGGTTGGTTGCGTACGGGAGACATTGTATATTTTGACGAGGAAGG
GTACTTGCATATATTAGATCGCTTGAAAGAGATCATCAAATACAAGGGCTTTCAGATTGCTCCTGCTGAGTTGGAGGCTGT
ATTGGTTACCCATCCTGATATACTTGATGCAGCAGTAACAGGAGCCATGGATGGGGAAGCAGGGGAGGTGCCGATGGCT
TTTGTACTGAAGAGGCATGGAAGTCTGCTTTCTGAGGCAGCTGTATTAGACTATGTCGCTACGAAGGTTGCACCATACAA
GAAAGTGAGGAAGGTGGTGTTCACACAGTCAATACCTAGGTCGGCGGCTGGAAAGATTCTCCGACGGGAACTGAGGAG
CTTGATTTCTAGAATTTAA

>VcCL3   K01904   maker-VaccDscaff37-augustus-gene-303.53
ATGATGGAGCACCAAAACCATCAACGACAAGACGATCATCATCCTGAATTCATATTCCGATCAAAACTTCCCGAT

ATTTACATACCAAAACACTTACCACTCCACACCTACTGCTTTGAAAACATATCCCAATTCAGCTCCAAGCCCTGCATCATCA
ACGGCGCCACCGGTGAAACCCACACCTACGCCGACGTCGAACTCACCGCTAGAAGAGTCGCCTCCGGCCTCCACAAGATC
GGTATCCACCAGGGCGACGTCATCATGCTCCTCCTCCACAACTCTCCCGAATTCGTCTTCGCCTTCCTCGGCGCCTCGTACG
CCGGCGCCACCGCCACCACGGCCAACCCGTTTTACACCACGGCGGAGATCGAGAAGCAGGCGAGGGCGGCCAAGGCGA
AGCTGATCATCACCCAGGCGTGTTACGCCGAAAAAGTGACGGAATTTGGAAGGGAGAATGGGGTGAAGGTGATGTGCA
CGGACAAGGACGTGGAGGGGTGTTTGCATTTCTCTGAGTTGACTGGGGCAGACGAAAGCGAGTTGCCGGCGGTGAAGA
TTAGTCCGGATGATGCGGTAGCGCTGCCGTATTCGTCCGGGACCACTGGACTACCCAAGGGCGTTATGCTCACGCACAAG
GGGCTGGTCACTAGTGTGGCCCAACAGGTGGATGGAGAAAATCCCAACCTGTATTTCCACAGCGAGGACGTGATCGTCT
GTCTACTTCCACTATTCCACATCTACTCCCTCAACTCCGTATTGCTCTGTGGGCTCCGTGTTGGGGCCACGATCCTAATCAT
GCAAAAGTTTGAGATCAACGCGTTGTTGGAGCTCGTTCACAAGTACAAGGTAACGATAGCACCGTTCGTGCCACCCATCG
TTCTTGCAATAGCCAAAAGTCCGGTGGTCGATAACTACGACTTGTCGTCGATTCGGATGGTCATGTCCGGTGCTGCTCCCA
TGGGGAAGGAACTAGAGGATAAAGTCAGGGCTAAGCTTCCTAATGCTACACTTGGACAGGGTTATGGGATGACAGAGGC
AGGACCAGTGCTGTCAATGTGCTTGGCATTTGCAAAAGAACCGTTCGAGATAAAATCAGGGGCGTGTGGGACTGTAGTC
AGAAATGCCGAGATGAAAATCGTGGATCCCGACACCGGCAATTCTCTTCCCCACAATCAAGCCGGAGAAATCTGCATCAG
GGGCGACCAGATCATGAAAGGCTATCTAAATGATCCGGAGGCCACTGAGAGAACAATAGACAAAGAAGGATGGTTGCA
CACTGGCGATATCGGCTACATAGACGATGACGAGGAGATATTCATTGTTGATCGGCTCAAGGAACTGATCAAGTACAAAG
GGTTCCAAGTAGCCCCTGCCGAGCTTGAAGCATTGTTGATTGCTCATCCCAACATCTCAGACGTTGCTGTTGTACCCATGA
AAGATGAGGCAGCAGGAGAAGTTCCGGTTGCATTTGTTGTAAGAGCCAATGGTTCTAAGATCAGTGAGGATGAAATTAA
GCAGTATATCTCCAAACAGGTGGTATTCTACAAGAGAATTAACAAGGTGTTCTTCACCGATTCAATCCCTAAAGCTCCGTC
AGGTAAAATCCTCAGAAAAGACCTAAGAGCAAAGCTCGCAGCAGGTTTTCCCAATTAA

>VcCL4 K01904 augustus_masked-VaccDscaff50-processed-gene-21.6

TTTTTAAAAACCACCACCACCACCAACCCCCCCTACCACCTTCACCAACTCACATCCAATGCATCCATATATATCT
ATCTACTCATCAACATATCTATATCTACTCACTTCCATGAATTCAAACCCACATTAATCTCACCAACAATTGTACCCCATTATT
TCTCTCCAATTCCAACCACCTACAGTCCACACAGCTCAATCCCTGGACCAATGGAGATCACCAAACCACCACCACCACCACC
ACAAAAACAACAAGAAGATATTATATTCCGGTCAAAGCTCCCGGACATCTACATCCCCAACCACCTCCCCCTCCACTCCTAC
GTCTTTCAAAACATCTCCGACCACCGCTCGAACCCCTGCCTGATCAACGGCGCGACTGGTGAAATCCACACCTATGCCGAG
GTCGAGCTCACCGCCAGGAGAGTCGCCGTCGGCCTCAGCAAACACGGAATCAAACAGCACGACACGATCATGCTCTTGCT
CCCGAACTCGCCGGAATTCGTCTTCTCCTTCCTCGCGGCCTCCTACCTCGGCGCCACCAGCACCATGGCCAACCCCTTCTTC
ACGCCAGCGGAAGTCCTGAAACAAGCCAAAGCCTCCAAAGCGAAGCTCATCGTAACTCAAAACAGCTACGTCGACAAAGT
CCTGACCCACGCGCTCGAACACGACATCGGAATCGCGTGCATCGACGCGCTGCCGCCCTCCGCCGCGAAAAACGTTTTCC
ATTTCTCAGAACTGGCTCAATCCGACGAGAACGAATTGCCGGAAGTCGAAATCGATCCGAACGACGTCGTCGCTCTCCCG
TACTCGTCCGGGACTACTGGACTGCCGAAAGGAGTCATGTTGACACACAAGGGGCTCGTAACCAGCGTGTCGCAGCAAG
TGGACGGCGAGAATCCGCACCTGTACATGAACAGCGACGACGTCTCGATGTGCGTCCTGCCGCTGTTCCACATCTACGCG
ATGAATTCGATCCTGCTGTGCAGTCTGAGAGTCGGGGCGGCGATCCTTTTGGTGCAGAAGTACGAGATCTTGTCGTTTTT
GGGGTTGATGGAGAGGTATAAGGTGACGTACGGGCCGTTCGTGCCGCCGATTGTTTTGGCGATTGCGAAGAGTGAGAA
AGTGGACGATTACGATTTGTCGTCGGTGAGGATGGTGCAGTCCGGGGCGGCGCCGCTGGGGAAGGATGTGGAGGAGGC
TGTGAGCGCCAAGTTTCCGAATGCTACGCTTGGTCAGGGGTATGGAATGACAGAGGCAGGGCCAGTGCTGGCTTTGTGC
TTGGCATTTGCCAAGGAGCCATTTGAGGTTAAGTCAGGAGCTTGTGGGACTGTTGTGAGGAACGCCGAGATGAAGATCG
TCGACCCAGAAACAGAGAATTCTCTGCCTCGGAACCAACCGGGAGAGATTTGCATTCGAGGGGACCAAATCATGAAGGG
TTATCTAAATGATCCAGAGGCCACAGAGAGAACAATAGACAAAGGCGGCTGGTTACACACTGGCGACATAGGCTACATT
GATGACGACGATGAGCTATTCATCGTCGATCGTTTGAAGGAAATAATAAAATACAACGGGTTTCAAGTAGCCCCAGCAGA
AATTGAAGCCCTGCTGCTAAACCATTCAAACATCTCAGACGCAGCAGTTGTCGGGATGAAAGATGAATCAGCAGGAGAG
GTACCTGTTGCTTTTGTTGCGAAATCAAACGGGTCCCAATTAACCGAGGAAGAAATCAAGCAATACATCTCCAAACAGGT
AGTTTACTACAAGAGAATTAAAAGAGTTTTCTTCACCGATTCCATTCCGAAGGCACCGTCTGGCAAAATCTTGAGGAAGGA
CTTGCGAGCGAGATTAGCAGCTGGTCTTGCAAACTAG

>VcCL5 K01904 maker-VaccDscaff47-augustus-gene-69.25

ATGGAGAAATCTGGTTACGGCCAAGACGGCATATACAGATCACTTCGCCCTCCTCTGCTTCTACCCACAGACCCT
AACCTCTCAATGGTCTCGTTCCTTTTCAGAAACTCCTCCTCTTACTCCCACAAACCCGCCCTCATCGACGCCAACTCCGGCCA
AAGCCTCACCTTCTCCCAATTCAAATCCACTGTCGCCAAACTCTCCCACGCCTTCATTAACCAACTGGGTATCAAGAAAAAT
GACGTTGTCCTCATCTGCGCCCCAAATTCAATCCATTTCCCCATCTGCTTCTTAGCAACCATCGCAATCGGCGCTATTGCCTC
CACTGTAAACCCTGCATATACTGTATCAGAGATTTCAAAACAAGTCGAGGATTGTAAACCAAAGGTTATTGTAACAGTCCC

CGTATTATGGGATAAGGTTAAAGGGTTTGGCTTGAAATATGTTATGATCGGTAGTGAGGAAAAATCTAACTTGATTGGTT
ATAGCTTGAATTCGAAGGTTACATGGTTAAATGATTTGGTTAAGAATTCTGGGACGGTGTCGGATTCGTTCCCTGAGGCT
GAAATTAAATCGAGCGATACGGCTGCTCTGTTGTACTCTTCGGGCACAACAGGGCTAAGCAAAGGAGTGGTTTTGAGCCA
TAGGAATTTTGTGGCGGCGGCTCTAATGATTACCGCTGATCCAGAACTCGCGGGGGAGAGGGACAACTTGTTCTTATGTG
TGCTGCCGATGTATCATGTTTTCGGACTTTCTGTAATTATGTATGCGCAGCTCCAGAGGGGCGACGGGATCGTCTCGATGG
CGAAGTTCAATTTGAAGATGGTTTTGAAGGCAGTGGAGAAGTACAGGGTGACACATTTATGGATTGTGCCTCCAATTGTG
CTGGCTCTGGCTAAGAATAGTATAGTGAAGAACTATGATGTGTCGTCCCTTAAGCAAATTTTGTCCGCTGCAGCCCCTTTG
GTGAAGGACTTGATGGTGGAGTGTGCGAAGAATTTCCCTCACGCCGCAGTAATACAGGGCTTGGGTATGACAGAAACTT
GTGGGATTATTTCAAATGAGAATCCAAGGTTAGGACGCCGACATTCTGGTTCAGTAGGAATGCTTGCTCCAGGAGTGGAA
GGCCGAATAGTTGGTGTAGATACTCTGAAGCCTCTTCCTCCCAAACAGTCCGGGGAAATATGGGTTCGGGGAGCTAACAT
GATGCAAGGTTATTTGAACAATTCAGAGGCCACCAAATTTACTATAGATAAGGAAGGTTGGGTTCATACTGGAGATCTTG
GGTATTTTGATGAGGAAGGACAACTATTTATCGTTGACCGACTTAAAGAGCTCATCAAGTACAAAGGCTTTCAGATTGCAC
CAGCTGAACTCGAAGGGTTGCTTGTTTCACACCCCGAAATACTGGATGCTGGTGTTTTTGGGTTACCTGATGCTGAAGCTG
GTGAGATTCCGGTTGCAAATGTTGTCCGCTCTCCTACCAGTTCTCTTACAGAAAAAGATGTGCAGAAATTCATTGCAAATC
AGGTTGCACATTTTAAAAGACTGCATCAGGTAACATTTGTGGGAAGTATTCCAAAATCACCTGCAGGGAAGATTCTTAGA
AGAGAGCTCAAGGAGAAAGTTCGGTCTAAGATATGA

>VcCL6 K01904 maker-VaccDscaff48-augustus-gene-108.40

ATGTTAAAAGTGGCCTCTGCTGAAACCCAAAAACCAGACCTCTCTCCCCAATTACTAGGAGTAGGAATAGGAGT
AGGAGTAGGAGTAGATACTCCTCCTATTATCACTAATTCTACCCATAACCACCAATCCTCCTCCTCCTCCTCTGAAACCACC
ACCACCCTTACTACTACTAATAACTATGTTTTCAGATCAAAGCTTCCCGACATACCCATCTCCAACCACCTCCCTCTCCACAC
CTACTGTTTCGAGAATCTCTCCCATTTCTCCCACAATACCTGCCTCATTCTCGGCTCCACTAACAAAACCTACTCCTTCGCTG
AAACCCATCTCATTTCCCAAAAAGTCGGCGCCGGATTATCCAACCTTGGCGTTCGTAAAGGCCACGTAATCATGCTCTTACT
CCAAAACTGCGCCGAATTCGTCTTCTCTTTCATGGGTGCTTCCATGATCGGAGCAGTTACCACCACCGCCAACCCTTACTAC
ACTGCTGCTGAAGTCTTCAAGCAATTCCACGCATCCAAGGCCAAACTCATCATTACCCACGCCCAATACGTTGATAAGCTG
CGCCACGCAGGAGGAGGAGGTGATCATAATCTCCCAAAACTCGGCGAGGATTACACTCTGATTACGATCGATGACCCCCC
GGAGAACTGCCTTCACTTCTCTGTTCTGTCCGAGGCAAATGAAACCGATATCCCAACTGTTTCAATCGATCCAAACGACGC
CGTGGCGCTTCCTTTCTCATCGGGGACCACGGGTTTACCAAAGGGCGTGGTCTTGACTCACAAGAGTTTGATTACGAGTG
TGGCTCAACAAGTAGACGGAGACAACCCCAATTTGTTCTTGAAAACAGAGGATGTGGTGTTGTGCGTGCTTCCCCTGTTCC
ACATATTCTCGTTGAATTCGGTGCTTTTGTGTTCTTTAAGAGCAGGGTCCGGAGTGCTTCTGATGCAGAAATTTGAGATAG
GGACGCTGCTAGAGCTGATACAGAGGTACCGGGTGTCGGTGGCGGCAGTGGTGCCGCCGCTAGTTCTGGTGTTGGCGAA
GAATCCGATGGTGGAGAGCTTTGATTTGAGCTCCATCAGAACAGTGTTGTCAGGAGCGGCGCCGCTGGGTAAAGAGCTG
GAACAGGCTCTCCGGAGTAGAGTGCCCCGAGCCGTTTTTGGTCAGGGTTATGGGATGACGGAGGCAGGCCCAGTACTAT
CAATGAGCCTAGCATTTGCAAAGCAGCCTTTCCAAACAAAATCTGGGTCTTGTGGAACAGTAGTCCGGAATGCCGAGCTC
AAGGTCATTGACTCTGAAACCGGTTGCTCCCTTCCTCGCAACCAACAAGGCGAGATCTGCATTCGTGGTCTCCAAATTATG
AAAGGATATCTAAATGATGTTGATGCCACGGCTACCACCATTGATGTGGATGGTTGGCTACACACTGGGGACATCGGCTA
CGTAGATGATGACGATGAAGTTTTCATTGTTGATAGAGTTAAGGAGCTCATCAAATTCAAAGGCTTCCAGGCATGTTACTT
TATTTTAGTTCACCACTCGATCTTTTCCTTCTTTTCTTTTATTGTTATGTTTCAGTCAGACATTTGA

>VcCOMT1 K13066 maker-VaccDscaff20-augustus-gene-5.31

ATGGGGGGTTCAGAAAGCGAAAACCAAACAAGTAGTACTGCTCTAGTAGGATCAGAGGAGGAAGAAGAGGC
ATGTTTATTCGCTATGCAACTAGCCAGCGCCTCAGTCCTACCCATGGTGCTCAAATCAGCCATAGAGCTCGACCTCCTAGA
GACCATGGCGAAGGCCGGTCCCGGTGCCTTTGTTTCTCCCACCGAGCTCGCCGCCCAGCTCCCGACACAGAACCCGGACG
CGGCGGTGATGCTCGACCGGATTCTCCGACTCCTCACTAGCTACTCTGTTCTTAACTGTAGCGTGAAAAGCTTACCCGGCG
GAGGAGTGGAGAGGTTGTATGCCCTGGCGCCTGTTTGTAAGTATTTGACTAAGAATACTGATGGTGTATCCATGGCACCT
CTTTTGCTTATGAACCAAGACAAGGTCCTCATGGAAAGCTGGTACTATTTGAAAGACGCAGTTCTTGATGGTGGAATCCCC
TTCAACAAAGCTTATGGAATGACTGCATTTGAGTACCATGGTACAGACCCCAGATTCAACAAGGTCTTCAACCAGGGCAT
GTCCAATCACTCCACCATCACCATGAAGAAAATCCTTCAGATTTACCAAGGCTTTGAGGGCCTCACTTCGTTGGTTGACGT
CGGCGGCGGTACCGGGGCCACTCTTCACATGATTCGCTCCAAGTATCCCACCATCAAGGGCATCAACTTTGATTTGCCTCA
TGTTATCGAAGATGCTCCGACTTATCCCGGAGTGGAGCATGTTGGAGGTGATATGTTTGTTAGTGTACCAAAAGGAGATG
CCATTTTCATGAAGTGGATATGCCACGATTGGAGCGACGAACACTGCGCAAAATTTTTGAAAAATTGCTACGATGCATTAC
CAAACAATGGGAAGGTGATCGTTGCTGAATGCAATCTCCCTGTGGCCCCGGACACCAGCCTTGCCTCCAAGAATGTCATTC

ATATCGACGTTATCATGCTAGCTCACAACCCGGGTGGTAAAGAAAGGACCAAGGAGGAGTTTGAGGCCTTGGCGAAAGG
TGCTGGATTTGAAGGTTTCCGCGTCATGTGCAGCGCTTTCAATACCTATGTCATGGAATTCCTCAAGAAGATTTG

>VcCOMT2 K13066 maker-VaccDscaff20-snap-gene-377.34
ATGCAGACCTTTGACGAAGAGGAAAGCCATCGGCAGTATGCGATGCAGCTGGTGAGCGCATCGGTGCTGCCCA
TGAAATCAGCTATCGAGCTCGGGGTGCTCGAGATCATAGCAAAAGCTGGTCCGGGTGCTCTACTCTCCCCTTCTGAAATC
GCAACTCGCCTCCAGACCAACAACCCAGAAGCACCTCTGGTTCTAGATCGTATCCTTCGCCTTCTCGCTAGCTATTCCATTC
TCACTTGCTCCCTTTCTACACAGTGCAATGATGGCCAGCAGGAGGTCAATAGCAGTAGCAGTAGCAGTAGCAGCAGCAGG
CTCTATGGATTGGCACCTGTCTCCAAATACTTTATTCCCAACCAAGATGGTGTTTCACTTGCACCAATGTTACATGTAATTC
AGGACAAGGTTACCGTCAACATGTGGTACCATTTGACAGATGCTCTTATGGAAGGAGGGCTTCCAATCAATAGGGCCTAT
GGAATGAACAACTTTGATTATATTCTGGAGACATACAAAGACTTTAAAGGCCTGAATTTGATTGTGGATGTTGGCGGAGG
ACACGGTGCTGTTCTTAGTATAATTGTTTCCAAGTACCCTACAATCAAAGGCATAAACTTTGATCTCACTCCGGTTATAGAA
AAGGCACCTTCCTATCCTGGTATTGAATCAGATACTTATTTTGGACTGAGCATCCCCAAAGGAGACTCCATTTTCCTGAAAT
GGATACTTCACAGTTGCGATGACGAAAACTGCTTGAAGATACTGAAAAATTGCTACGATTCGCTACCAGATAATGTAAAA
GTGATTGTGGTGGATATGGTGATCCCAGAAACACCTGAAACCAGTGTTGACGTTAAAAGTGATTTTCAATTCGATTTGTTC
CTGCTGAACATGAACCCTGGAGGCAAGGAAAGGACAGAACGCGAATTTGAAAGTTTAGCAAAGGAAGCGGGGTTTTCAA
GGGTTGCTGCTTGTGCGTTCAAATTTTCACTTGTGGAGTTCTACAAAACTACATGA

>VcDFR1 K13082 augustus_masked-VaccDscaff13-processed-gene-166.8
ATGAAAGGTGTTAACAGTGGGCTGGGCACCACCGTGTGCGTCACCGGAGCCGCCGGGTTCATCGGGTCGTGGCTGATCA
TGAGGCTTCTTGAACGGGGCTATGTTGTTCGAGCAACCGTTCGCGATCCAGGCAATTTGAAGAAGGTGAAACACCTATTG
GAGTTGCCTAAAGCCGATACAAACCTAACGCTGTGGAAGGCGGACCTGAACGAGGAAGGAAGCTTCGACGAGGCCATTG
AAGGTTGCGTCGGAGTATTTCATGTCGCCACACCTATGGATTTTGAGTCCAAGGACCCTGAGAATGAGGTAATTAAGCCG
ACGATCAACGGTGTGCTAAGCATAATAAAGTCATGTACAAAAGCAAAGACAGTCAAGAGGCTGGTGTTCACATCTTCTGC
TGGAACCGTCAATGTTCAAGAGCACCAACAACCGGTGTATGATGAAAACAACTGGAGCGACTTGGATTTTGTCTATAAAA
TCAAGATGACTGGCTGGATGTACTTTGTGTCAAAAACACTAGCAGAGAAAGCAGCATGGGAAGCAGCCAAAGAAAACAA
CATTGATTTCATCAGTATTATACCAACATTAGTGGTAGGCCCCTTCATTATGCCTACATTCCCACCAAGCCTGATCACTGCA
CTCTCCCCTATCACAGGAAATGAACCTCATTACTCAATAATCAAGCAAGGCCAATTTGTGCACCTAGACGATCTTTGTGAAT
CTCATATATACTTATTTGAGCATCCTGAAGCAGAGGGGAGATACATTTGCTCATCCCATGATGCAACTATCTACGATTTGG
CGAAAATGATGAGGGAGAAATGGCCCGAGTACAATGTCCCCACTGAGTTTAAGGGGATTCCTAAGGATTTGCCAAATGT
GTCGTTTTCGTCGAAGAAGTTGATAGGGATGGGGTTTCAGTTCAAGTACAGCTTGGAGGATATGTTCAGAGGAGCCATTG
ATACTTGTAGGGAGAAGGGATTGCTTCCTTACTCTAATGAAACAACTGCCAATGGAAATGGAAATGGAACGATTTAG

>VcDFR2 K13082 maker-VaccDscaff12-snap-gene-66.28
TGGTTATAAATTCTGTATATAACCAAGACACAAACACGTACACAATAACATTCCAGGAGAAGAAGGACATGGA
AGGAGAGAACAGAGGAAGTAGAACCACCGTGTGCGTGACGGGGGCGTCGGGATTTATCGGGTCATGGCTTGTCATGAG
GCTTCTCGAACGAGGCTATTTTGTTAGAGCCACAGTACGCGACCCTGCTAATGCGAACAAGGTTAAGCATCTTTTAGACTT
GCCCAAAGCTGAAACACACCTGAGCTTGTGGAAGGCAGATTTAATGGAGGAGGGAAGTTTTGATGATGCCATTCAAGGT
TGTGCTGGTGTCTTTCATGTTGCTACCCCTATGGAACTTTCCTACAATGATCTTGAGAATGAAGTGATTAAACCGACCGTAA
ATGGGGTTTTAAACATCATGAGATCATGCTGCAAGGCCAAAACCGTCAAGAGACTCGTTTACACTTCAACCACAGGAACC
GTTGCCGTCCAAGAACACCCCCAACCTGAGTACAATGAGAGTTTCTGGACCGATATAGACTTCTGCAAAGCCCAAAAGAT
GACAGCATGGATGTATTTCGTGGCGAAAACAATGGCCGAGAAAGCAGCATGGGAATTTGCTAAAGAGAATGGTCTTGAT
CTTGTAACCATTCACCCTTCTGTTGTTATCGGGCCATTTATAACTCCGTCAAGACCTCTCAGCGTCGATCTCTCACTTTCCGT
AATAACGAGAAATGAAGCTCTTTACCCGATGGTAACTCGAGCTTGGGCCGTTCACGTGGATGATGTATGCAATGCTCATA
TATACCTTTTTGAGAATCCGAAAGCCGAAGGAAGATATATTTGCTCGTCTCACCGTTTTACCATTCTAGATCTTGCCAAATC
GTTGAGCCGACGATACCCCGAGTACAATATACCGATCAAGTTTGATGGTGTTGATGAATTATTGAAACCTGTCCCTTGCTC
GTCTAAGAAACTCATGGACCTAGGATTCAAATTCAAGTACAGCCCAGAAGAGCATGATGTCGGGGATTTGGTCGCGGAA
GCAATTGAATCATGCAGAGAAAAGGGGCTGATGCCAGACATGGAAGGAGAGAACAGAGGAAGTAGAACCACCGTGTGC
GTGACGGGGGCGTCGGGATTTATCGGGTCATGGCTTGTCATGAGGCTTCTCGAACGAGGCTATTTTGTTAGAGCCACAGT
ACGCGACCCCGCTAATGCGAACAAGGTTAAGCATCTTTTAGACTTGCCCAAAGCTGAAACACACCTGAGCTTGTGGAAAG
CAGATTTAATGGAGGAGGGGAGTTTTGATGATGCCATTCAAGGTTGTGCTGGTGTCTTTCATGTTGCTACCCCTATGGAAC
TTTCCTTCAATGATCTTGAGAATGAAGTGATTAAACCGACCGTAAATGGGGTTTTAAACATCATGAGATCATGCTGCAAGG
CCAAAACCGTCAAGAGACTCGTTTACACTTCAACCACAGGAACCGTTGCCGTCCAAGAACATCCCCAACCTGAGTACAATG
AGAGTTTCTGGACCGATATAGACTTCTGCAAGACCCAAAAAATGACTGCATGGATGTATTTCGTGGCGAAAACAATGGCT

GAGAAAGCAGCATGGGAATTTGCTAAAGAGAATGGTCTTGATCTTGTAACCATTCACCCTTCTGTTGTTATCGGGCCATTT
ATAACTCCGTCGAGACCTCTCAGCGTCGATATCTCACTTTCCGTAATAACGAGAAATGAAGCTCTTTACCCGATGGTAACTC
GAGCTTGGGCCGTTCACGTGGATGATGTATGCCATGCTCATATATACCTTTTTGAGAATCCGAAAGCTGAAGGAAGATAT
ATTTGCTCGTCTCACCGTTTTACCATTCTAGATCTTGCCAAATTGTTGAGCCGACGATACCCCGAGTACAATATACCGATCA
AGTTTGATGGTGTTGATGAATTATTGAAACCTGTCCCTTGCACGTCTAAGAAACTGATGGACCTAGGATTCAAATTCAAGT
ACAGCCCAGAAGAGCATGATGTCGGAGATTTGGTCGCGGAAGCAATTGAATCATGCAGAGAAAAGGGGCTGATGCCAG
TGTAG

>VcF3.5.H1 K13083 maker-VaccDscaff29-augustus-gene-305.28

ATGAGTGTCACTACTCTTGATCTCCTCTCTGCAATTTTCCTCTTTTTGATTACTCATTTTTCCATAAAGTATTTTCTG
TCCAGGAGGCTCCGTTACCCGATGTTGCCGCCCGGGCCTGGCGGCTGGCCGGTGGTGGGTTGCTTGCCGCTCTTAGGGAC
CATGCCGCATGTCGCACTTGCTCAAATGGCCAGAAAATATGGCCCCGTCATGTACCTTAAAATGGGCACTTGTGACATGGT
GGTTGCATCGAGCCCTGGGGCGGCTAGTACATTCCTCAAGACTTTGGACCACAATTTCTCTAACCGCCCTCCTGGTGCCGG
TGCGACCCATATTGCCTACGGTGCACAAGATTTTGTTTTCGCGGATATTGGGCCACGGTGGAATCTGTTTAGGAAATTGAG
TAATTTGCATATGCTTGGAACCCAATCGTACAAAGATTGGGCCCAGATAAGAGTAAAAGAACTTGGTCATGTGATTCAGG
ATATGTTTCAATTGAGCCGAAAGGGTGAACCGGTGGTGGTGCCCGAAATGTTGTTATGTGCCATGGCCAACCTAATAGGG
CAAAAGAGTCTTAGCCGCCGCGTGTTTGCTACGCTAGGCTCGGAGTCTAATGAGTTCAAGTACATGGTTGTGGAGCTCAT
GAGATTAGCTGGATTATTCAATGTTGGTGATTTCATACCATCAATCGCGTGGATGGATTTACAAGGGATCGAGCGGAAAA
TGAAGCGCCTACACAATCAATTTGATGGTCTACTAACAAAAATGATCGAGGAACACTCGTCAACAGCCCACGAACGCGAG
GGAAATCCAGACTTTCTCGACATCGTTATGGCCAACCGGGAGGTTCCCGACGGGCCGGAGCTCACAATGACCAATATTAA
AGCCCTTCTTTTGAATTGGTTCATTGCCGGCACCGATACATCAGCCGGCACAGTGGAATGGGCACTAGCAGAGATGATCA
AGAACCCAAGAATTCTCGAACGGGCACAAAAAGAACTGGACCGGGTAATCGGCAGAAACCGTCGTTTAGAAGAATCTGA
CTTGCCTAATCTTCCATACTTGCAAGCCATATGCAAGGAAACACTTAGGTTGCACCCACCAGTTCCCCTGAGTATTCCTAGA
GTATCCGTTGATCCATGTGAGGTCAACGGTTACTACATTCCTAAAAACACTAGGCTTTTCGTCAACATATGGGCCATCGGG
CGAGACCCGAATGTGTGGGAGGATCCAATGGAGTTCAACCCGGATAGGTTTTTAAGGGAGAAGATTAATGGGAAGATTG
AACCATGGGGGAATGATTTTGAGCTCATCCCATTTGGTGCTGGGAGAAGAATTTGTGCAGGAATTAGGATGGGAATTGC
AGCTGTTGAGTACATTTTGGGCACCCTTGTGCACTCTTTTGACTGGAGGCTTCCGGAGGGGGCGGCGCTCGACATGGGCG
AGGCGTTTGGCCTGGTGTTGCAAAAGGCGGTCCCACTGTCGGCGGTGGTCGTTCCAAGGTTACCGACAAGTGCTTATGAG
TCCTAA

>VcF3.5.H2 K13083 maker-VaccDscaff19-augustus-gene-162.21

ATGGCTGTGGACACATTCCTCTTGAGGGAGATTGCTACAGCAACCCTAGTCTTTCTTCTCACTAGGCTCTTCCTT
GGTTCCCTCCTCCTCCTCCTCCTCAAACCTGCTCGTAAACTCCCGCCCGGTCCTAAAGGGTGGCCGATCATTGGCGCCCTAC
CCCTTCTTGGAACCATGCCTCATGTTACCCTATCCCAAATGGCCAAAAAATATGGACCCATCGTCTACCTAAAAATGGGAA
CTTGTGACATGGTGGTAGCCTCCACTCCTGATTCGGCTCGAGCATTCCTAAAAACCCTAGACATGAATTTCTCAAACCGAC
CACCAAATTCTGGTTCAACCCACATGGCCTATAATGCCCAAGATTTGGTTTGGGCTGACTACGGCCCGAGATGGAAGTCG
TTACGAAAGTTATGCAACCTACAGATGCTTGGTGCGAAAGCGCTTGATGATTCGGCTCATATTCGAGCTACGGAGATAGG
GCACATGGTTCAAGCCATGTGTCAGTCTAGCCAGAGAGGCGAGGCTGTGGTGATACCGGAGATGTTAACTTTTGCTATGG
CCAACATCATAGGCCAAGTAATACTTGGGCGTCGCGTGTTTGCCCAAAAGGGTGAGGAGTCGAATGAGTTTAAGGACAT
GGTGGTGGAGCTCATGACCTCGGCTGGATTATTCAACATGGGCGATTATATACCTGCGATAGCGTGGATGGACTTACAGG
GGATTGTACGAAAGATGAAGCGTACAATTAACAAGTGGGATGTTATAATAAAGAAGATGGTAACAGAGCATACGGAGCT
GGCTCGTCAACGCGGAGGGAACCCAGATTTTCTTGATGTTCTTATGAGTAATAGAGAAAATCCTCAAGGCCCCGAGCTTA
GCATGACCAACATCAAAGCACTCCTTTTGGATCTATTTACCGCTGGAACTGATACATCTTCCAGCGTAATCGAATGGGCAC
TAGCTGAAATGTTACTGAATTCAGACATCCTAAAACGGGCTCAAGAAGAGATGGATCAAGTCATTGGTAGAAACAGAAG
ATTGCAAGCGTCTGATATTCCAAAGTTGCCTTATCTACAGGCCCTATGCAAAGAAACCTTCCGAAAGCACCCTTCTACCCCT
CTAAACCTCCCACGAATGGCATCCGAAGCATGTGAAGTTAACGGCTACTACATACCAAAGAACACGAGACTAAGCGTGAA
CATATGGGCGATCGGGAGAGATCCGGACGTTTGGGAAAACCCTTTGGAGTTCAACCCGGAAAGATTTCTGAGCGGAAAA
AACGCAAAAATCGATCCACGGGGAAACGATTTCGAGCTGATTCCGTTTGGGGCGGGAAGGAGGATTTGTGCGGGAGCA
AGGATGGGAGTTGTGATGGTTGAGTACTTTTTAGGGACATTGGTTCACTCGTTTGATTGGAAATTGCCTGATGGGCTGGT
TGAGCTAAACATGGATGAGACTTTTGGTCTTGCTTTACAAAAGACTGTGCCTCTCGCAGCTATGGTTACCCCGCGGCTACA
CCAAAATGCCTATGCCACTTAA

>VcF3.5.H3 K13083 maker-VaccDscaff10-augustus-gene-348.25

ATGGCCCTAGACATAATGTTGTTCAGGGAGATTGCAGCAGCAACTGTAGTCTTTCTGCTCACTAGGCTCTTCCTT

GGTTCCATCCTCCAGCTCCTCAAACCCACCTGTAAACTCCCACCCGGTCCGAAAGGATGGCCGGTCGTCGGCGCCTTACCA
CTTCTTGGAACCATGCCTCATGTTGCCCTAGCCAAAATGGCCAAAAAATATGGACCCATCGTCTACCTAAAAATGGGCACT
TGTGGCATGGTGGTAGCCTCGACTCCCGAGTCGGCTAGAGCCTTCCTCAAAACCCTTGACATGAATTTCTCAAACCGGCCA
CCTAATGCCGGTGCAACCCACTTGGCCTACAACAGCCAAGACATGGTTTTTGCCGACTACGGCCCGAAATGGACGTTGTTA
CGCAAGTTGTGCAACGTACACATGCTTGGTGCAAAAGCGCTGGATGATTCGGCTCATATCCGGAAATCGGAGTTGGGGC
ACATGCTCCAAGCCATGGTCGAATCGAGCCAACGAGCCGAGCCAGTGGTGATATCGGAAATGATGACTTACGCCATGGC
GAACATGATAGGGCAAGTCATACTCAAGCGTCGCGTGTTTGTCCAAAAGGGATCGGAGTCAAACGAGTTCAAGGACATG
GTGGTGGAGCTCATGACCTCGGCTGGACTATTTAACGTGGGCGATTTTATACCGTCGGTCGCGTGGATGGACTTACAAGG
GATCGAGGGTGGGATGAAGCGTATGCATAGCAAGTGGGATGATTTGATAACGAGGATGGTGAAGGGACACAGTGAGTC
GGCTCATGAGCGTAAAGGGAACCCGGATTTTCTTGATGTTCTTATGGCTAATAAAGAAAATTCTCAAGGCACCGGATCACT
TAGCCTCACCAACATTAAAGCACTCCTTTTGAATCTGTTCACTGCCGGCACCGATACATCATCTAGCGTAATTGAATGGGCA
CTAGCTGAAATGGTACTGAATCCTGATATCCTAAAACGGGCACAACAAGAGATGGATCAAGTCATTGGAAGAAACAGAA
GATTACAAGAATCTGATATTCCAAAACTACCTTATCTACAAGCCTTATGCAAAGAAGCCTTTCGACTGCACCCTTCCACCCC
ACTTAACCTTCCACGAATCTCATCCGAAGGATGCGAAGTGAATGGCTACTATGTACCAAAGAATACAAGGCTAAGCGTGA
ACATATGGGCGATTGGGAGAGATCCTGACGTCTGGGAAAACCCTCTGGAGTTCAACCCGGAAAGGTTTATGAGCGGAAA
AAACGCGAAAATGGATCCAAGAGGGAACGATTTCGAGTTGATTCCGTTCGGGGCGGGTAGGAGGATATGTGCAGGAGC
TAGGATGGGGGTTGTGATGGTTGAGTACTTTTTGGGCACATTGGTGCACTCATTTGACTGGAAATTGCCAGATGGGGTGG
CTGAGCTAAATATGGATGAGACTTTTGGTCTGGCTTTGCAAAAGGCCGTGCCTCTGGCTGCCAAGGTTACCCCACGGCTG
CATCCAAGTGCTTATGCTATGTAG

>VcF3.5.H4 K13083 maker-VaccDscaff24-augustus-gene-336.30

ATGGCTGTGGACACATTCCTCTTGAGGGAGATTGCTACAGCAACCCTAGTCTTTCTTCTCACTAGGCTCTTCCTT
GGTTCCCTCCTCCTCCTCAAACCCGCTCGTAAACTCCCGCCCGGTCCTAAAGGGTGGCCGGTCATCGGCGCCCTACCCCTTC
TTGGAACCATGCCTCATGTTGCCCTGTCCCAAATGGCCAAAAAATATGGACCCATTGTCTACCTAAAAATGGGAACTTGTG
ACATGGTGGTAGCCTCCACTCCTGATTCGGCTCGAGCATTCCTAAAAACCCTAGACATGAATTTCTCAAACCGACCACCAA
ATTCTGGTTCAACCCACATGGCCTATAATGCCCAAGATTTGGTTTGGGCTGGCTACGGCCCGAGATGGAAGTCGTTACGA
AAGTTATGCAACCTACACATGCTTGGTACGAAAGCGCTTGATGATTCGGCTCATATTCGAGCTACGGAGACAGGACACAT
GATTCTAGCCATGTGTGAATCGAGCCAGAGAGGCGAGGCTGTGGTGATATCGGAGATGTTAACTTTTGCAATGGCCAAC
ATCATAGGCCAAGTGATACTTGGGCGTCGTGTGTTTGCCCAAAAGGGTGCGGAGTCAAATGAGTTCAAGGACATGGTGG
TGGAGCTCATGACCTCGGCCGGATTATTCAACGTGGGCGATTATATACCTACGATAGCGTGGATGGACTTACAAGGGATT
GTACGAAAGATGAAGCGTACAATTAACAAGTGGGATGTTATAATAAAGAAGATGGTAACAGAGCACACGGAGCTGGCTC
GTCAACGCGAAGGGAACCCAGATTTTCTTGATGTTCTTATGAGTAATAGAGAAAATCCTCAAGGCCCCGAGCTTAGCATG
ACCAACATCAAAGCACTCCTTTTGGATCTATTTACCGCTGGAACTGATACATCTTCCAGCGTAATCGAATGGGCACTAGCT
GAAATGTTACTGAATTCAGACATCCTAAAACGGGCACAAGAAGAGATGGATCAAGTCATTGGTAGAAACAGAAGATTGC
AAGCGTCTGATATTCCAAAGTTGCCTTATCTACAGGCCCTATGCAAAGAAACCTTCCGAAAGCACCCTTCTACCCCTCTAAA
CCTCCCGCGAATGGCATCCGAAGCATGTGAAGTTAACGGCTACTACATACCAAAGAACACGAGACTAAGTGTGAACATAT
GGGCAATTGGGAGAGATCCAGATGTTTGGGAAAACCCTTTGGAGTTCAACCCAGAAAGGTTTCTGAGCGAAAAAAACGC
AAAAATGGATCCACGGGGAAACGATTTCGAGCTGATTCCGTTTGGGGCGGGAAGGAGGATTTGTGCGGGAGCAAGGAT
GGGAGTTGTGATGGTTGAGTACTTTTTAGGGACATTGGTTCACTCATTTGATTGGAAATTGCCTGATGGGATGGTTGAGC
TTAACATGGATGAGACTTTTGGTCTTGCTTTACAAAAGACTGTACCTCTCGCAGCTATGGTTACCCCACGGCTACACCAAA
ATGCCTATGCTACTTAG

>VcF3.H K05280 maker-VaccDscaff32-augustus-gene-159.26

ATGCCCTCACTCTTAAGAAAAATATTTGTCACTAATCACACAGGCCAAACCATGGCTTCTCTGGCTTTCATCATCT
ACTCTCTCCTCATCGGCATCTTCCTATACTTTGTTCTCAGCCTCCTCCGCAACCGGTACCCGCGGCCGCTCCCGCCAGGGCC
GAAGCCATGGCCAGTGATCGGCAACCTGCCCCACCTGGGCACGATGCCGCACCACTCCATCGCGGCCCTGGCTCGGACGT
ACGGGCCGCTGATGCACCTCTGGATGGGGTCGGTGCACGTGGTGGTGGCGGCGTCGGCGTCGGTGGCGGCGCAGTTTTT
GAAGACGCACGACCAGAATTTCTCGAGCCGGCCGCCGAACTCCGGGGCGAAGCACATAGCTTATAACTATCAGGACCTTG
TGTTTGCGCCGTACGGGCCGCGGTGGCGGATGCTTAGGAAGATATGCTCGGTTCACTTGTTCTCTGCTAAGGCCTTGGAT
GACTTCCGCCACGTCCGCCAGCTGTTCTTAATACCGCAGGAGGAGGTAGCGATCCTCACGCGCGCGTTAGCGAGTGCGGC
CAAATCCAAAACAACCGTAAATCTCGGCCAGCTCCTGAACCTGTGCACCACCAACGCCATCGGGCGCGTGATGCTGGGGC
GGCGCGTGTTCGGCGACGGCAGCGGCGGTGGAGACCCGAAGGCCGACGAGTTCAAGGGGATGGTGGTGGAGCTGATG
GTCCTCGCCGGAGTTTTCAACATCGGCGACTTCGTGCCGTCGCTGGAGTGGCTCGATCTGCAAGGAGTCGCCGGGAAAAT

GAAGAAGCTGCACTCGAGATTCGATGCGTTTCTGAGTGAGATTCTCGAGGAGCATAAGGTGGGGTCCATTGGTGGTGGG
GCCCAGAGTCACACTGATTTGTTGAGCACTTTGATTTCGCTCAAGGAGGAGGATGATGGCGAGGGAGGGAAGCTCACCG
ATACCGAAATCAAAGCCTTGCTCTTGGATTTGTTCACAGCAGGTACTGACACCTCATCCAGCACAGTAGAGTGGGCCATAG
CGGAACTTCTCCGACACCCGAAAGTCCTAGCTGAAGCCCAAAGAGAGCTGGACTCAGTTGTTGGGCCTGATCGGCTAGTA
ATGGAGGCTGACTTAGCCCAATTAACCTACCTCCAAGCTGTTATCAAGGAAACCTTCCGGCTCCACCCATCAACCCCACTTT
CCCTCCCACGGATGGCGGCCGAAAGCTGCGAAATCAACGGCTACTTCATTCCAAAGGGTTCGACTCTCCTCGTCAACGTGT
GGGCGATAGCCCGTGATCCAGAAGCTTGGGACAATCCTCTGGAGTTCAAGCCCGAACGGTTCTTACCCGGTGGGGAAAG
GCCCAATGCTGATATCCGAGGGAATGATTTTGAGGTCATTCCATTTGGGGCGGGCCGAAGGATATGTGCCGGGATGAGC
CTTGGGCTTCGGATGGTCCAGCTACTAACCGCGACATTGGTCCATTCCTTCAACTGGGAATTGCCTGAGGGAAAATTGGC
CGAGAAGCTTAACATGGATGAGGCATATGGGCTGACCTTACAGCGGGCCGAACCCCTCATGGTGCATCCAAGGCCAAGG
TTTGGGGCCCATGTGTAA

>VcF3H1K00475 maker-VaccDscaff32-augustus-gene-323.35

ATGGCACCAACGACGCTGACGGCTCTATCGGAGGAGAAGACCCTGAACGCCAAATTCGTTCGCGACGAGGACG
AGCGTCCCAAAGTGGCGTACAACACGTTCAGCGACGCGATTCCTGTGATCTCTCTCAGCGGGCTCGACGAGGTCGACGGA
CGGAGGGCGGAGATATGCAAGAAGATCGTGGAGGCCTGCGAGGACTGGGGGGTTTTCCAGGTCGTCGATCACGGCGTC
GACGCCGGCCTTATTTCCGATATGACTCGATTGGCTCGGGAGTTCTTTGCCTTGCCGCCAGAGGAGAAGCTGCGGTTCGA
TATGTCCGGTGGGAAGAAGGGCGGGTTCATTGTTTCTAGCCATTTGCAAGGGGAAGCAGTGCAAGATTGGCGTGAGATT
GTCACCTATTTCTCATACCCACTTCGCAACCGAGACTACTCAAGGTGGCCCGACAAGCCAGAGGGGTGGATCTCAGTGAC
TGAAAAGTACAGCGAAAAACTCATGGAACTTGCCTGCAAGATGCTCGACGTGTTGTCTGAGGCAATGGGTTTGGAAAAA
GATGCACTAACAAAGGCATGCGTGGACATGGACCAGAAGGTTGTGGTCAATTACTACCCCAAGTGCCCGGAACCAGATCT
CACTCTTGGGCTCAAACGCCACACCGACCCGGGCACAATCACCCTTTTGCTTCAGGATCAGGTTGGTGGACTTCAGGCCAC
CAAAGATGGTGGGAAGAATTGGATCACTGTTCAGCCCGTTGAAGGCGCTTTCGTTGTCAACCTTGGCGACCATGGTCATT
TTCTGAGCAATGGGAGGTTCAAGAACGCGGATCACCAAGCAGTGGTGAACTCAAACTACAGCAGACTGTCCATCGCCAC
GTTCCAGAACCCGGCTCCCAATGCAACTGTGTACCCGCTGAAGATCAGAGAAGGAGAGAAGTCAATAATGGAGGAACCC
ATCCCCTTCAGTGAAATGTACAGGAGGAAGATGACCAAGGACCTTGAGCTTGCCAGGCTAAAGAAGCTGGCAAAGGAGC
AGAACGCAGAAGCCCAATTGGAGGCCAAGCCCATTAAGGAGATTTTTGCTTAG

>VcF3H2K00475 snap_masked-VaccDscaff7-processed-gene-48.23

ATGGCTCCAACAACAACAACGCTCACTGCACTAGCAGAGGAGAAGACGCTTCAGAATAAATTCGTGAGAGACG
AAGACGAGCGTCCGAAGGTGGCCTATAACGAATTCAGCAACGAGATTCCGGTGATATCGCTCGCCGGAATCGATGAGGT
GGAGGGCCGGAGAGCGGAGATTTGTCGGAAGATCGTGGAGGCGTGTGAGGATTGGGGGATTTTTCAGGTCGTGGATCA
CGGCGTGGATTCGAGTCTGATTTCTGAGATGACTCGACTGGCTCGTGAGTTCTTTGCTCTGCCGCCGGAGGAGAAGCTCC
GGTTCGATATGTCCGGTGGGAAGAAAGGTGGATTCATTGTGTCCAGTCATCTTCAGAATCTCACTACTGTATGCCAGGGG
GAAGCAGTGCAAGACTGGAGGGAAATAGTGACCTACTTCTCATACCCAATCCGGGCCCGAGACTACTCGAGATGGCCCG
ACAAGCCTGACGGTTGGAGGGCCGTTACGGAGTCCTACAGCGAGAACTTAATGGGCCTGGCCTGTAAGTTGCTGGAGGT
GTTATCAGAGGCTATGGGCCTTGAGAAGGAGGCTCTAACAAAAGCCTGTGTTGATATGGACCAGAAAGTGGTTGTCAACT
TTTACCCGAAATGCCCACAACCCGACCTCACACTCGGACTCAAGCGACACACGGATCCGGGTACCATAACCTTGCTGCTTC
AGGACCAGGTTGGTGGGCTCCAGGCCACTAGAGATGGTGGAAAGACTTGGATCACAGTTCAGCCCGTGGAGGGAGCTTT
TGTTGTCAATTTGGGCGACCATGGTCATTATCTTAGCAATGGTAGGTTCAAGAATGCGGATCACCAAGCAGTGGTGAACT
CCAACCACAGCCGACTATCGATCGCCACGTTCCAGAACCCAGCACCCGAGGCAACAGTGTATCCACTTAAGATCAGGGAA
GGAGAGAAGCCAGTTCTCGACGAGCCAATCACGTTCATGGAGATGTATAAGAGGAAGATGAGCAAGGATCTTGAGCTTG
CAAAGTTGAAGAAATTGGCCAAAGAAAAGGTACTGGAGGAGCAGGAACTGGAGAAGGCCAAGTTGGAGACTAAAGGA
GAGGAGATTTTTGCTTAG

>VcFLS1 K05278 maker-VaccDscaff6-augustus-gene-163.26

ATGGAGGTAGAGAGAGTTCAAGCTCTAGCCCACGGAGGCCTCCACGAACTCCCCGCCCAATTCATCCGGCCCG
CGCACGAGCGGCCGGAGAACAGCAAAGCCATAGACGGGGTCACGGTTCCGGTAATCTCCCTCTCCAAGCCGCACGATAT
CGTCGTCGACGAGATATCGAGGGCTTGCAGCGAGTGGGGATTCTTCCTCCTCACGGATCACAACGTGTCGCCGGCGGTG
ATACGGCGGCTGAAGGAGGTTGGAGAGGAGTTCTTCAACCTCCCCCAGAAGGAGAAGGAGAGCTACGCGAATGACCCG
GCTAGTGGGAGGTTTGATGGGTACGGCACAAAGATGACTAGAAACCTTGATGAGAAGGTTGAGTGGGTGGACTATTATT
TCCATGTCATGTACCCTACAACCAAGGTCAATTATGACAATTGGCCCAAGAACCCTCCTTCTTACAGGGAAGCGACAGAGG
AATACAATGCAGAACTGCTGCAAGTAACAGATAAGATACTGGAGTTGCTCTCAGAGGGACTAGGTTTGGAAGGGAAGGC
ATTAAGGTCTCGTTTGGGCGAAGATGCAATGGAATTCGAAATGAAAATAAACATGTACCCGCCATGCCCACAGCCTGAAC

TCGCCCTCGGAGTCGAACCCCACACCGACATGTCTGCTCTCACCTTGCTTGTCCCGAACGATGTTCCCGGCCTTCAGGTTTG
GAAAGACGATAACTGGGTCGCTGTCGATTACTTCCCGAACGCGCTTTTCGTCCATGTCGGTGATCAAGTTGAGGTGTTGA
GCAACGGGAAGTACAAGAGCGTGCTTCACAGGAGCTTGGTTAACAAGGAAAGAACAAGGATGTCCTGGGCTGTGTTCGT
TACGCCTCCGCATGAAGCAGTGATCGGTCCTATTCCGGAGCTCATTGACGGGAAAAATCCTGCCAAATACTCAACCAAGA
CATATGCTGAGTATCGTCACCGCAAATTCAACAAGATTCCCCAGTAA

>VcFLS2 K05278 maker-VaccDscaff25-augustus-gene-225.24

ATGGAGGTGGAGAGAGTGCAAGCCATAGCTATACTATCCAAATTCATGGACACGATCCCGGCCGAATTCATCC
GGTCCGAGACCGAGCAACCCGCGATCACCACTGTGCGCGGGGAGGTGCTGGAGGTGCCGGTGATCGACCTCCTCGGCG
ATGAGGAAGAAGTGGTGGCGGCGGTGGCGACTGCGGGAGCTGAGTGGGGGTTGTTCCAGGTGGTGAACCACGGGATA
CCGGAGGAGGTGATAGGGAATTTACAGAGAGTAGGGAAGGAGTTTTTCGAGCTGCCGCAGGAGGAGAAGGAGATGTAT
GCGAAGCCTAAAGAGGGGAAGAGTATGGAAGGGTATGGGACGAAGTTGCAAAAGGAAGTTGAAGGGAAGAAAGGGT
GGGTTGATCATTTGTTCCACAAGGTTTGGCCTCCTTCTGCTATTAATTACCAGTTTTGGCCAAAGAATCCTCCTTCTTACAG
GGAAGCTAATGAGGCGTATACGGAGAAGTTGCGGGCAGTGGCGGACAAGTTGTTCAAGTGGCTGTCACTAGGGCTAGG
GCTGGACGGGCATGAAGTGAAGGCCGCGGTGGGTGGCGACGACTTGGAATACATGATGAAGATCAATTATTACCCACCC
TGCCCCCGCCCTGACCTTGCCTTAGGAGTGGTGGCTCACACTGACATGTCCACCCTCACACTTCTTGTTCCAAATGAAGTCC
CAGGACTCCAAGTCTTCAAGGATGACCATTGGTATGATGTCAAGTATATCCCCAATGCCCTTATTGTTCATATTGGGGACC
AAATTGAGATACTGAGCAATGGAAAATATAAAGCTGTACTGCACAGATCAACGGTGAGCAAGGACACAACCAGGATGTC
GTGGCCAGTGTTCTTAGAACCACCCCCAGAGCATGAACTCGGGCCGATTCCAAAACTCATTGGCGACAAAAATCCACCGA
AATATAAGACCAAGAAGTACAAAGACTATGTTTACTGTAAGCTGAACAAGCTTCTCCAGTGA

>VcHCT1 K13065 augustus_masked-VaccDscaff38-processed-gene-5.8

ATGAAGATCAAGGTGAAAGAAAGTTGTATTGTAAAGCCTGCCACAGAAACACCCAAACACGTACTAAAGAGCT
CCATTGTTGACCAAGTGTTTCCAAATTTCCATTCCCCAACTGTCTACTTCTATAGGCCAGATGGTTTGGAAAATTTCTTCGAC
TTTCGTTTGCTCAAGGAGGCTCTGAGCAAATGTCTAGTAACGTTCTATCCGGTGGCGGGACGGCTAAAGAAGAGGGAGG
GCAGTGCCTGTTTCGACCTAGATTGTAATGGAGAGGGTGTGTTGTTTGTGGAGGCAGAATCTGAAGATACTATTAATGAT
TTTGGTGATTTCGCCCCTTGCTCAGATCTCCTGCAACTTATTCCAAACGTTGACACTTCCGATGGATCTTCTTCCCATCCCCT
TTTGGTAGCACAGGTAACATCTTTCAAGTGTGGAGGAGCTTGTCTTGGACTCGGTTATCAACACATTCAAGGAGATGGAG
CGTCAGCCATATACCTCATCAATGCATGGTCCGACTTGACGCGTGGCCTCTCTGTCACCATTCCTCCGTTCTTCAATCGGTC
GTTACTCTGCGGCCGGGACCCACCTTCCCCGACGCACCACCACTCCGAATACGACCCACCGCCTTCCATGAACAAACCCCT
CCAAACCGTAGAAAGCCAAACAAGTCCAACATTGACTTCCACTGTCGTTATCAAGATCACACGTGATCAACTCAACACCTT
GAAAGCCAATTCAAGGGATGATAACAGCCCGGAAAGCTATAGCACATATGAGACCCTTACTGCACATGTGTGGCGAAGC
TTGTGCATCGCACGTGGACTTGGTGATGATCAAGCCACCAAATTGTACATCCCCACGGATGGGCGGTCGAGATTCCATCC
TCCTCTGCCACCCGGCTACTATGGCAATGTAATCTTCACAACCACATCTCTAGCCTTATCTGGCGATCTCCGGTCAAAGCCA
TTGTCTTGGGCTATTGAAAGAATACATGATGCAATATCCCGGATGGATGACGATTATCTACGGTCGGCTTTAGACATGCTC
ACAATATTGCAACCAAATCCACCGGCTCTACTTCGAGGGGCCCGCTACCTCATGTGCCCAAACCTTAATGTTGTGAGTTGG
GCAAGGTTGCCTATACATGATGCTGACTTCGGGTGGGGTCGGCCTATTCATATGGGCCCTGCCGATATATCTTTCGAAGG
AAACACATTCATATTACCCAGCCCAACTGGGGATGGGAGCTTGTCTTTGCTTATATGCTTACAGGATGATCATCTCAAAGC
CTTCAAGACCGCTTTCTATGACTTTTGA

>VcHCT2 K13065 maker-VaccDscaff24-augustus-gene-299.21

ATGAAGATCAAGGTGAAAGAAAACACTATTGTGAAACCAGCCGAAGAAACACCCAAACACATCCTAAGGAGCT
CCAACTTGGACCTATTGGTACCAAGCTTTTATGCACCAACAATTTACTTCTACAAGCCAAATGGGTCAAAAAATTTCTTTGA
CACACGTTTGCTCAAGGAGGCTCTCAGTAATTGTCTGGTGGTCTTCTACCCAGTGGCGGGGCGGCTGAGGAAAAGGGTC
GACGGTGGTGGCTTCGACATAGATTGTAATGGAAAGGGAGTGTTATTTGTGGAGGCAGAAGCTGACGGTGTTATTGATG
ATTTTGGCGATTTCGCTCCGTGCTCAGAAATCAAAAAACTTATTCCAACTGTTGAGTTTTCTGATGAATCTTTTTCCTTTCCC
CTTCTGTTGTTACAGGTAACATATTTCAAATGTGGAGGAGCTTCTCTTGGAGTAGGGTTTCAACACACTTTAGTAGATGGA
ATGTCAGCCCTCCACTTCATCAACGAATGGTCCAACATGACACGTGGCCTCTCCGTCGCCATCCTACCGTTCATCGACAGG
TCGTTACTCAATGGCCGCGACCCACCTTCCCCTACACACCACCATACAGAGTATGACCCACCACCTCCCATGAACAGTTCCG
TTCAAACCAAAGAACGCCAAGCAAGTCCCAAACACACTTCCATTGCCATTATCAAGATCTCACATGATCAGCTCAAAACCC
TCAAAGCCAATTCAAAGGACGATAAAAAACTAGAAACGTACAGCACCTATAAGATCCTTGCTGCACACGTGTGGCGATGC
TCGTGCATTGCACGTGGCCTTGGTGATGATCAAGCCTCCAAATTGTACATCTCAACAGATGGACGTTCCAGATTGCATCCT
CCTCTTCCGCCCGGCTATTTTGGCAATGTAATCTTCACCGGCACATTACTAGCCTTATCTGGTGATCTCCGATCACAGCCGT
TGGAATCAACTGCAAGAAAAATTCACAGTGTTTTATCCCGAATGGACGACGATTATCTGAGGTCAGCTCTAGACTACCTTG

AATTGCAGCCTGATCCAACAGCTTTGGTACGAGGATCCCATACCTTCAAGTGCCCAAACCTCAATATTGTGAGTTGGATTA
GGCTTCCTGTACATGATGCTGATTTCGGATGGGGCCGGCCAATTCATATGGGCCCTGCCGATGTACCTTTCGAAGGGAAC
GTGTACATACTACCAAGCCCAACTGGGGATGGGAGTTTGTCCTTGGTTGTGTGCTTAGAAGCTGATCATATGAAAGTCTTC
AAGACTGTTTTCTATGACTTCAGGAAAACCCATTCAGATGCCCCTAAACTCAATGTAGTCGAGGATTATTGA

>VcHCT3 K13065 maker-VaccDscaff27-snap-gene-291.30

ATGCTCAAATCTCTTCGGACTTCCTTTCTAATTTCCAAATTCAAAACCTCAATATACAGTTCAAGTTTCCGGCACA
AGTTAGCAAAAAACAATAGGCGCAGCTTATCAACGCGGCAGAGTTCGGCGCAAGTTAGCAACAAAATGAGGATCGAAGT
GAGGGAGTCGACGATGGTGCGGCCGGCGGAGGAGACCCCGGCGCGGCGGCTGTGGAACTCGAACGTGGACCTAGTCG
TCCCCAGCTTCCACACGCCGAGCGTCTACTTCTACCGCCCCAACGGCTCGCCCAACTTCTTCGACGCCGAGACGCTCAAGG
CGGCGCTCAGCCGTGTCTTGGTGGCGTTCTACCCGATGGCTGGGCGGCTGGCTAGGGACGAGGACGGCCGTATCGAGAT
CGATTGTAATGCTGAGGGGGTTTTATTCGTTGTGGCCGAGACCGGTGCGGCCGTCGACGATTTCGGGGATTTTGCACCCA
CGCTTGAGCTTCGGCGGTTGATTCCGGCCGTCGATTATTCGCTGGGAATCTCGGCGAATCCTCTGCTGGTGTTGCAGCTGA
CATATTTCAAATGCGGCGGCGTCTCCCTTGGTGTCGGTATGCAACATCATGCTGCAGATGGAGCTTCGGGTCTTCACTTTG
TCAACACATGGTCCGATGTGGCCCGTGGCCTTGACATCACAATCCCACCCTTCATCGACCGGACTCTCCTCCGTGCCCGGG
ACCCACCGCAGCCTCTTTTCCACCACGTAGAATACCAGCCCGCTCCTCCCCTGAAATCCCCTCCTCAATCGACAAATCCAGA
ATCTACCCCTGAAACTGCAGTTTCCATTTTTAAGTTGACCAAAGAGCAACTCAACACCCTCAAAGCCAAGTCTAAGGAAGA
TGGCAATACAGTAAACTACAGCTCGTATGCAATGCTCGCAGGCCACGTTTGGCGCTGTGTGTGCAAGGCACGTGGACTTT
CAGCTGACCAAGAAACCAAGTTGTACATTGCGACAGACGGAAGGTCCAGAATTCGACCCCCGCTTCCTTCAGGCTATTTTG
GTAATGTGATTTTCACCGCCACCCCTTTGGCCTTAGCCGGCGATCTCCAATCAAAGCCGACTTGGTACGCAGCCAGTAGGA
TTCACGATGCTTTGGCGCGAATGGACAACGATTACTTAAGATCTGCTCTTGATTACTTGGAATTGCAGCCTGACCTAAAGG
CCCTTGTTCGTGGAGCCCACACTTTCCGGTGCCCGAATCTTGGGATAACCAGTTGGACTAGACTGCCCATTCACGACGCGG
ATTTTGGGTGGGGGCGGCCGATATTTATGGGACCTGGCGGGATTCCGTATGAAGGGTTAGCATTTGCATTACCAAGTGCG
AGTAACGATGGGAGCTTGTCAGTGGGGATTTCTCTTCAAGCAGAACACATGAAGCTCTTTGCGAAGTTCTTGTATGACAT
ATA

>VcLAR1 K13081 maker-VaccDscaff25-augustus-gene-293.25

ATGACTGTGTCGAGTTTTATTGCTGGTGGCGCCGGTGGTGCAGCCAAGGGCCAGAGGGTCCTGATAATTGGAG
CCACTGGGTTCATTGGTCAGTTCATTGCTGAAGCAAGTTTGAGTGGTGGCCGACCCACTTATCTTCTTGTTAGGTCTGGTT
CTTCCAATGCCAAAACCATCAAAAGTCTGCAGGACAAAGGGGCCATGATCGTATATGGTGGCATGAAGGATCAAGAGTC
CATGGAGAAGATACTGAAGGAGAATGAGATAGATGTAGTTATTGCAGCCGTAGGTGGTGCTAATATCCTGGACCAGCTC
ACCCTAGTCCGTGCTATGAAAACTGTTGGAACCATCAAGAGGTTCTTGCCTTCCGAGTTCGGGCATGACGTGGACAGGGC
CGATCCGGTGGAGCCAGGTCTAACGATGTACAACGAGAAGCGGAGGGTTCGACGGTTGGTAGAGGAGTCAGGGATTCC
CTACACCTATATCTGCTGCAACTCCATTGCTTCGTGGCCTTACTACGACAACACTCACCCTTCCGAAGTCCACCCTCCCTTGG
ATCAGTTCAAAATCTACGGTGATGGCACCGTCAAAGCTTATTTTGTTGCTGGCTCCGACATCGGAAAATTTACGATCAAAT
CTATTGATGACATTCGTACTTTGAACAAACAAGTTCATTTCCGACCACCATGCAATTATCTAAACATAAATGAACTTGCATC
TTTGTGGGAGAAAAAAATTGGGCGGATCCTCCCCAGGGTTACAGTCACAGAGGATGATCTACTAGCTGCAGCAGCAGAG
AATATCATCCCACAAAGTATCGTTGCATCTTTTACCCACGACATATTCATTAGGGGATGCCAAGTTAATTTTTCAATTGAGG
GTCCTGATGAAGTTGAAGTGTGCGAACTCTACCCAGATGAATCATTCAAAACCGTCGATGATTGCTTTGAGGATTACGTG
GCGAAAATCGACGACAAAAGCATAGGCGTAACCGCCAATGGAATTGCCGCACAAAACCATGTCGTTGAAGCGCTGCCGA
TCACTGCAATGTGCGCTTGA

>VcLAR2 K13081 maker-VaccDscaff28-augustus-gene-232.26

ATGACGTTGATCACAGCTTCTGTTGCAGCAACCAAGGGCCGAGTCCTCATCGTTGGAGCCACTGGGTTCATAGG
CCAGTTCATAGCTGAAGCCAGCCTTGACTCCGGTCGAGCCACGTTTGTTCTCGCTCGCTCGTTTTATGATACCCCTTCTAAG
GCCAAAACCGTCAAGACTCTCCAAGACAAAGGCGCAACTGTGATACATGGAGTTGTTAGGGACCAAGAATTCGTGGAGA
GGGTACTGAAGGAGCACGAGATAGATATAGTGATTTCAGCTGTTGGTGGGGCCAAAATACTGGATCAGACCATCCTTGTC
CGGGCCATCAAAGCTGTTGGAACTATCAAGAGGTTTCTGCCTTCAGAATTTGGGCATGATGTGGACAGAGCTGATCCAGT
GGAACCGGGGCTCACCATGTACAAAGAGAAGCGCGAGATCAGACGGTTGATTGAGGAGTGTGGGGTCCCTTACACTTAC
ATCTGCTGCAACTCCATTGCTTCTTGGCCCTACTATGATAATACCCATCCTTCAGAGGTTCTTCCTCCTTTGGAGCAGTTCCA
AATTTATGGCGATGGAACTGTCAAAGCTTATTTTGTTGCTGGCGTTGATATTGGAAAATTCACAATGAAAACTGTTGATGA
CTTCCGTGCCCTCAATAAATCCGTTCATTTTCGGCCGTCTTGCAATTTTCTGAACATAAATGAGCTTGCTTCTTTGTGGGAG
AAGAAAATCGGACGAATCTTGCCCAGAGTCACAGTCACAGAAGACGACCTACTAGATGCTGCAGCAGAGAATATAATCCC
ACAAAGTATCGTTGCATCATTCACTCACGACATTTTCATTAAGGGTTGCCAAGTAAATTTTTCTGTCGATGGTCCTGACGAA

GTGGAAGTGAGCGATCTCTATCCAGATGAATCTTTCCGAACAATGGACGAATGCTTCAATGATTTCGTTTCGAAGATGGA
CATTGATAGGAGAAAGGGTGCTGCCGATGAGATCAATAGTACCAAAAATCATGTGGTTGAAGCTTTCCCAATCACAGCAA
TGTGTGCTTAA

>VcLAR3 K13081 maker-VaccDscaff9-augustus-gene-315.13

ATGACTGGTCATCGGACCCTGATCGTGGGTTCAACCGGATTCATCGGCCGGTTCCTGGCCGAAGCCAGCCTGG
GGTCTGGCCGGTCCACTTTCCTCCTCGTACGCCCCGGCCCAGCTTCCCCTTCCAAGGCCCACACCATCGCCTCCCTCGAAGA
AAAAGGCGCCATAGTCATACACGGTTCAATCGGAGACCAAGATGCGATGGAGAAGGTATTGAAGGAACATGAGATTGAA
GTTGTAATATCAGTCGTGGGTGGTGTTAGCGTCTTGGACCAGCTGATCCTCGTGGACGCCATTAAAAGTGCTGGCACCAT
TAAGAGGTTTTTTCCATCGGAATTTGGGCATGACATAGACAGAGCCGATCCGGTGGAACCTGGACTCACAATGTACAACC
AAAAGCGAAAAGTGAGAAGGGCTATAGAAGCAGCTGGGATCCCCTACACCTACATTTGCTGCAACTCCATCGCTGCCTGG
CCCTACCACGACAACACCCACCCCGCCGATGTCCTCCCACCGTTGGATTGCTTCCACATCTACGGTGATGGCTCCGTCAAA
GCTTATTTCGTTGCAGGGACTGATATTGGAAAATTCACGATAAAATGCGTGGACGACGAACGGACCGTGAACAAATCAGT
TCATTTCCAGCCACAATCCAATCTACTGAACATGAATGAGCTAGCATCTTTGTGGGAGAAGAAGATTGGACGCACTCTCCC
TAGAGTTACCATCACTGAAGATGACCTTCTTGTGGCTGCTAAAGAGATGTGCATCCCAGGCAGCATAGTTGCATCATTGAC
CCATGACATCTTCATTAAGGGTTGCCAAGTAAATTACAGCCTCGATAGAGACACGGATACGGAAGTGACCTCCCTCTTTCC
GGGCGCCTCTTTTCAAACCATCGATGAATGTTTTGACAACTTCCTTGCTAGGATCATCGATACTGATCCGGTTGTTGTCGTT
GATGAACCAACCACCGAAGACACCATTGGTGCATCAAATCCCGAAAGAAAAGCATTCGCCATCACCGCGTCTTGCTCATG
A

>VcPAL1 K10775 maker-VaccDscaff19-augustus-gene-331.32

ATGGAGTGCTCGCAACAAAACGGCAACGGCCACGCGGCGAAGGCGGACAGTTTGTGTGTCATGAAAGATCCA
TTGAATTGGGGTATGGCCGCGGAGTCATTGAAAGGAAGTCATTTGGAGGAGGTGAAGAGGATGGTGGAGGTGTTTCGG
CAGCCGGTGATCCGGCTAGGAGGGGAGAGCCTTACCATAGCGCAGGTGGCTGCTGTGGCGGCAAGGGAGGACGCCGG
GGTGGCGGTGGAGCTGTCCGAGTCGGCGAGGGCCGGTGTGAAGGCTAGTAGTGATTGGGTTATGGAGAGTATGAACAA
AGGGACGGATAGTTATGGGGTTACCACTGGGTTTGGTGCAACATCTCATAGGAGGACTAACAAAGGTGCTGCTCTTCAAA
AAGAGCTCATAAGATTTTTGAATGCTGGAATATTTGGCAATGGGACAGAGTCAACCCACACATTGCCTCACTCTGCCACAA
GGGCTGCCATGTTAGTGAGAATCAACACTCTCCTTCAGGGGTACTCAGGCATTAGGTTTGAAATTCTGGAAGCCATGACA
AAGCTCCTCAACAAAAACATCACTCCTTGTTTGCCGTTGCGCGGTACAATCACCGCCTCCGGTGACCTAGTCCCCTTATCGT
ACATCGCCGGAGTTTTAACAGGCCGGCCAAACTCCAAATGTGTTGGGCCCAATGGAGAGGCCCTTGATGCTGCCGAGGC
GTTCCGCCTAGCCGGAATCGAGAGTGGGTTCTTTGAGTTGCAGCCCAAAGAGGGCCTTGCCCTAGTGAATGGCACAGCTG
TTGGGTCTGGTTTGGCCTCTGTTGTTCTATTTGAGGCCAATTTACTAGCTGTTTTGTCTGAAGTTATTTCAGCAATTTTTGCT
GAAGTGATGCAGGGGAAACCAGAATTTACTGACCATTTGACTCACAAATTGAAGCATCACCCTGGCCAAATTGAAGCTGC
AGCTATAATGGAGCACATTTTGGATGGGAGTGACTATGTTAAGGCTGCACAAAAGTTACATGAAATGGACCCCCTCCAGA
AGCCTAAACAAGACCGATACGCCCTCCGTACCTCGCCCCAGTGGTTGGGCCCGTTGATCGAAGTCATTCGTTCCTCGACTA
AGTCGATCGAGCGGGAGATCAATTCGGTGAACGACAACCCCTTGATCGACGTGTCGAGGAACAAGGCCTTACATGGTGG
GAATTTCCAGGGTACCCCAATTGGTGTCTCAATGGACAACACTAGATTGGCTCTGGCTTCAATTGGTAAACTCATGTTTGC
TCAATTCTCTGAGCTAGTCAATGACTTTTACAACAATGGGTTGCCTTCAAATCTCTCCGGCGGGCGCAACCCGAGTTTGGA
TTACGGGTTTAAGGGCGCAGAAATAGCCATGGCGGCCTATTGTTCCGAGCTCCAGTTCCTGGCTAACCCAGTAACCAATC
ATGTCCAAAGTGCTGAGCAACACAACCAAGATGTGAACTCGCTCGGGTTGATTTCGTCGAGGAAAACAGCCGAGGCGAT
TGACATTTTGAAGCTCATGTCGTCTACCTACTTAGTTGCCATTTGCCAAGCGATAGACCTGAGGCACATGGAGGAGAATTT
GAGGAATACGGTTAAGAACACGGTGAGCCAAGTGGCTAAGAAAGTCCTCACAATGGGTGTCAATGGGGAGCTTCACCCA
TCAAGGTTTTGTGAGAAGGACTTGCTGAAAGTTGTGGATCGCGAGCACGTTTTCGCCTACATTGATGATCCGTGCAGTGC
GACGTACCCACTTATGGTGAAGCTAAGGCAAGTCCTTGTTGAGCACGCGCTGACAAACACCGACGATGTCAAGAACGCG
AACAATTCAATCTTTCTGAAGATTGGTGCTTTTGAGGAGGAACTCAAGAACCTTTTGCCGAAAGAAGTTGAGAGCATGAG
ATGTGCCGTGGAGAGTGGAAACCCAGTTGTTCCAAACAGGATCATGGAGTGCAGGTCTTACCCATTGTACAAGTTTGTGA
GGGAGGAGTTGGGTACCGAGCTGTTGACGGGAGAGAAGATCACGTCGCCCGGTGAGGAATGCGATAAGGTGTTTTCTG
CGATCTGCGAAGGGAAGATGATCGATCCGCTGCTCGAATGTCTCAAAGACTGGAATGGCGCCCCTCTGCCAATTTGCTA

>VcPAL2 K10775 maker-VaccDscaff10-augustus-gene-222.29

ATGGGCTCACTAATGGAAATTGAGTACACAAATGGGTCCGGCCCGGGTTTCTGCCTGAAGGACCCGTTGAACT
GGGGAGTGGCGGCGGAGGCGCTGAAGGGGAGTCACCTGGATGAGGTTAAGTCGATGGTGGCGGAGTTTCGGAAGGGA
GTGGTGAGGCTGGGTGGAGAGACGCTGACGATATCGCAGGTGGCTGCGATAGCGGCTGCTAGCGGAGGGGAGGTGAA
AGTGGAGCTGTCGGAGTCGGCGAGGGCCGGGGTGGAGGCGAGTAGTAATTGGGTTATGGAGAGTATGAATAAAGGGA

CGGATAGTTATGGGGTGACCACTGGGTTTGGGGCTACCTCTCATCGGAGGACTAAGCAAGGTGGAGCTCTTCAAAAGGA
GCTTATTAGGTTCTTAAACGCTGGAATATTTGGCAAAGGAACAGAGTCTGGCCACACGTTGCCCCACTCAGCCACAAGGG
CAGCGATGCTTGTTCGCATCAACACCCTCCTCCAGGGTTACTCTGGCATTAGATTCGAAATCCTGGAAGCCATTGCGAAAT
TCCTCAACAACAACGTCACCCCATGCCTCCCCCTCCGCGGTACCATCACCGCCTCGGGCGACTTGGTCCCTCTCTCTTACAT
TGCTGGGCTCTTGACAGGGCGGCCTAACTCAAAAGCGGTAGGGCCTGATGGAGAAATGCTCAAGCCCGAAGACGCCTTC
CGTGTGGCTGGAGTCAATGGCGGGTTTTTCGAGCTCCAACCCAAAGAAGGCCTTGCCCTAGTCAACGGAACCGCAGTGG
GGTCTGGCATGGCTTCTATGGTCCTATTCGAGGCTAACATACTTGCCGTTCTATCGGAAGTTATGTCGGCGATTTTCGCCG
AAGTAATGCAAGGAAAGCCCGAATTCACAGACCATTTGACGCACAAACTCAAGCACCATCCAGGCCAAATAGAAGCAGCT
GCTATCATGGAACACATTTTGAATGGAAGTGCTTATGTTAAGGCAGCAGAAAAGCTCCATGAGATGGACCCCCTGCAAAA
GCCAAAACAAGATCGATACGCCCTCCGAACGTCCCCGCAATGGCTAGGACCGCAAATCGAAGTCATCCGATCATCGACAA
AGTCAATCGAACGGGAGATAAATTCGGTGAACGACAACCCTTTGATCGACGTTTCAAGAAACAAGGCCTTACACGGAGG
AAATTTCCAAGGGACCCCCATTGGAGTCTCGATGGACAACACCCGTCTAGCCATCGCTGCAATTGGGAAACTCATGTTTGC
GCAATTTTCAGAACTTGTCAATGATTATTACAACAATGGGTTGCCTTCAAACCTATCCGGGGGACGAAACCCAAGCTTGGA
TTATGGGTTTAAAGGAGCTGAGATTGCCATGGCTTCTTATTGCTCTGAACTTCAATTTCTAGCCAATCCAGTGACTAACCAT
GTCCAAAGTGCAGAGCAACATAACCAAGATGTCAACTCTTTGGGCCTGATTTCCTCAAGAAAAACGGCTGAAGCTGTTGA
TATATTGAAGCTGATGTCCTCCACATATCTTGTAGCTCTATGCCAAGCCATTGATTTGAGGCATTTGGAGGAGAATTTGAG
GAGCTCTGTTAAAACCACTGTAAGCCAAGTAGCTAAGAAAGTTTTGACTATGGGGGTGAATGGAGAACTTCATCCTTCAA
GGTTTTGCGAGAAGGATTTGCTCCGAGTGGTTGATCGCGAATCTGTATTCGCGTACATTGATGACCCTTGCAGTGGAACCT
ACCCATTGATGCAGAAACTAAGGCAAGTTCTCGTGGAGCACGCATTGAAAAATGGCGAAAATGAGAAGAATATGGGGAC
TTCGATTTTCCAAAAGATTGAGGCCTTTGAGGAGGAATTGAAGGCAGTTCTGCCCAAGGAGGTGGAGAGCACGAGAGTG
GCTGTGGAGAGCGGGAAACCGGCGATTCCAAACTTGATCAAGGACTGCAGGTCTTATCCACTTTATAAATTCGTGAGAGA
GGAGATGGGGACGGCGTTCTTGACCGGAGAGAAGGTGGTGTCACCGGGGGAGGAGTGCGATAAGATTTTCACGGGGTT
GTGTGAAGGGAAGGTGATTGATCCTTTGATGGACTGTCTCAAGGATTGGAATGGTGCTCCTCTTCCAATCTGTTAA

>VcPAL3 K10775 maker-VaccDscaff29-augustus-gene-173.18
ATGGAATTTGATCAAGGTCAATGCAATGTTCATGACACGATCTCCTTGTGTGTTCAAGACCCATTGAATTGGGG
TAAGGCTGCAGAGTCCTTAAAGGGAAGTCACTTGGATGAAGTTAAACGTATGGTGGTGGAGTACCGGAAGCCGGTGGTC
CGGCTTGGTGGCGAGACCCTAACCATATCACAGGTGGCCGCGATAGCCACCCGTGATGCCAAGATCACGGTGGAGCTAT
CGGAAGCTGCCAGGGCGGGCGTGGTGGCAAGTAGTGATTGGGTGATGGAAAGCATGAACAAGGGGACCGATAGCTATG
GTGTGACAACTGGTTTTGGAGCAACTTCTCATAGGAGAACCAAACAGGGTGGAGCACTTCAAAAAGAGCTTATTAGGTTC
TTGAATGCTGGAATATTTGGTAACGGGACAGAATCTTCCCATACTCTGCCTCGCTCAACAACACGGGCCTCAATGCTTGTG
AGAATCAACACCCTCCTTCAAGGGTATTCAGGCATTCGATTTGAGATCTTAGAAGCCATCACTAAGTTTCTTAACCATAACA
TCACCCCGTGCTTGCCCCTCCGTGGAACAATCACTGCCTCGGGCGACTTGGTCCCTTTGTCCTACATTGCTGGGCTTTTAAC
TGGAAGGCCCAATTCAAAGGCTGTTGGGCCCAACGGAGAATTGCTCAATGCCATTGAGGCCTTTCACCTTGCGGGGATTG
ACACCGGGTTTTTCGAGTTGCAGCCCAAAGAGGGTCTTGCACTAGTGAATGGCACAGCTGTTGGGTCGGGCTTGGCTTCC
ATAGTTCTTTTCGAAGCCAACATTCTCTCCCTCCTTTCGGAAGTTTTATCTGCTGTATTCACAGAGGTCATGCAAGGAAAAC
CCGAATTCACAGACCATTTGACTCATAAATTGAAGCACCACCCGGGCCAAATTGAGGCTGCAGCTATAATGGAACACATTT
TAGATGGAAGCTCATATGTCAAAGAATCCCAAAGGCTCCATGACATTGATCCACTCCAAAAGCCCAAACAAGACCGATAC
GCTCTCCGTACATCTCCCCAGTGGCTCGGCCCGCAGATCGAAGTGATTCGATCTGCTACCAAAATGATCGAACGGGAGAT
AAACTCCGTGAATGACAACCCACTTATTGATGTTTCCCGTAACAAGGCACTACACGGAGGGAATTTCCAGGGCACCCCAAT
AGGGGTCTCAATGGACAATACCCGGTTAGCCATCGCCTCGATAGGGAAACTAATGTTCGCTCAATTTTCCGAGCTTGTCAA
TGACTTTTACAACAATGGGTTGCCCTCGAATCTCTCGGGTGGGCGTAACCCTAGCTTGGATTACGGGTTCAAAGGTGCGG
AAATCGCGATGGCGGCCTATTGCTCTGAGCTCCAGTTCTTGGCTAACCCTGTAACCAATCATGTCCAAAGTGCTGAGCAAC
ACAACCAAGATGTGAACTCATTGGGCTTGATCTCATCAAGAAAAACAGCTGAGGCACTTGACATACTAAAACTCATGAGT
GCCACTTGGTTGGTAGCCTTATGCCAAGCAATTGACTTGAGGCATTTGGAGGAAAACCTAAAGAGCATAGTAAAAAGCAC
AGTGAGTCAAGCTGCCAAGAGGGTTCTAACCATGGGTTCAAATGGACAACTCCACCCCTCAAGATTTTCAGAGAAAGAAT
TGCTCAAAGTGGTGGACCGGGAGCTCGTATTCACTTATATCGATGACCCGGTGAGCAACACGTACCCATTGATGCAAAAA
CTCCGACAAGTCCTGGTGGAGCATGCGCTGGCAAACGGTGAGAGTGAGAGGAATCCTAGTACGTCGATTTTCCAAAAGA
TCAGCGCATTTGAGGAGGAGCTAAAGAGCGTTTTGCCCAAGGAAGTCGAAATTGCCCGGTGCGACGTGGAGAGTGGGAT
GGCAGGTATTGGAAACAGGATAAAGGAATGTCGGTCGTACCCATTGTACAAATTCGTGAGAGAGGATTTGAGGACCGGG
TTTTTGACCGGAGAGAAGATCCGGTCACCGGGAGAGGAGTTTGATAAGGTGTTCTCTGCCATTTGTGAGGGGAAGTTGA
TTGATCCTTTGCTGGATTGCGTGAAAGAGTGGAATGGTGCTCCTTTGCCTATTTGCTAA

>VcbHLH075 maker-VaccDscaff17-augustus-gene-313.25

ATGTCAGTTTTTCCCCAACAACACCAGCCTTTTCTGCTGGACTCTTCATTTTTCACTCCCATTAAGATCATGTCTGG
GTTTTCGGAGGAACCAAACACCCCCTTGTTTTCTCAATTGTACCCACCTGAATCTGTTCACCAAATCCCTGTTCATGAATTTA
GTAGCTGCCTTGAAAACAGCACAAGATTTGATGTTAGTACTAATGGTGATGATTCTTCATCAGTAGTTGATAACAAGGCTG
AAAGTGGTGAGCAAGTCACTCAGAAGTTGATTCCCATGGACAAGAAGAGGAAAAACAGAGATGGGTCTGCTCAATCCAA
GGATGCAAGAGAAGTGAAGGGGAAAAATCAAATGAAATGCAATGGTGTGATGAAAGGTAATGAAGAGAAGAAGACTA
AAGAAAATAAGAAGGATCAGAAGAAGAAGGTCAATGAAGAAGCCCCAACAGGGTTCATTCATGTAAGGGCCAGAAGGG
GGCAAGCAACAGACAGCCACAGCTTAGCAGAGAGGGTGAGAAGGGAGAAAATAAGTGAGAGGATGAAGCTACTGCAG
GCTCTTGTTCCTGGATGTGACAAGGTTACTGGGAAGGCCCTCATGTTGGATGAAATTATCAACTATGTCCAGTCCCTACAA
AATCAAGTTGAGTTCCTATCTATGAAGCTTGCTTCAGTGAATCCCATGTTCTATGACTTTGGAATGGACTTAGATGCATTCA
TGGTTCCACCTGAGATCAGACTGAATGGATTGGTATCACCATTGTTAATGCCAAATCTGCAAAAATGCAACCACCACCAGG
CCACTGCCTTTTCTGATGCAAGCGCCGCGGCCGCCACCGCCGCGACCTCCACCACTTTGACAATACCAAATAGCTATGCTC
TTGTGGACACACCTACATCTACATCACTTTTGCTTCAACAAGTGCAGAGGCCAAATATTCTGTCCCAGGATACTGAACAGC
TATTGTGGGATGTGGAGGACCAGAGACAGAAACTTACTTATCAATCTGACTTCAGCAACAACTTGTGCTCTTTCCATTAA

>VcbHLH1 maker-VaccDscaff22-snap-gene-21.29

ATGTGTTGGGGAATGGCTAAAGGGGCACAAAACCAACAAGGGGTGCCAGAAAATCTGAGAAGGCAGCTTGCT
GTTGCTGTTAGGAGTGTGCAGTGGAGCTATGGAATTTTCTGGTCATTGTCAACCAAACAAGAAGGGGTATTGGAGTGGG
GTGATGGGTACTACAATGGGGACATAAAGACGAGGAAGACAGTGCCGGCCATGGAACTTAAACCAGATAAAATGGGTTT
GCAGAGGAGTGAGCAACTGAGACAGCTTTACGAGTGTCTCTTAGAGGGTGAAGAAAGTGATCAACAAGTGCCTTCTGCC
GCACTGTCTCCAGATGATCTCACAGATGCAGAGTGGTATTACTTGGTTTGCATGTCCTTTGTGTTCAACCCCGGCCAAGGG
TACAAGCTTTCTTCTGGACATTTTCATCTTTCTCTGTGGCTTTTGCCAGGAAGGGCATTGGCAAATGGTCAAGCTATCTGGT
TGTGCAATGCTCACAGTGCTGATAGCAAAGTGTTTTCTCGCTCTCTTCTAGCAAAGACTGTTATTTGTTTTCCCTATTTGGGT
GGAGTCATTGAGCTAGGCGTGACGGAGCTGGTATTGGAAGACCCTAGTCTCATTCAGCACATCAAGACTTCTCTATTAGA
GTTTTCAAAGCCCGTTTGCTCTGAGAAATCTTCTTCTGGTCCTCCCCACAGAGACGATGACGCTGACTTCATGTATGCCAAG
GTTGATCGTGAGCTGGTAAATACAATTTCGTTGGAGAGTTTCTATTCCCCAACTGAAGATATGAGATTTGATCGGGAGGG
ACTAAATGACTTTGAAAAAAATATCCAGGCCGATTTCACTATGGGTTCTCCTGACGAATGTTCGAATGGGTGTGAACACAA
TCACCAGACAGAAGACTCCTTCCTCCTTGATAATGTAACTTCTCAAATGGAAGATGATTTTAGCAACTGTGTTCAAGTTTCC
ATGAATTCTAGTGATTGCATATCTCAGGTCGTTGCGAACCAAGAAAAGTTTATTTCTCCTCCCAAGGGTGACAATAGTAAA
TTGAAAGAACTTCAAGAAGGGAATGATACAAAATTTGGATCCTTGGACATTGGAACTGATGATGACTTGCACTACAAAAG
AACTCTATCTGCTGTTCTGATAAGAAATTCTCATAGGTTACTTGATAACCTTTGCTTCCATAGGTGGGATTATAAGTCTAGC
TTTGTGAGTTGGAAGAGAGGAGAAATCAGAAATGGCCATAGACCACAGGTGCCGCAGCAGATGTTAAAGAAGATATTAG
TTGATGTCCCTCTAATGCATTGTTTCTCACTCAAGTCTCAACAAGGAAATGTGGGGAAATTTTGGTTGTTAAAACAAGAAA
GTTATGTGCAACATGATTTGCCGGATGATGGAATAGAGAATGAGAAGTTTTTGGTCCTCAAATCGATGGTTCCTCCTTCTG
TTAGCAAGGTTGACAAAGCAGCAATACTTGGTGACACTATAGAATACTTGAAAGAACTTGAGGGAAGGGTAGGAGAACT
TGAATCTTGCATAGATTTAGCGGAGCATGAAGCTAGAAGAAACTACCCAGATGAAATAGAGCAGAAATCTGAAAACAAT
GAAAACAGAAGGATTGGAAATAGCAATAAGCCTTGGATAAACAAGAGGAAGGCCTGTGATATTGATGAAACAGACCCTG
AGCTCAGCAGGGTTGTTCCTAAAGATGGGCCACTGTTTGATATGAAAGTAAAGATAAAAGAGCAGGAAGTCCTGATTGA
GATGAGATGCCCTTCCAGGGAATATTTGTTGCTTGATATCATTGAAGCTCTGAACAATCTCAGCTTAGACGCTCACTCTGTT
CAGTCATCAACCATTGACGGCATTCTCACTGTAACCCTTCAGTCTAAGTTTCGAGGAGCAGCAGTTGCATCAGCACGGATG
ATCAAACAAGCGCTTTCAACTGTTGCCGGAAAGTGTTGA

>VcbHLH2 maker-VaccDscaff19-augustus-gene-381.30

ATGGCCGCACCACCTAGTACACGGCTGCAAAATATGTTACAGACGGCGGTGCAATCAGTGCAATGGACGTATA
GTCTCTTCTGGCAACTGTGCCCTCAACAAGGGATCTTAGTATGGGGAGATGGGTACTACAATGGAGCAATCAAAACAAGA
AAGACAGTACAACCAGTGGAGGTGAGTGCCGAGGAGGCGTCGCTACAAAGAAGCCAGCAGCTGAGAGAATTGTACGAT
TCCCTCTCCGCTGGCGAGTCGAACCAGCAGACACGGCGGCCGTGTGCCTCCCTGTCGCCGGAAGACTTGACGGAGTCCGA
GTGGTTCTACCTCATGTGCGTGTCCTTTTCTTTTCCTCCTGGTGTTGGACTGCCGGGAAAAGCTTACGCAAAGAGGCAACA
TGTTTGGCTTACCGGGGCAAACGAAGTCGACAGCAAAGTGTTTTCTAGAGCCATTCTTGCCAAGAGTGCTCGCATTCAGA
CTGTAGTGTGTATTCCTCTACTTGATGGCGTTGTGGAGCTCGGCACAACAGAAAGGGTTCAAGAAGACATTGGCTTGGTA
CAACAAGTAAAGAGCTTCTTCATAGACCACCACCCTCCACAGCCTCCGAAACCCGCCCTCTCCGAGCACTCCACTTCCAACC
CCGCCACCTCCTCCGATCACCCCCGTTTCCACTCTCCTACAGTACCAAACGTTCACGCACCCGTAAATCCTCCTGTAAACCCC
AATCAGATCGACGAGGAGGAAGAAGAAGAGGAGGAGGACGAAGAAGATGAAGAGGAGGAAGAAGAAGAAGGGGAG

TCGGAATCAGAAGCTGACACGGGGCGGAATAGCCACCTTTCGGGGGCTCGGAACCCACGGGTAGTAAATGTGGCCCAAG
CAGCCGCCGAGCCGAGTGAGCTAATGCAGCTTGAGATGTCTGAGGATATCCGCCTTGGCTCGCCGGATGACGGTTCGAA
TAATTTGGACTCGGAGTTTCAGTTGCTGGCAGCGAGCCAAGGGGGAAATGCGGTGGATCATCAACGGCGAGCAGACTCA
TATAGGGCCGAGTCAACTCGGAGGTGGCCATTGTTGCAGGACCCCATGAGCAGTAATCTCCAACCACCTCCTTCAGGAGG
GCCCGCACTGGAAGAATTAACACAAGAGGACACGCACTACTCCCAAACAGTCTCCACCATTCTCCAACACCAGTCCACCCG
GTGGTCAGACTCATCAACCTCCTCCGCCGCCGCTGCTCCCGGCTACCTAATGTACTCCTCCCAATCCTCATTCTCCAAGTGG
AATCTCCATCCCTCCGACCACCACCACTACGCAGCCTCAGCCGCCGACGGCACCTCCCAGTGGCTCCTCAAATACATCCTCT
TCTCCGTTCCCTTCCTCCACACAAAGTACCACGACAACAACAGCAACTCCCCCAAATCTGCCTCCGCCGCCGCCAACGACTC
CGCCTCCCGATTCCGCAAAACGACGCCGCAGGACGAGCTCAGCGCCAACCACGTCCTCGCCGAGCGCCGCCGCCGCGAA
AAACTCAACGAGCGGTTCATCATCTTGCGATCGCTCGTCCCTTTCGTTACCAAAATGGACAAGGCCTCGATTTTAGGTGAC
ACGATCGAGTACGTCAAGCAGCTGCGGAAAAAAATCCAGGATCTAGAATCGAGGACGCGTCAGATGGAATCCGATCAAC
GGTCGAGATCGTCTGGGGACCTACAGAGAACGAGTAGTTTGAGTTTAAAAGAACAGAGGAGCGGAGTTACGACGGTTGT
GAATACGGATCGGGCCAATAGGGTGGGGGGTGGGCCAACCGGGTCGGATAAGAGGAAGTTGAGGATAGTGGAGGGGG
GAAGTGGAGGAGCGAAGGGGAAGGCGGTGGATTCGTCGCCGGTGGAGGTTCCACCGTCTCCGCCTCCTCCTCCTCCTCC
CCCACAGCCGGTGGCGGGAGTTGGGTTGCAGGTGCAGGTGTCGATTATAGAGAGTGATGCGTTGGTGGAGCTGCAGTG
TCCGCATAGGGAAGGGCTGTTGTTGGATGTGATGGTGGTGTTAAGGGACCATCGGGTGGAGGTTACGGCGGTGCAGTC
GTCGTTGACTAATGGGGTTTTTGTTGCAGAGTTGAGAGCTAAGGTGAAAGACAACGGCAGCGGGAAGAAGCCAAGTATT
GTGGAAGTAAAGAGGGCAATACACCAAATCATACCCTCGTACTGA

>VcMYB4 maker-VaccDscaff37-augustus-gene-96.16

ATGGGAAGATCACCTTGCTGTGAGAAAGCTCACACAAACAAAGGCGCGTGGACCAAGGAAGAGGACACGCGC
CTCATCAACTACATCAGGGTTCACGGCGAGGGCAACTGGCGCTCCCTCCCCAAATCCGCCGGATTGCTGCGATGTGGAAA
GAGTTGCAGACTGCGGTGGATAAATTATCTGAGGCCTGATCTGAAGAGAGGGAATTTCACTGAGGAAGAAGACGAACTC
ATTATTAAGCTTCACAGCTTACTCGGTAACAAGTGGTCATTGATTGCCGGAAGATTACCCGGTAGAACCGACAACGAGAT
CAAGAACTACTGGAACACTCACATCAAGCGAAAACTCGTCACCCGAGGCCTCGACCCACAAACTCACCGTCCCTTCAAGTC
CACCACCACCACTACCTCCGTCACCAATTCCCAATTCGACTTCACAAACACTTCTCAATCGCCTTCACCCCCATCCGACATCG
TTTTAACCGGTCAATCTACGGCGAAGCATCAACTCAGAACCGCCGCGGATTCAATCGAGTGCAGCAGTGGCACCACTGAG
GAGACTCGGCCCCAGCCACTGGCCGAGTCTCCTCAGTCGATTGGGAGATTCGATCCGATGGGTTCTGCGGCTCCAGATCG
ATCGACTGGCAAATTTGAGCCGATCGATCTGGAGCTGTCGATCGGGCTTGTCCCGTTCCAGTCCCGATTGCCGAGTTTTTC
GGCGAACTCGGCCGAGTCGAAGGTTGTGTGCTTGTGTTGGCAGTTGGGGTTTCGGGGTGGTCAATTGTGTAGTAATTGT
GAAAGCAGTAATACAAGGAGGTTTTATAGTTAG

>VcMYBA maker-VaccDscaff1486-snap-gene-0.3

ATGGACATAGTTCCATTGGGAGTGAGAAAGGGTGCTTGGACTGAGGAAGAAGACTGTCTTCTCAAGAAGTGCA
TTGAGAAGCATGGAGAGGGGAAGTGGCACCAAGTTCCTTACAGGGCAGGATTGAATAGATGCAGGAAAAGTTGTAGGC
TGAGATGGTTGAATTATCTGAGGCCAAATATAAAGAGAGGAAATTTTGCTGTGGATGAAGTTGATCTCATTATCAGGCTT
CATAAGCTGCTAGGCAATAGGCAAGCGCGCCCTCTCTCTCTCGGAATTTTCATTTCATGGTCCTTAATTGCGGGTAGACTTC
CAGGAAGAACATCGAACGACGTGAAAAATTACTGGAATACCCATCTAAAAGAGAAATCGACGGACCAAAGTGGAGAGGT
ACAGAAATCTAAAACGACCTTGAATACGACTGAAAGGACCACAATCATACGGCCTCAACCACGAACCTTCCCCAAAAATC
GACGTGTTTTGATGGGTAGTACTGTCATTGCAGATAATATTCAAACAAGAGATCCAAATCTCTCCAACCCATCCCAAACAC
AACCACCGAGGGATGATGATGGAACATTGTGGTGGGATGACATGTTGTTCGATTATGAAATTAGCAGAGGTATGATGAC
GTGGACCAATGACGGATCAAATGAGGAGGCCATGATGGTGGATAACGGTGAAGAAGCAAAATCAGGTACACAAGGAGT
TGGTGGGGATCATTACAGTTGTGTTCAAGAAGATCAAAGTGATTGGAGTAATATTTTTATGGACAATGTGGACCTTTGGG

>VcMYBC2 maker-VaccDscaff28-augustus-gene-197.19

ATGAGGAAGCCATGTTGTGAGAAGCAAGACACGAATAAAGGAGCATGGTCCAAACAAGAGGATCAGAAGCTC
ATAGATTACATTCGCAGGCACGGTGAAGGCTGTTGGCGTACCATCCCGCAGGCTGCAGGACTACTTCGTTGTGGTAAAAG
TTGTAGGCTAAGATGGATTAATTACCTGAGACCAGACCTCAAAAGGGGCAACTTTGCCGAAGATGAAGAAGATCTCATCA
TCAAGCTTCATGCACTCCTGGGTAACCGGTGGTCATTGATAGCAGGAAGATTGCCAGGGCGAACCGACAACGAAGTCAA
GAATTACTGGAACTCCCATTTGAGAAGAAAGCTTATGAACATGGGTATCGATCCAAATAATCACCGGTTAAATCAGAATCT
CCCCCCTCGCTCTCAAAATCCACGTCCCTCTCCCACTGCAACAGCTTCTTCTGCTTCTAAACACGACGTATCTCAGCCATTGA
AGACCAAGCTAGCGGAGAATGATCAGGTCTCCGATGCTGCAAGTTGTCTAGACGACGACTCCACACCGTGCTTGCCTGAT
TTAAATCTTGATCTTAAAATTACCCTGAATCATTCTTCTTCAATTTCCATTGATATTGTTGAAGAGGAGAAGAAAGAAATTG
ATCAATCCACATTATCAAGGGCTGTTGAATCCAACCTTTGCCCCACCCTTCCTCTCTTTACATGATACTTTTCTAATGGAAAA

AATGATCAATAGAGAAAAAGAAAATATTTGGCAAGGGGAGCTCGAAACCATCGAACGAAAAAATAAAAAATTTGATTTG
CGGTCTCATTTTGATGAACTGCATCATTTCGCTGAAGATTTTTAA

>VcMYBPA1 maker-VaccDscaff39-snap-gene-168.25

ATGGGAAGGGCTCCCTGTTGTTCAAAGGTCGGGTTGCATAGAGGTCCATGGACTGCTAAAGAGGACTCATTGC
TTTCAAAGTACATTCAACTTCATGGCGAAGGCAACTGGAGATCTTTGCCTAAAAAAGCAGGGTTATTTAGATGTGGAAAA
AGTTGCAGGCTGAGATGGATGAATTATCTGAGACCAGATATTAAGAGGGGAAACATCACCCCTGACGAGGATGACCTCA
TTATAAGAATGCATGCCCTTCTAGGCAATCGATGGTCTCTAATTGCGGGAAGATTACCAGGTCGAACCGATAACGAGATA
AAGAACTACTGGAACACTCATCTTAGCAAAAGACTCCGAGACCAAGGAACCGACCCGAGTACCCACAAAAAATTATCGGA
ATATCCTAACGACCAACCACCAAAGAAGAGGAGGAACAACAATAGAAAGAAGAACAAGTCAAATTTGGAGGCCCGAAA
GCAGAAAGTCCATAACCCAAAGCCCAAAAGGATCACTTCTTTGAGTTCTTTGTCAATGTCAAGGAATAGTAGCTTTGATTG
CCCTCCTAAAGAAGGTTTAGATGTTCAATTGTCTAAATTCGATGGCAATGGCGATGGCGATGGCGTTGGGTTTCTTGTTGG
TAATGATCAAGATCCGGATATGGTTAATGGGTCGGACATTCAACGCCAATCTGGTTTACCAGTAGTGTCTGATCACAACAA
TACAGAGAAGCTATATGAAGAGTATCTTCAACTTCTAATCAGAACGGAAGATCTTGACCTACTTCAGTTGGATTCCTTTGC
CGAATCATTGTTGATTTGA

>VcMYBPA2a maker-VaccDscaff32-augustus-gene-55.27

ATGGGGAGAAGCCCTTGTTGCGCAAAGGAAGGGCTGAACAGAGGTTCATGGACTGCGTCTGAAGACAGGATT
CTGACTGATTACGTTAAGCTTCATGGCGAAGGTGATTGGAGAAACCTTCCCAAGAAAGCAGGTCTAAAGAGATGTGGGA
AGAGTTGTAGGCTGCGATGGCTGAATTATCTGAGGCCAGATATCAAAAGAGGGAACATATCTCCTGATGAGGAAGAGCT
TATCATTAGGCTTCACAAGCTCTTGGGCAACAGATGGTCGCTAATAGCTGGGAGGCTACCAGGCCGAACAGACAATGAAA
TCAAAAATTACTGGAACACCAATTTGTGCAAGAAGGTACAAGACCACCACTGCTCAGACAAATCTGTCAAGAATAAGAGG
AAGAAGCCTAAAATGGAGCCTAATATCGAGTCTAAAGTGGTTCGGACCAAGGCAATGAGGTGCACCAAGGTCTTTATTAG
CCCGCAGCTACAGAAAATTGAGTCAATTGATCATAACAAAGGACCACCGGTACCGGAGTCGATTTCATGCAATATTGGGT
TATCGCAATTCGCTCCGAAGGACGATAATCCGACGTTGGACGACCTCATGTTTGATGTGAATATGGAGGATGTTTTCATGT
CTGGGATTCTGAATTCGGATTTCGATGGAACGTGTTTTTTCAATTACGGCGGGGACAAATTTGACGATTTATCGGCCTCTG
TACCAGACGGAACCCTATTTTGTTCCAAGGATATGTTGGAGGATTGGACCTTAAACAATTGCCTTCAATCAAATGGGGTTG
GCTAG

>VcMYBPA2b maker-VaccDscaff33-snap-gene-307.38

ATGGACCCACATCCCAAGAAAGAATCATCCAATGTGGTCCGGACCAAGGCAGTAAGGTGCTCCAAAGTCATCA
TCAATCATCCACAGCCAAACATGAAAACTGATCATCAACCAATGGCTAAACCATTGGAAATGGTTAATGTTCAAGAAGCA
GAAGATAATAATACCTCAAGGGATTTTGCTTTGGATGCCAACATGGAGGATGTTTGGTTATCAGATTTTCTGAATTCTGAG
GAACTTCTAATGCCTTGTGAGGATACCATGGAGGATTGGAGTGGAAACAATTTCCTTGAAGAACCAAATTTAAATTATCCC
CATGGTTTGACTTCTTTTCTTGGGTATGGCGGTGATTGGCTAGCAGATAATTAA

>VcMYBR3 maker-VaccDscaff4-snap-gene-174.23

ATGTCTGAGCTCGACATCCAGATTCCTAATACCTTTGATCCATTCGCAGAGGCAAATGCTGAGGACTCGGGTGC
TGGGTCAAAGGAGTATGTGCATATCCGTATACAGCAGCGTAATGGTAGAAAAAGTCTGACCACTGTCCAAGGATTGAAG
AAGCAATTCAGCTACAATAAGATACTCAAAGACCTTAAAAAGGAGTTTTGCTGCAATGGTACTGTTGTGCAAGACCCGGA
GCTGGGTCAGATTATCCAACTTCAAGGCGATCAGCGAAAGAATGTCTCTACTTTCCTTGTTCAGGCTGGAATTGTGAAGAA
AGAACGCATCAAGATCCATGCTCGGAGGGTTGCTGGGCTTACTCCTAGGCCGGCCCTGTTTATTAGCCACACAACATGTG
CAACGAGTTACTCCAACAAGAAGATAATTGCAACAGGTCACTTTGAGATCAACAGGCTCTCCCCCTCTCTCCAAATGGCTG
ACTCAGAACACTCTTCTTCCAATGAGACTTCTGTCGATTTTCGAGATAACAAGTTCATACGACTCGACGACTTGCTTTATTT
GAGTTTGATAGTAAATGATGATGTGGGACTGTTTGTTTCAGAGGAAAGTAGCAAAGAAAAGAAACAAGAATTCTCAGAA
GACGAGGAAACACTCATCATTAGGATGTATAATTTGGTTGGAGAGAGGTGGTCTTTAATTGCTGGGAGGATTCCGGGAA
GAACAGCTGAGGAAATCGAGAAGTACTGGAACTCCAGATACTCAACGAGCGAATGA

>VcWDR1 maker-VaccDscaff28-augustus-gene-346.33

ATGGAGAATTCAACCCAAGAATCCCACCTCAGATCCGAAAACTCCGTCACTTACGACTCCCCCTACCCACTCTAC
GCCACTGCCTTCTCTTCCTCTTCCTCCCGCTCTCCCCACCACCACCGTATCGCAATCGGCAGCTTCATTGAAGAATACAACA
ACCGCGTCGATATCCTCTCTTTCGATGAAGAAACCCTAACACTCAAACCCAACCCAAACCTCTCCTTCGAACACCCTTACCC
TCCCACCAAGCTCATGTTCCACCCCAACCCCTCTAAATCCTCCGACCTCCTCGCCTCCTCCGGCGACTACCTCCGCTTATGG
GAAATCCGCGACAATAACAACTCCATCGAGCCCCTTTCCGTCCTTAATAATGCCAAAACGAGCGAGTTCTGTGCCCCGTTA
ACTTCATTCGATTGGAACGAGGTAGAGGTGAGGAGAATTGGGACGTCCAGCATCGATACCACGTGCACGATTTGGGATA

TCGAGAAAGGGGTTGTTGAAACCCAGTTGATTGCGCACGATAAGGAAGTGTACGATATCGCCTGGGGTGAAGCAGGGGT
TTTCGCATCAGTATCGGCAGATGGATCTGTGAGGATTTTCGACTTGCGTGACAAAGAGCACTCCACAATTATCTATGAAAG
CCCTACACCAGACACTCCTTTACTCCGATTGGCCTGGAACAAGCAGGATTTAAGGTACATGGCAACGATATTGATGGACTG
TAATAAGGTTGTGATATTGGATATCCGGTCACCCACAATGCCGGTGGCGGAGCTGGAGAGGCACAGGGCGAGTGTCAAC
GCGATTGCTTGGGCTCCACAGAGTAGCCGGCATATCTGTTCTGCTGGGGATGATGCGCAGGCACTCATTTGGGAGTTGCC
GACGGTGGCAGGTCCCAATGGGATTGATCCAATGTCGATGTACTCCGCCGGGTCCGAGATTAACCAGCTCCAATGGTCTG
CCAGTCTGCCTGATTGGATTGCTATTGCCTTTTCTAACAAAATGCAGCTTCTAAAAGTCTGA
